# Topographic and vegetation drivers of thermal heterogeneity along the boreal–grassland transition zone in western Canada: Implications for climate change refugia

**DOI:** 10.1002/ece3.9008

**Published:** 2022-06-22

**Authors:** Cesar A. Estevo, Diana Stralberg, Scott E. Nielsen, Erin Bayne

**Affiliations:** ^1^ 3158 Department of Biological Sciences University of Alberta Edmonton Alberta Canada; ^2^ Natural Resources Canada Northern Forestry Centre Edmonton Alberta Canada; ^3^ 3158 Department of Renewable Resources University of Alberta Edmonton Alberta Canada

**Keywords:** boreal forest, buffering, climate change, local climates, microclimate, refugia, topography

## Abstract

Climate change refugia are areas that are relatively buffered from contemporary climate change and may be important safe havens for wildlife and plants under anthropogenic climate change. Topographic variation is an important driver of thermal heterogeneity, but it is limited in relatively flat landscapes, such as the boreal plain and prairie regions of western Canada. Topographic variation within this region is mostly restricted to river valleys and hill systems, and their effects on local climates are not well documented. We sought to quantify thermal heterogeneity as a function of topography and vegetation cover within major valleys and hill systems across the boreal–grassland transition zone.

Using iButton data loggers, we monitored local temperature at four hills and 12 river valley systems that comprised a wide range of habitats and ecosystems in Alberta, Canada (*N* = 240), between 2014 and 2020. We then modeled monthly temperature by season as a function of topography and different vegetation cover types using general linear mixed effect models.

Summer maximum temperatures (*T*_max_) varied nearly 6°C across the elevation gradient sampled. Local summer mean (*T*_mean_) and maximum (*T*_max_) temperatures on steep, north‐facing slopes (i.e., low levels of potential solar radiation) were up to 0.70°C and 2.90°C cooler than highly exposed areas, respectively. *T*_max_ in incised valleys was between 0.26 and 0.28°C cooler than other landforms, whereas areas with greater terrain roughness experienced maximum temperatures that were up to 1.62°C cooler. We also found that forest cover buffered temperatures locally, with coniferous and mixedwood forests decreasing summer *T*_mean_ from 0.23 to 0.72°C and increasing winter *T*_min_ by up to 2°C, relative to non‐forested areas.

Spatial predictions of temperatures from iButton data loggers were similar to a gridded climate product (ClimateNA), but the difference between them increased with potential solar radiation, vegetation cover, and terrain roughness.

Species that can track their climate niche may be able to compensate for regional climate warming through local migrations to cooler microsites. Topographic and vegetation characteristics that are related to cooler local climates should be considered in the evaluation of future climate change impacts and to identify potential refugia from climate change.

## Graphical abstract

Characteristics that are related to colder local climates could be used for climate change adaptation. We investigated the gentle terrain in North America's boreal‐parkland‐grassland transition zone to quantify the buffering strength that local topography and vegetation can have. We found that north‐facing slopes, highly rugged terrain, and highly treed areas may be 0.5–2.0°C cooler and could be used to map key refugia areas.

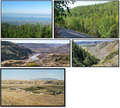

## 1 INTRODUCTION

The importance and relevance of local climates is increasingly recognized in ecological and climatological studies, particularly in a time where contemporary climate change poses threats to biodiversity and ecosystems (Hannah et al., 2014; Suggitt et al., 2018). Local climates exist at scales of meters to up to a few kilometers and are defined by the set of properties that influence atmospheric conditions at a small scale, including biotic properties (Bailey, 2009; Chen et al., 1993; Geiger et al., 1995) and topography (e.g., aspect and landform; Barry & Blanken, 2016; Thornthwaite, 1954). Local climates are thought to influence aspects of population change and community structure for a variety of organisms and biological processes, including fitness (Høyvik Hilde et al., 2016), predation (George et al., 2017), genetic diversity (Lampei et al., 2019), and species diversity (Schooler et al., 2020). Despite the potential importance of local climates, our understanding of their relevance to climate change adaptation in forests and other ecosystems is still limited.

Local climates are dictated by how physical features (physiography) influence incoming solar insulation and wind exposure and, therefore, the energy balance near the earth's surface. For instance, slopes with high sun exposure can show significantly higher temperatures of up to 7°C compared to shaded slopes (Suggitt et al., 2011). Because of changes in airflow across warm and cool slopes throughout the day in mountainous landscapes (Barry, 2008; Barry & Blanken, 2016), prevailing winds can also be more pronounced in rugged terrain, further contributing to temperature differences according to aspect (De Frenne et al., 2021; Williams & Thorp, 2015). Likewise, phenomena such as cold‐air pooling in valleys may create temperature inversions, thus decreasing local temperatures drastically (Daly et al., 2010; Nielsen & Haney, 1998). Interestingly, thermal differences driven by physical features may lead to differences in temperature with the same order of magnitude as the projected effects of climate change globally (Daly et al., 2010; Nevo, 2012).

The extent to which terrain drives local climates varies widely. Local influences can be such that local climate is buffered from regional averages (Dobrowski, 2011). In other words, terrain effects can be strong enough that local climatic trends deviate from conditions at larger (meso or synoptic) spatial scales; this has been proposed as one of the key features of climate change refugia (Dobrowski, 2011; Morelli et al., 2016; Stralberg et al., 2020). Consequently, local topography can create thermally heterogeneous landscapes that directly affect key ecological processes and patterns (Elsen et al., 2020; Swanson et al., 1988) and have the potential to reduce the exposure of biodiversity to climate extremes (De Frenne et al., 2013; Letten et al., 2013; Scheffers et al., 2014; Wolff et al., 2020). For instance, thermal heterogeneity was critical for the redistribution of many species during and after the last glacial period, particularly for disjunct populations (e.g., Fuentes‐Hurtado et al., 2016; Leipold et al., 2017), suggesting the importance of refugia for species in a contemporary climate change context. Therefore, refugia—areas that are “relatively buffered” from contemporary climate change (Morelli et al., 2016)—can provide “safe havens” for organisms against climate change (Keppel et al., 2012; Sears et al., 2011).

Vegetation may also influence local atmospheric conditions. For instance, forest cover can act in synergy with topography to influence radiation balance locally, thus affecting temperature, humidity, and wind and generally resulting in cooler local climates within the understory (Lenoir et al., 2016; Vanwalleghem & Meentemeyer, 2009). Old‐growth forests with high biomass and complexity can buffer maximum temperatures by 2.5°C relative to forests with simpler stand structure (Norris et al., 2012; e.g., plantations; Frey et al., 2016) and can be about 5°C cooler than areas with less forest cover (Davis et al., 2019). Meanwhile, forest canopies retain heat in the winter, resulting in warmer temperature under the canopy relative to non‐forest areas, especially in boreal regions (De Frenne et al., 2019). Thus, forests can buffer local climates against both extremely warm and cold temperatures.

Local climates have been investigated extensively in mountainous regions and mountain basins, where topographic effects (from varied terrain and elevation) are most pronounced (e.g., Cantlon, 1953; Clements et al., 2003). In mountainous areas, local changes in elevation provide excellent “natural experiments” for ecological and meteorological studies, with a diversity of gradients, including radiation, humidity, precipitation, and temperature. Elevation differences had also been used to identify climate change refugia (Ashcroft et al., 2012). However, elevation per se is a poor predictor of climate at smaller scales because air temperatures near the ground may not be correlated with temperatures in the free atmosphere (Dobrowski, 2011; Lookingbill & Urban, 2003). This suggests that temperature predictions that are solely based on temperature changes with elevation (adiabatic lapse rates) do not include important topographic and vegetation effects on local climatic conditions. Therefore, incorporating finer scale features such as aspect, landform, and forest cover can substantially improve our predictions of the local climate.

In landscapes with gentle terrain, thermal heterogeneity and seasonal attenuation of minimum and maximum temperatures (i.e., climatic buffering) should be more limited compared to mountainous landscapes, as the strength of influence of topographic factors should be smaller (e.g., Keppel et al., 2017). The velocity required for organisms to track their climate niche as the climate changes is also greater in flatter areas, relative to mountains where climatic gradients are steeper, suggesting that flatter areas might be more susceptible to rapid changes in climate (Barber et al., 2016; Carroll et al., 2015; Loarie et al., 2009). In the boreal plains region of Western North America, thermal heterogeneity in river valleys and hill systems may result in local climates that are buffered from regional temperature increase. Relatively cooler (and thus wetter) conditions could be critical for retaining boreal forest tree species, especially moisture‐limited conifers such as white spruce (*Picea glauca*; Hogg, 1994). The remaining forest patches could further cool local conditions through canopy shading and associated temperature buffering (De Frenne et al., 2021). The resulting refugia can provide habitat for forest‐dependent plant and wildlife species and serve as “stepping stones” to facilitate climate‐driven range shifts (Hannah et al., 2014; Stralberg et al., 2015).

**FIGURE 1:**
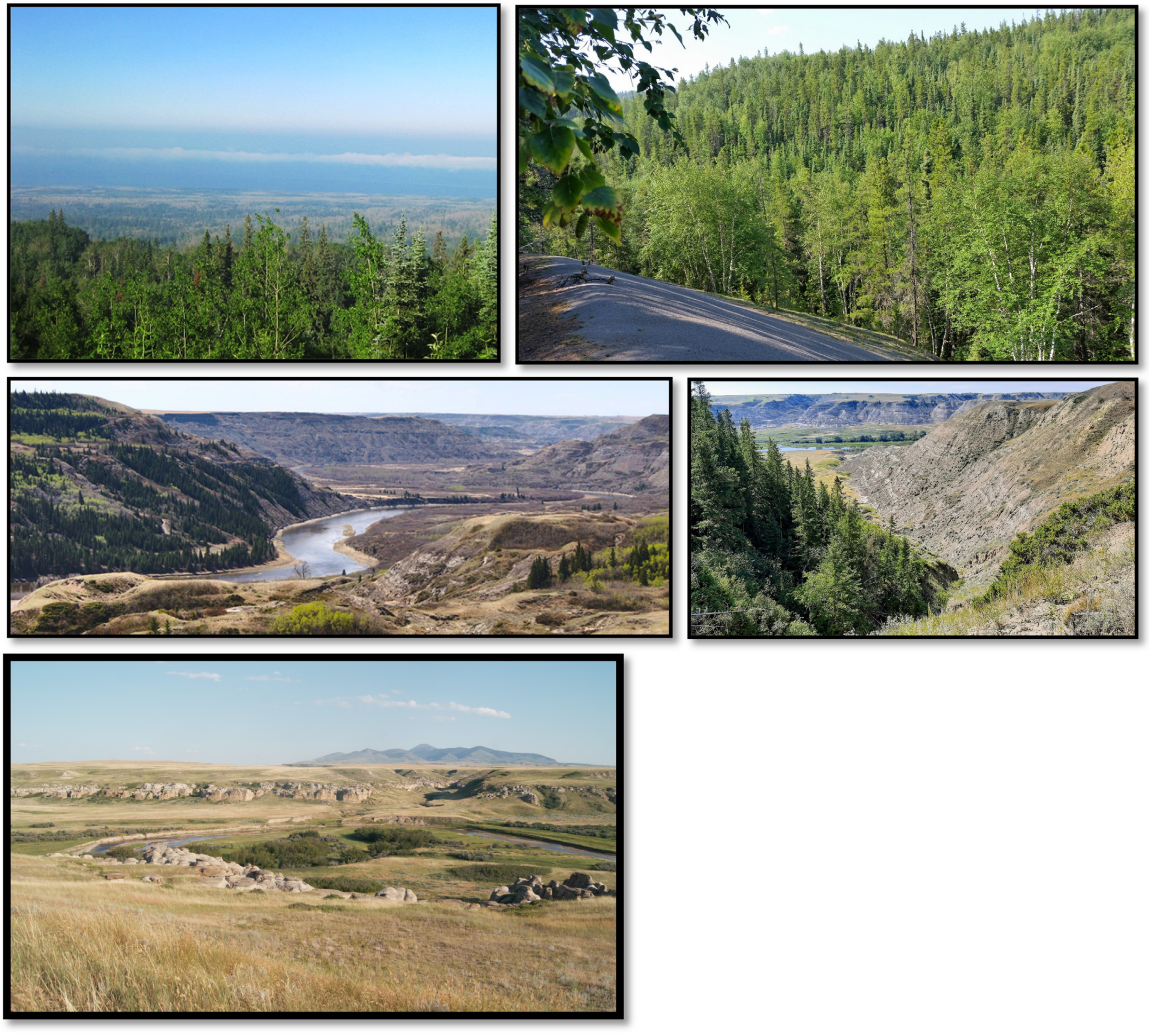
Examples of ecosystems and contrasting slopes sampled in Alberta, Canada. Writing‐on‐Stone Provincial Park with a tree patch at valley bottom (bottom; ~49° N); contrasting slopes with remnant conifer forests in river valley systems at Dry Island Buffalo Jump Provincial Park (center left) and Tolman Badlands Heritage Rangeland Natural Area (center right) in South‐Central Alberta, Canada (~52° N); hills systems at Marten Hills (top left) and Watt Mountain (top right) in Central (~55° N) and Northwest (~59° N) Alberta, respectively

The boreal forest is expected to experience northward shifts of entire ecoregions (Rehfeldt et al., 2012), with the largest changes in vegetation expected at southern margins where higher evapotranspiration and incidence of drought and heat stress are expected to surpass biological thresholds (Price et al., 2013; Schneider, 2013). In much of the western prairie province of Alberta, Canada, the difference between precipitation and evapotranspiration is close to zero, resulting in the potential for local differences in vegetation. In the prairie part of the province, patches of trees consisting of species typically associated with boreal forests persist along north‐facing slopes in river valleys and at higher elevations (Figure 1). Most notably, the Cypress Hills of southern Alberta contains one of the few larger isolated remnants of coniferous species (white spruce and lodgepole pine *– Pinus contorta*) in the Canadian prairies because of cooler temperatures at higher elevations. These forests were likely established during the retreat of the previous ice sheet when boreal mixedwood forests occupied much of what today are the grassland landscapes of southern Alberta (Dyke, 2007; Moss, 1955; Strong & Hills, 2005). These ecological remnants provide contemporary analogs for what northern boreal forest landscapes may resemble in a warmer and drier future. Therefore, we view boreal forest refugia as areas in which topographic effects lead to cooler local climates that allow coniferous trees, particularly white spruce, to persist over time.

We sought to understand the role that fine‐scale variations in local topography and vegetation play in promoting thermal heterogeneity. Moreover, we wanted to quantify the degree to which topography and vegetation can reduce or buffer temperatures in the boreal–grassland transition zone of Western Canada. Specifically, we investigated the effects of different terrain features (including elevation) and vegetation cover types on minimum, mean, and maximum monthly temperatures during summer and winter seasons when extreme temperature values are most likely. We defined temperature buffering as some combination of decreasing mean and maximum temperatures during summer warm months, increasing mean and minimum temperatures during winter cold months, and/or decreasing temperature ranges in both seasons. In addition, we investigated the extent to which a standard gridded climate product—based on interpolated weather station data and downscaled as a function of elevation‐derived lapse rates—captures thermal heterogeneity. We did so by monitoring and analyzing climate conditions in several river valleys and hill systems along a 1000+ km latitudinal gradient in Alberta, Canada. Our survey design covered vegetation ranging from isolated boreal forest remnants within landscapes currently dominated by grassland in the south to contiguous boreal conifer and mixedwood forest in the northern reaches.

## 2 METHODS

### 2.1 Site selection and study areas

**FIGURE 2:**
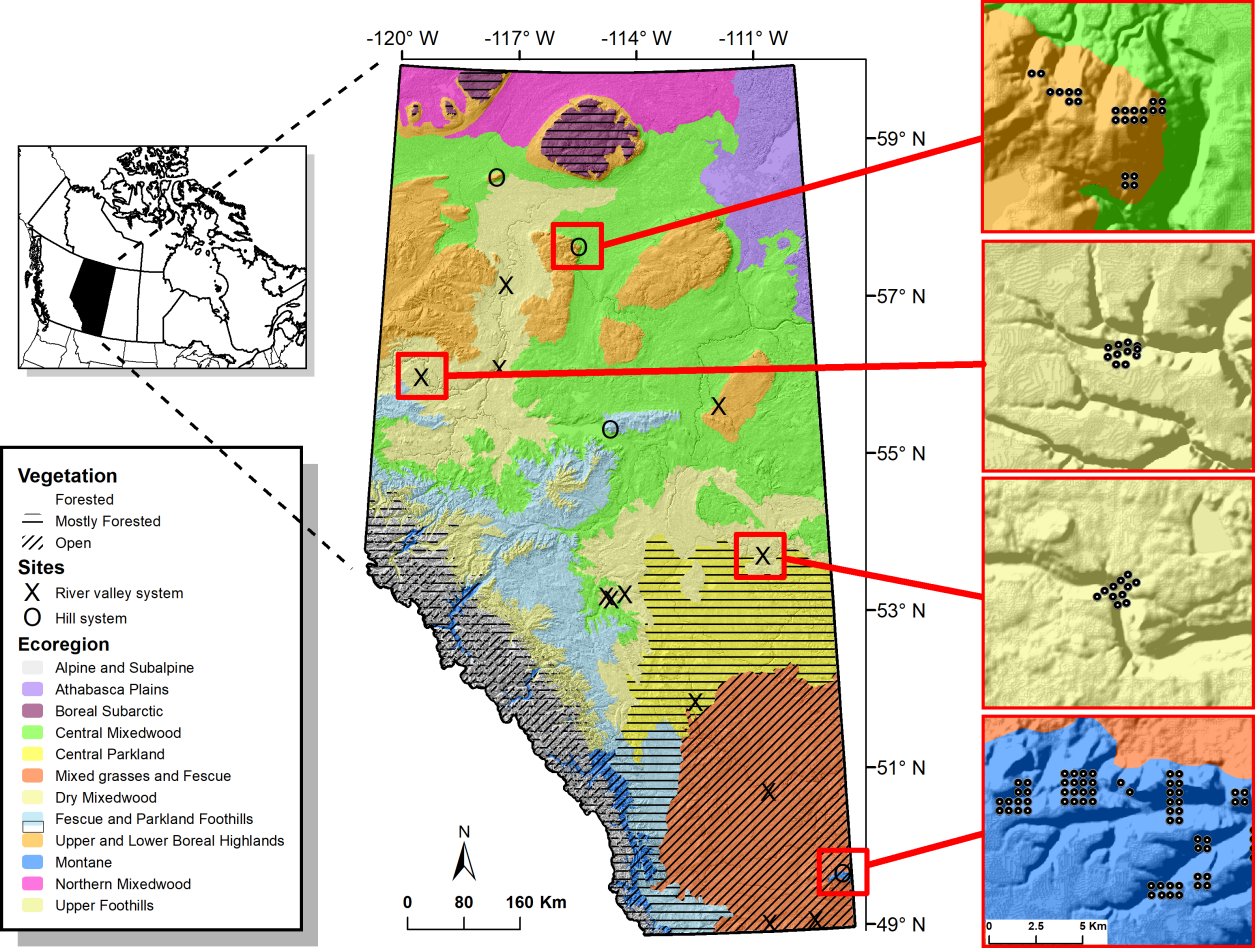
Location of sample sites (river valley and hill systems) in Alberta, Canada, with different sub‐ecoregions in the province. Some classes were grouped for mapping. Northern portions of Alberta are often times composed of open wetlands interspaced by trees; therefore, this simplified version may not necessarily represent entire sub‐ecoregions. The map is overlaid on a hillshade model to depict topography across study sites. The column on the right depicts examples of the sampling scheme of some iButtons (black dots) in some valleys and hills systems

This study encompassed four hills and 12 river valleys systems along a latitudinal gradient in Alberta, Canada, that covers a transition from boreal forest to parkland to grassland ecosystems (Figure 2). The parkland natural region is a transition between grassland and boreal forests and consists primarily of aspen (*Populus tremuloides*) and grassland mosaic interspersed with occasional balsam poplar (*Populus balsamifera*) and white spruce forests (*Picea glauca*). Hill and valley formations in Alberta are a result of differential fluvial erosion of sedimentary bedrock in the Western plains during the Quaternary glaciations. Existing hills systems are upland remnants more resistant to erosion, whereas river valleys are, for the most part, remains of pre‐glacial rivers that were filled with Quaternary sediments (Fulton, 1989). Such pre‐glacial valleys are prominent in northern regions of Alberta, particularly in between boreal highlands (Figure 2). With the retraction of the Laurentide Ice Sheet (18–11 ka), ecoregions and biomes that were once pushed farther south expanded northward (Dyke, 2007), with some vegetation remaining along climatically suitable areas in central Alberta. Cypress Hills, in southeast Alberta, was one of the areas that remained unglaciated throughout the Quaternary period (Fulton, 1989). We sampled similar upland vegetation across hill and valley systems, which consisted mostly of white spruce (*Picea glauca*), trembling aspen (*Populus tremuloides*), birch (*Betula* spp.), and balsam poplar (*P. balsamifera*), as well as plains cottonwood (*P. deltoides*) in valley bottoms of southern sites. The Cypress Hills site (farthest south and considered part of the Rocky Mountain natural region in Alberta) also included lodgepole pine (*Pinus contorta*). Some valleys were treeless on south‐facing slopes, creating a sharp contrast with forested north‐facing slopes (Figure 1).

Normal climatic conditions in our study sites vary widely. At colder and wetter sites in the north (55° N through 59° N), mean annual temperatures ranged from −2.7 to 1.4°C (30‐year normal; 1961–1990), and precipitation ranged from 256 to 281 mm per year. The central and southern regions (49° N through 55° N) experienced long dry and hot periods during summer and warmer temperatures during winter. In central Alberta, mean annual temperatures at study sites ranged from 1.6 to 2.7°C and precipitation from 386 to 432 mm. In the southmost river valley sites, the mean annual temperature was approximately 5°C, and the mean annual precipitation hovers around 315 mm. In Cypress Hills, the southernmost hill system, the mean annual temperature was slightly cooler and precipitation slightly higher (~2.5°C and 435 mm, respectively).

Current differences in mean annual temperature in Alberta are approximated by changes in latitude (inverse relationship) along the boreal–parkland–grassland gradient (Figure S1). We identified four mean annual temperature strata (hot–warm–cool–cold) within Alberta and selected accessible hill and valley systems (i.e., up to 5 km of a road) within each stratum for field sampling along the boreal–parkland–grassland gradient. We avoided the Rocky Mountain foothills, which are much wetter and less seasonal than boreal environments, and also contain different floristic communities. We selected sites mostly within protected areas to reduce confounding factors caused by human activities (i.e., forest clearing). Once we identified hills or valley systems, we placed iButton temperature data loggers (details below) at a spacing of at least 500 m along elevational gradients by either setting up transects along the elevation gradient or by placing a 500‐m virtual grid over the area with iButtons at the junctions of the grid. We attempted to achieve equal coverage of distinct landforms, that is, ridgetops, valley bottoms, and slopes with large aspect contrasts (i.e., northeast and southwest facing), reflecting differences in solar radiation. We purposely selected river valleys that were representative of the existing distribution of trees in river valleys of the sites in the grasslands ecoregion. For instance, sites in the far south (~49° N) were either treeless or contained trees only in valley bottoms (Figure 1, bottom). In the grassland ecosystem (mixed grasses and fescue biomes) and central parkland, patches of trees were more common in valley bottoms and generally occurred on north‐facing slopes (Figure 1, center left and right). Further north in the boreal and central mixedwood biomes ecosystems, trees were present throughout (Figure 1, top left and right).

### 2.2 Temperature data logger deployment and sampling

We deployed 283 iButton temperature data loggers (Thermochron iButton model DS1922L; N_Hills_=166 and N_Valleys_=117) between May and June 2014 (hill systems) and 2018 (river valleys), with some river valley stations deployed in September 2018. We programmed iButtons to sample every 90 min and retrieved them approximately 1–3 years after deployment (July–August 2015 – Watt Mountain [~59°N] and Buffalo Head Hills [~58°N]; May–June 2016 for Cypress Hills [~49.5°N]; June–July 2017 for Marten Hills [~55°N]; and August–September 2020 for river valleys). Fourteen iButtons from hill systems and 29 from river valleys either failed or were damaged by wildlife, leading to a final sample size of *N =* 240. For each iButton, we built inexpensive radiation shields following the procedures of Holden et al. (2013). Radiation shields have been reported to be comparable with weather stations, with a small warm bias of up to 1°C (Holden et al., 2013; Terando et al., 2017). This allowed us to compare temperatures from data loggers directly with weather station‐derived estimates, such as ClimateNA (see details below). We attached each shield, with its enclosed iButton, to the north‐facing side of a tree at 1.5 m from the ground or to wooden stakes approximately 1.5 m above the ground in treeless areas. We removed obviously unrealistic iButton logger values (high and low), that is, where temperature sensors failed, by excluding values outside of ±3 times the interquartile range for all summer months. This allowed us to use only reliable temperature measures and thus remove bias in our analysis since all metrics were summarized to monthly averages. We discarded data for the month of deployment or retrieval for sampling stations if it had less than 20 days of sampling. We imputed daily temperature data for the stations that had missing days by using univariate time series imputation with spline interpolation within the *imputeTS* package in R (Moritz & Bartz‐Beielstein, 2017). This approach allowed us to estimate monthly temperature metrics in a way that respected the seasonality of a given month (e.g., colder temperatures at the end of a summer season).

### 2.3 Temperature metrics

Following Suggitt et al. (2011), we calculated five temperature metrics from the raw data for each month: average of daily maximum (*T*_max_), minimum (*T*_min_), and mean temperatures (*T*_mean_), growing degree days above 5°C (GDD_5)_ and average of the daily temperature range (*T*_range_). In addition to these metrics, we also calculated the 99^th^ percentile of daily maximum temperatures (*T*_99_) to evaluate topographic effects on extreme temperature events. We chose these metrics based on their relevance to several ecological processes, such as animal and plant thermoregulation, plant recruitment, animal distribution, and because of their relevance in identifying climate change refugia from a temperature standpoint (Ashcroft et al., 2012; Briga & Verhulst, 2015; Dobrowski, 2011). Our next step was to subset the data into two seasons consisting of data from June, July, and August (northern hemisphere summer season), and December, January, and February (winter season). These two seasons are ecologically relevant for several reasons. The summer period is crucial for several taxa as it corresponds to the growing season for plants and the breeding season for most animals. In addition, the effects of climate change are expected to be more pronounced during summer, with higher maximum temperatures and more extreme drought periods, but also during winter, with higher minimum temperatures and increased frost‐free periods (Price et al., 2013). We gave particular attention to *T*_max_, *T*_99_, and GDD_5_ for the summer season as these metrics are more directly driven by the warmer season, while we calculated *T*_mean_ and *T*_range_ for both summer and winter, and *T*_min_ for the winter (refer to the Appendix S1 for results of all metrics in both seasons). Such an approach allowed us to consider our results in view of the buffering effects that forest cover and terrain could have on local climates (i.e., lower summer *T*_max_ and *T*_mean_ and warmer winter *T*_min_; De Frenne et al., 2019). The summer temperature metrics considered here are particularly relevant from a boreal refugia perspective because of their direct linkage to conditions that could be favorable (cool and wet) or unfavorable (hot and dry) to seedling development and recruitment of coniferous trees, especially white spruce (*Picea glauca*; Hogg, 1994; Price et al., 2013). Therefore, in analyzing the effects of topographic variables on local climates, we focused on coniferous boreal trees, especially white spruce (*Picea glauca*).

Finally, we calculated the correlation between temperature metrics to assist with model interpretation.

### 2.4 Topographic and vegetation variables

We used a suite of topographic and vegetation variables that could affect climate conditions at the local scale: solar radiation, topographic roughness index (TRI), landform, elevation, latitude, compound topographic index (CTI), and vegetation cover (Table 1). Topographic variables were calculated from a 50‐m digital elevation model (DEM) derived from 1:50,000 Topographic Data of Canada (CanVec series).

**TABLE 1:** Topographic and vegetation variables used in temperature regression of temperature sensors deployed in hills and river valley systems in Alberta, Canada

Category	Variable	Definition	Related literature/Source
Topography	Elevation	Derived from a 50‐m DEM	
	Solar Radiation	Based on nonparametric multiplicative regression using slope, aspect, and a constant latitude of 53 N	McCune (2007)
	Landform	Valley or ridge top based on topographic position index of a 300 m radius and a slope grid	Jenness (2006)
	TRI	Topographic roughness index, as the sum change in elevation in the eight neighboring cells	Riley et al. (1999)
	CTI	Compound topographic index, calculated based on flow direction, accumulation, and slope derived from a 50‐m DEM	Rho (2002); Nielsen et al. (2004)
Vegetation	Forest Cover	Percentage of forest cover around each iButton station on a 3 × 3 50 m pixel moving window	ABMI (2010)

We estimated annual potential relative solar radiation (in MJ/cm^2^/year; hereafter solar radiation) by using a multiplicative kernel smoothing technique that uses slope, aspect, and cumulative warming from the afternoon sun following equations from McCune and Keon (2002) and McCune (2007). Despite not being a direct measure of solar radiation, this modeled terrain‐based estimate should reflect the effects of slope and aspect on local climates. We decided to use a constant midpoint latitude in this case so that we could model the effects of latitude separately in our models (see Analysis section).

We calculated the terrain roughness index (TRI) as the sum of the change in elevation between a given grid cell and its surrounding cells, which indicates the level of topographic heterogeneity in a certain area (Riley et al., 1999). The compound topographic index (CTI) tracks the flow of water drainage and could be used as a proxy for cold‐air drainage, soil moisture, and topographic evenness (Daly et al., 2010; Dobrowski, 2011; Lookingbill & Urban, 2003). We calculated CTI by using the spatial analyst extension in ArcView 3.2 and a script developed by Rho (2002). To generate landform classes, we first calculated the topographic position index (TPI) using a circular radius of 300 m and a slope raster to generate landform grids further categorized into 10 classes (Jenness, 2006). For this study, we extracted whether the station was located at an incised valley or ridge top by using the first and last class generated when calculating landform. We grouped the other classes into a single one, as their effect is likely represented in the other topographic variables. For vegetation, we summarized forest cover from the spatial land cover inventory layer developed by the Alberta Biodiversity Monitoring Institute (‘Wall‐to‐wall Land Cover Map’ version1.0 from 2010 retrieved from http://www.abmi.ca, ABMI, 2010). We extracted broadleaf, conifer, and mixedwood polygon layers from the land cover map and rasterized them to a 50‐m resolution. We used a moving window of 3 × 3 cells to calculate the percent cover of vegetation in the surrounding landscape. We used these same layers for mapping purposes and comparison with ClimateNA (see analysis part below). Collinearity was not an issue with these covariates, as all the variables in our analysis had reasonably low correlations (Pearson *R* ^2^ < 0.7, Figure S2) and were all included in our analysis.

### 2.5 Analysis

#### 2.5.1 Effects of topographic factors and vegetation on local climate

We used three different approaches to evaluate the effects of topography and vegetation cover on the local climate. First, we standardized all variables to facilitate the assessment of effect sizes. We compared a set of a priori models and hypotheses (Table 2) with different variable combinations using the Akaike Information Criterion to rank models (AIC; Burnham & Anderson, 2004). Our main objective was to evaluate the amount of support for models that included only elevation against models that incorporated additional topographic and vegetation effects. Thus, all models in this step include elevation as an environmental null model. The *aspect model* was used to evaluate the importance of differences in solar radiation associated with slope and aspect. With the *topodiversity* model, we wanted to know whether roughness and topographic diversity were important, whereas the *topodiversity and vegetation effects* models also included the percentage of broadleaf, conifer, and mixedwood canopy cover around the station as an additive effect. The *moisture and landform* model was used to test the level of support for the potential effects of soil moisture and topographic position based on CTI and landform classes. Here, we emphasized models for summer *T*_max_ and *T*_mean_, and winter *T*_mean_ and *T*_min_ (please refer to the Appendix S1 for models for all metrics in both seasons). We included latitude in all models to control for the overarching influence of latitude on temperature.

**TABLE 2:** Models developed to compare different effects of local topographic features and vegetation cover on different temperature metrics and related hypotheses. All models also included latitude as an additional variable (see Analysis section)

Model	Variables	Mechanism	Hypothesis	Expectation
Elevation	Elevation	Adiabatic cooling	Adiabatic lapse rates (i.e., elevation) are the predominant factor regulating temperature in hills and valleys	Decrease in temperature with increasing altitude; expected negative effects across temperature metrics
Aspect	Solar radiation	Increased/decreased solar radiation	Heating from incoming solar radiation due to aspect and terrain slope lead to warmer local climates in highly exposed slopes, and cooler local climates in areas with low exposure	All temperature metrics are expected to increase with increasing sun exposure. Conversely, topographic shading has a lower temperature. *T*_range_ is expected to increase
	Elevation	Adiabatic cooling		Decrease in temperature with increasing altitude; expected negative effects across temperature metrics
Topodiversity	Solar radiation	Increased/decreased solar radiation	Terrain ruggedness, through increasing air motion and mixing, and incised valleys, through cold‐air pool, are cooler, whereas ridges are warmer; aspect and slope lead to warming (high exposure) and cooling (low exposure). Local climates are highly heterogeneous	All temperature metrics are expected to increase with increasing sun exposure; shaded areas are cooler
	Elevation	Adiabatic cooling		Decrease in temperature with increasing altitude; expected negative effects across temperature metrics
	Landform	Temperature inversion and cold‐air pooling		Incised valleys are expected to have lower *T*_min_ and *T*_range_ as a result of cold‐air pooling, whereas ridge tops are warmer.
	TRI	Air motion and mixing		Lower temperatures are expected in highly rugged areas
Moisture and Landform	Elevation	Adiabatic cooling	Soil moisture potential and drainage lead to different local climates; valleys are colder, whereas ridges are warmer due to cold‐air pool formation and exposure, respectively	Decrease in temperature with increasing altitude; expected negative effects across temperature metrics
	CTI	Drainage and wetness		Wetter areas (i.e., high soil moisture potential) are expected to have higher *T*_min_ and lower *T*_max_/*T*_99_;
	Landform	Temperature inversion and cold‐air pooling		Decrease in temperature with increasing altitude; expected negative effects across temperature metrics
Topodiversity and Vegetation Effects	Solar Radiation	Increased/decreased solar radiation	Local climates are highly heterogeneous, driven by roughness (air motion), solar exposure, and landforms (e.g., cold‐air pools and exposure); local climates below forest canopies are more moderate, with lower daily variability	All temperature metrics are expected to increase with increasing sun exposure; shaded areas are cooler
	Landform	Temperature inversion and cold‐air pooling		Incised valleys are expected to have lower *T*_min_ and *T*_range_ as a result of cold‐air pooling, whereas ridge tops are warmer
	TRI	Air motion and mixing		Lower temperatures are expected in highly rugged areas
	Elevation	Adiabatic cooling		Decrease in temperature with increasing altitude; expected negative effects across temperature metrics
	Vegetation	Canopy buffering		Canopy cover is expected to decrease *T*_max_/*T*_99_, increase *T*_min_, and decrease *T*_range_. The coniferous cover is expected to have stronger effects (i.e., cooler)
Full	CTI	Drainage and wetness	Topographic diversity and local vegetation (topodiversity and vegetation effects hypothesis), in addition to soil moisture potential (moisture and landform hypothesis), create highly heterogeneous thermal landscapes	Wetter areas (i.e., high soil moisture potential) are expected to have higher *T*_min_ and lower *T*_max_/*T*_99_;
	Solar Radiation	Increased/decreased solar radiation		All temperature metrics are expected to increase with increasing sun exposure; shaded areas are cooler
	Landform	Temperature inversion and cold‐air pooling		Incised valleys are expected to have lower *T*_min_ and *T*_range_ as a result of cold‐air pooling, whereas ridge tops are warmer.
	TRI	Air motion and mixing		Lower temperatures are expected in highly rugged areas
	Elevation	Adiabatic cooling		Decrease in temperature with increasing altitude; expected negative effects across temperature metrics
	Vegetation	Canopy buffering		Canopy cover is expected to decrease *T*_max_/*T*_99_, increase *T*_min_, and decrease *T*_range_. The coniferous cover is expected to have stronger effects (i.e., cooler)

Secondly, we developed full models with additive effects for all variables mentioned in the previous section to develop spatially explicit predictions to compare with another gridded temperature product (ClimateNA; see details in the next section). We evaluated the significance, direction, and strength of influence of β‐coefficient estimates to interpret the effects of each covariate on the variation of local temperature. We used general linear mixed effect models with the monthly temperature metrics as response variables for all models. We developed separate model sets for the winter and summer seasons and fit models using each hill or river valley system as a random intercept to account for latent climatic phenomena and properties of each system. We added an additional random effect for the year and month of sampling. We also incorporated a within‐group correlation structure to account for temporal autocorrelation within each season by using a continuous autoregressive process (corCAR1) and a constant variance function structure with month as a grouping factor. We performed all modeling within the R environment (R Core Team, 2013) using the *lme* function from the *nmle* package (Pinheiro et al., 2007) for linear mixed effect models.

Finally, we calculated conditional and marginal coefficients of determination (the proportion of variance explained by fixed and random effects, *R* ^2^_c_, and fixed effects only, *R* ^2^_m_, respectively) to examine the goodness‐of‐fit of our models using the *squaredGLMM* function in the *MuMIn* package (Barton, 2020; Nakagawa & Schielzeth, 2013). We also evaluated the improvement in the explanatory power of models that incorporate all topographic variables (*full model*) relative to the *elevation* model by measuring the percent change in *R* ^2^_c_.

#### 2.5.2 Mapped local climate and comparison with ClimateNA

We used the full models based on iButton data from step two in the previous section, but with non‐standardized variables, to generate spatially explicit predictions of summer *T*_mean_ and *T*_max_. We then constrained our mapped predictions to a region of approximately 25 × 25 km around each river valley or hill system, thereby avoiding predicting outside the range of our data. To illustrate our results based on iButtons and to compare with other gridded products, we also created climate grids using ClimateNA, a graphical user interface package that provides climate predictions at different scales (Wang et al., 2016). The interface provides data in different temporal scales (e.g., monthly, yearly, and seasonally) when provided with either point coordinates or a raster DEM. The package uses monthly temperature data for the normal period of 1961–1990 as a baseline, which is compiled from four different sources depending on the region. For Alberta, it resamples PRISM grids to a 4‐km resolution baseline by using bilinear interpolation and empirical lapse rate with high accuracy (Wang et al., 2016). In recent years, ClimateNA retrieves historical climate data generated by the Climate Research Unit at the University of East Anglia, which is updated yearly. Comparing ClimateNA to our iButton predictions is advantageous as the former provides scale‐free climate estimates and empirical lapse rates that should be consistent with the ones in our iButton predictions. We generated spatial temperature estimates with ClimateNA (version 6.3) for the same 25 × 25‐km region and same sampling period as the iButtons (2014–2020) by supplying the ClimateNA software with a DEM at 50‐m resolution. We then extracted monthly average temperature data from ClimateNA for the same location, month, and corresponding year of iButton data. Even though iButtons and ClimateNA predictions differ in terms of precision and range of values, the difference between the two rasters should remain constant across elevational gradients in the absence of topographic effects. However, the dissimilarity should be higher at valley bottoms, mountain tops, and in areas with high or low incoming solar radiation, which are factors that are not necessarily captured by interpolated products. Therefore, our goal was to quantify the underlying drivers for the expected difference between ClimateNA and direct iButton readings. We quantified the magnitude of the difference between ClimateNA and iButton measurements as the absolute difference between ClimateNA grid cell values extracted and the iButton measurement for that station (summarized as monthly *T*_max_, *T*_min_, GGD_5_, and *T*_mean_ averages). We modeled the absolute difference as a function of the full model from Table 2 for the summer and winter seasons but included an interaction term between solar radiation and the percentage of vegetation cover to account for warmer and treeless slopes. We used the same random effect and correlation structure from the models detailed in the previous section and evaluated the effect size and significance of each variable in explaining the differences. Finally, we generated temperature surfaces based on ClimateNA and iButtons for July 2018 and calculated the absolute difference between the two data sources for illustrative purposes.

## 3 RESULTS

### 3.1 Effects of topographic factors and vegetation on local climate

Models that included all predictor variables (*full* model) were among the top three models in both summer and winter for all metrics (Table 3) and were always among the models with the most support (ΔAICc < 2), except for summer *T*_range_ and winter GDD_5_ (see Table S1), Models that accounted for tree cover, solar radiation, topographic roughness, and landform variables, in addition to latitude and elevation (i.e., the *topodiversity and vegetation effects* model), received either similar or more support than the full model for most metrics in both seasons. Overall, models that included additional topographic features, besides elevation, received substantially more support than elevation‐only models in both seasons. However, adding a terrain wetness term (i.e., CTI) did not improve models substantially and the *Moisture and Landform* model received little support throughout the analysis. Notably, the correlation between temperature metrics within seasons was high (*R* ^2^ > 0.7, Figure S3) for most metrics, except for summer *T*_min_ (*R* ^2^ < 0.7) and in some cases for *T*_range_. In most cases, the ranking for the full model was similar, but the level of support could vary substantially. For example, summer *T*_max_ and summer *T*_range_ were strongly correlated, but the support for the full model was ΔAICc < 2 for summer *T*_max_ and ΔAICc > 2 for summer *T*_range_ (Table S1), suggesting that topographic and vegetation effects on summer *T*_range_ are largely driven by their effects on summer *T*_max_.

**TABLE 3:** Model ranking for two different temperature metrics for the summer months between 2014 and 2020 in the river valley and hill systems in Alberta, Canada. Only the top three models are presented for *T*_max_ and *T*_min_. Please refer to Table 2 for variables in each model. K *–* number of parameters, ω *–* weighted AICc of the model, LL *–* negative log‐likelihood. ΔAICc *–* difference in AICc between a given model and the top model of that model set

Season	Metric	Model	K	ΔAICc	ω	LL	*R* ^2^_m_	*R* ^2^_c_
Summer	*T*_max_	Topodiversity and Vegetation	17	0.00	0.55	−2451.64	0.39	0.84
		Full	18	1.42	0.27	−2451.32	0.39	0.84
		Topodiversity	14	2.28	0.18	−2455.86	0.39	0.84
	*T*_mean_	Topodiversity and Vegetation	17	0.00	0.73	−1469.74	0.37	0.95
		Full	18	1.99	0.27	−1469.71	0.37	0.95
		Topodiversity	14	24.29	0.00	−1484.96	0.34	0.95
Winter	*T*_mean_	Full	18	0.00	0.99	−2095.69	0.18	0.91
		Topodiversity and Vegetation	17	8.54	0.01	−2100.99	0.18	0.91
		Topodiversity	14	26.18	0.00	−2112.91	0.16	0.91
	*T*_min_	Full	18	0.00	1.00	−2487.68	0.11	0.85
		Topodiversity and Vegetation	17	12.03	0.00	−2494.73	0.11	0.85
		Moisture and Landform	13	19.26	0.00	−2502.46	0.09	0.84

**FIGURE 3:**
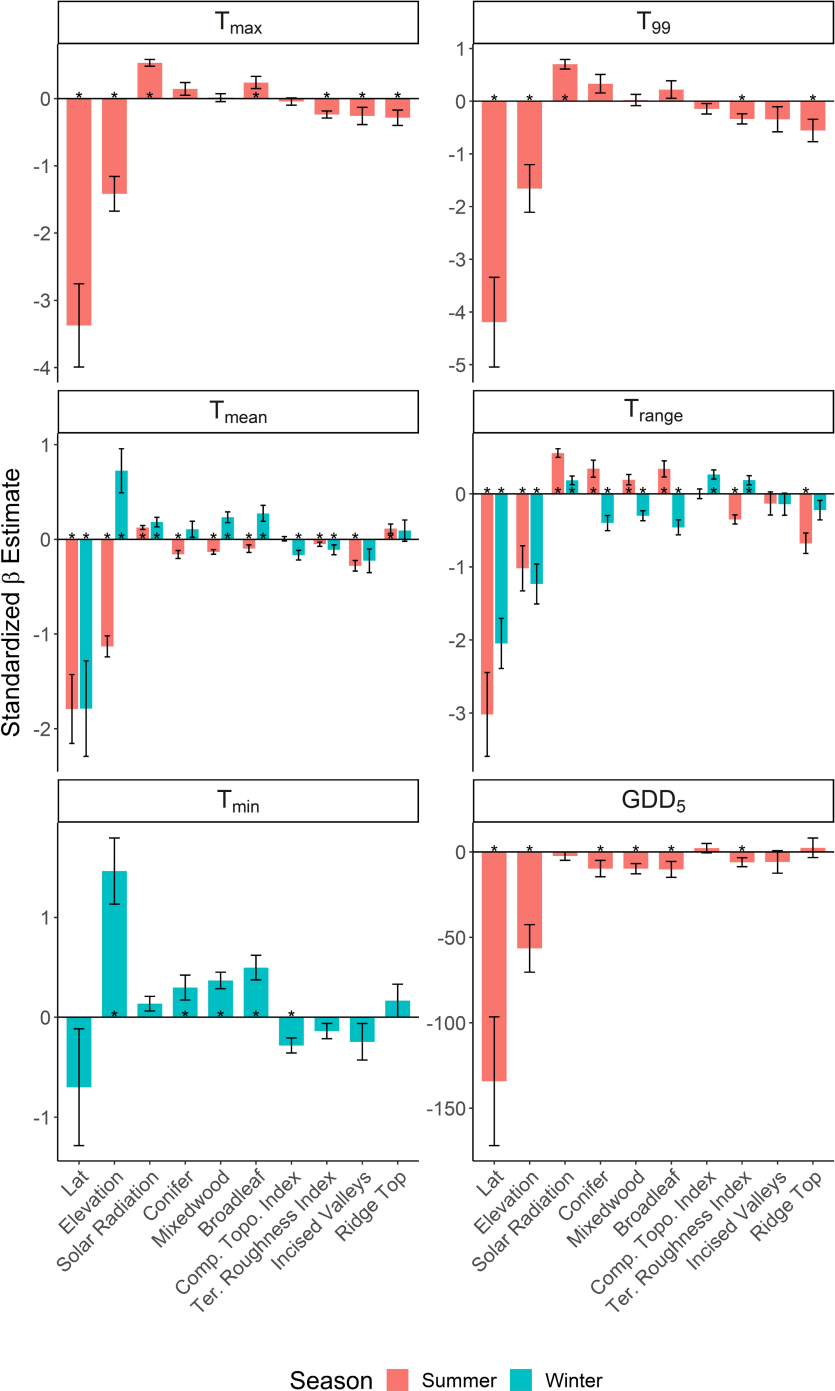
The influence of topographic and ecological variables over the monthly average of daily *T*_max_, *T*_min_, *T*_mean_, the 99th percentile of daily maximum temperatures (*T*_99_), growing degree days above 5°C (GDD_5_), and average of the daily temperature range (*T*_range_) for the summer and winter seasons in the river valley and hill systems in Alberta, Canada. Standardized beta coefficients are from the full model (refer to the Methods section and Table 2 for more details). See Figure S4 for results of other temperature metrics. Error bars represent standard errors and * indicates significant estimates at ɑ = 0.05

The direction of each variable's effect remained relatively consistent across all temperature metrics and seasons (Figure 3, Figure S4). In terms of the effect size of each variable relative to elevation, we found, as expected, that latitude was the strongest individual predictor for most metrics, except for winter *T*_min_, for which elevation was the strongest (positive) predictor. Other topographic and vegetation variables had a smaller influence on temperature metrics when compared with elevation. For instance, solar radiation increased summer high‐temperature extremes (*T*_max_ and *T*_99_) and temperature range (*T*_range_), with a combined effect size that was approximately 37–54% of the effect size of elevation. Aspect‐ and slope‐driven increases in solar radiation also increased summer mean temperature (*T*_mean_), but with a smaller effect (~25%). Overall, the directionality of effects was similar over the winter, but with a lower magnitude.

Terrain roughness (TRI) significantly decreased high‐temperature extremes (*T*_max_ and *T*_99_), mean temperatures (*T*_mean_), and the range of temperatures (*T*_range_) during the summer, but its effect size was much smaller than that of elevation (~4–24%). Our proxy for soil wetness had no effects on summer temperature but decreased minimum and average temperatures and the range of temperatures in the winter to a minor degree. Summer temperature decreased significantly in incised valleys, particularly for *T*_mean_ (~25% of the effect size of elevation).

Overall, the amount of surrounding forest cover for all three types had significant negative effects on most summer temperature metrics, except for high extremes (*T*_max_ and *T*_99_), where the broadleaf cover had even positive effects (Figure 3 and Figure S4). The effects of vegetation cover on winter temperatures were positive for *T*_min_ and *T*_mean_, suggesting a buffering effect. Out of the three different vegetation types analyzed, coniferous forest cover had the strongest (negative) effect on average summer temperature (*T*_min_ and *T*_mean_), particularly compared to broadleaf forest.

**FIGURE 4:**
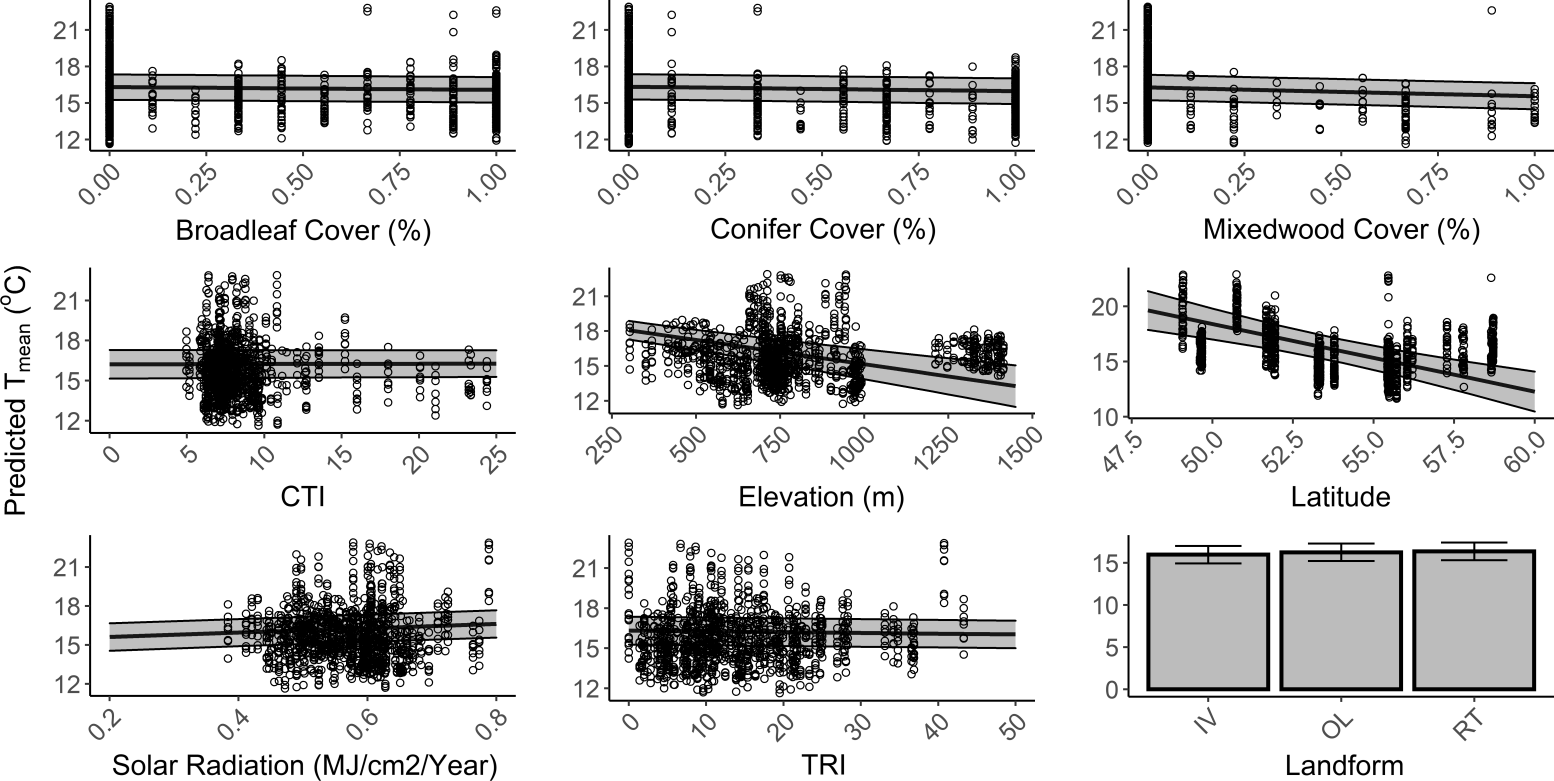
Predicted effects of topographic and vegetation variables (non‐standardized) sampled on summer *T*_mean_ for July 2018 in hill and river valley systems in Alberta, Canada. Shaded areas around the regression line represent 95% confidence intervals. Landforms: IV – incised valleys, RT – ridge tops, OL – other landforms. See Figures S4 and S5 for coefficients of other temperature metrics and other seasons

In absolute terms, summer *T*_max_ varied ~6°C across the elevation gradient sampled. Relative to areas with low exposure, areas with high solar radiation increased summer maximum temperatures by 5.66°C, or even up to 7.46°C for *T*_99_. This meant that summer *T*_mean_ and *T*_max_ on steep, north‐facing slopes with lower levels of potential solar radiation were up to 0.7°C and 2.9°C cooler, respectively, than highly exposed areas (Figure 4). Incised valleys were between 0.26 and 0.28°C cooler than other landforms, whereas terrain roughness decreased *T*_max_ and *T*_99_ by about 0.03 to 0.04 per index unit, up to 1.62°C. Areas fully covered by broadleaf, coniferous, or in particular mixedwood forests experienced significantly lower summer *T*_mean_ than unvegetated areas by about 0.23°C, 0.37°C, and 0.72°C, respectively (Figure 4). Over the winter, all forest cover types increased *T*_min_, particularly under mixedwood (2.01°C) and broadleaf forest cover (1.16°C). Elevation had a strong warming effect in winter *T*_min_ with an increase of up to ~5.50°C, relative to low elevation areas. Interestingly, we found that the strength of forest cover was similar to that of topographic factors, particularly for *T*_mean_, *T*_min_, and *T*_range_. Figure 4 summarizes the predicted effects for *T*_mean_ for July of 2018, which was a typical year in terms of temperature for Alberta (see Figures S5 and S6 for unstandardized coefficients of all metrics in both seasons).

We found that fixed effects and random effects together explained on average 40% more of the variance than fixed effects alone (conditional vs. marginal *R* ^2^; Table 1 and Table S1), indicating that site‐level temperature differences were strong over the large area sampled. Conditional and marginal pseudo‐*R* ^2^ values varied across seasons and temperature metrics (Table S1) but generally indicated that the top models of a given temperature metric explained over 60% and 35% of the variation, respectively. For the summer season, explanatory power attributed to variables was over 35% of the variation for *T*_max_ and *T*_range_ (*R*_m_^2^ = 0.36*–*0.39), over 34% for *T*_mean_, and over for GDD_5_ and *T*_99_ (*R*_m_^2^ = 0.27*–*0.33). For the winter season, variables explained around 35% of the variation for *T*_range_ (*R* ^2^ = 0.35*–*0.39), around 18% for *T*_mean_ (*R* ^2^ = 0.16–0.18), but did not perform well for *T*_min_ (*R* ^2^ = 0.11) or GDD_5_ (*R* ^2^ < 0.05). Appendix S2 shows diagnostic plots for all metrics and models.

### 3.2 Mapped local climate and comparison with ClimateNA

**FIGURE 5:**
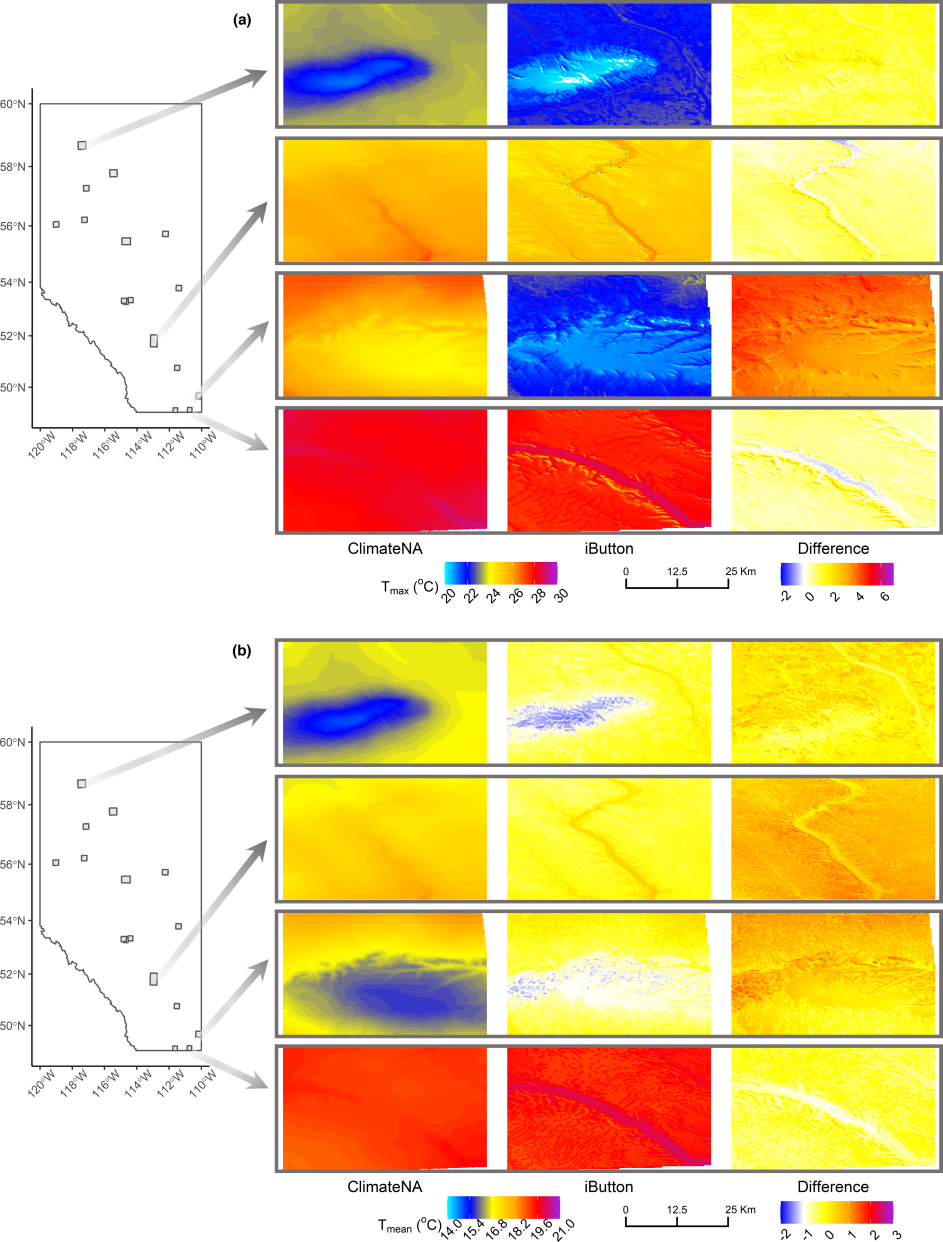
Spatial representation of the monthly average of summer daily (a) *T*_max_ and (b) *T*_mean_ for ClimateNA (first column) and iButtons (second column), and the difference between the two readings (i.e., *T*_Difference_ = *T*_ClimateNA_–*T*_iButton_; third column) over two river valley and hill systems in Alberta, Canada, in July of 2018. For differences, red/blue colors indicate higher/lower temperature predictions for ClimateNA vs iButtons, whereas white colors indicate closer predictions between the two. For clarity, we used a specific color scheme for the differences (third column) in each panel. From top to bottom rows: Watt Mountain, North Saskatchewan River, Cypress Hills, and Milk River. Greyed squares on the left map indicate the location of all study sites

We observed significant differences (i.e., *T*_Difference_ = *T*_ClimateNA_–*T*_iButton_) between ClimateNA and iButton predictions (Figure 5). At the station level, iButtons recorded, on average, warmer summer *T*_mean_, cooler summer *T*_max_, and warmer winter temperatures than ClimateNA (Figures S7 and S8). The directionality of the effect of topography and vegetation remained mostly consistent across seasons for the different metrics. Interestingly, differences in *T*_max_ and *T*_mean_ typically increased with latitude in the summer but decreased in the winter, though not always significantly.

**FIGURE 6:**
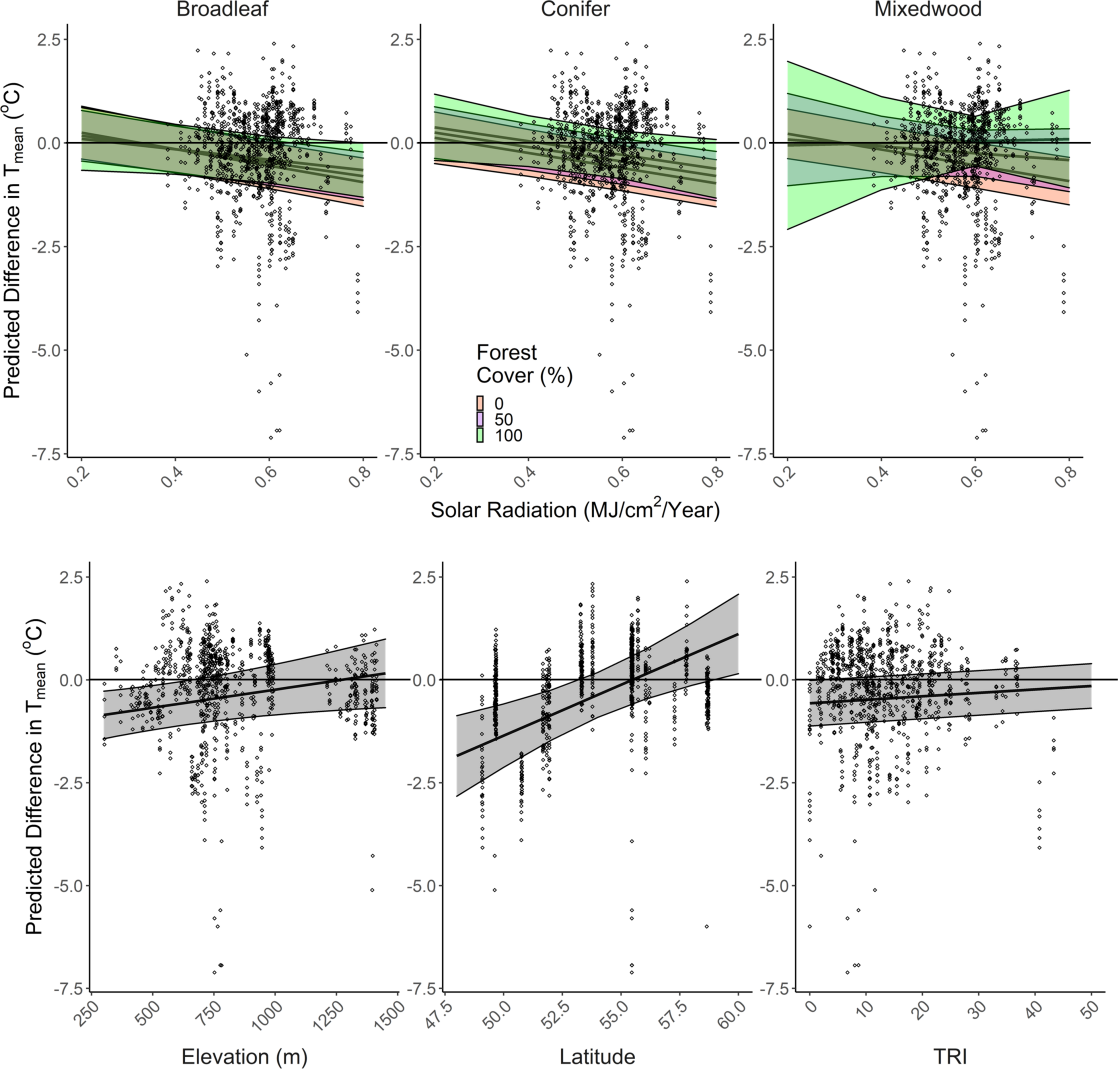
The effect of different topographic and vegetation variables on the absolute difference in summer *T*_max_ between ClimateNA and iButton readings (i.e., *T*_Difference_ = *T*_ClimateNA_–*T*_iButton_). Positive values indicate that iButton measurements were higher than ClimateNA measurements. Points represent absolute differences between the two sources at the iButton station; the regression line represented the average difference with 95% confidence intervals. The horizontal black line indicates no difference between ClimateNA and iButtons. Only significant variables at α = 0.05 are presented

During summer, the difference in *T*_max_ was ~0.22°C higher on ridge tops relative to other landforms, indicating that iButtons recorded cooler *T*_max_ on those ridges than on other landforms. The difference in *T*_max_ also increased by 0.03–1.2°C between flat and highly rugged terrain (Figure 6; Figure S7). The difference in *T*_max_ decreased with increasing solar radiation, indicating that iButton temperature predictions were almost 2°C cooler than ClimateNA in less exposed areas and 1.3°C higher in highly exposed areas when other variables were held constant at mean values (Figure S7). Differences in average temperature (*T*_mean_) between the two sources were much smaller than differences in summer maximum. We found that the difference in summer *T*_mean_ in incised valleys was 0.21°C higher than other landforms, indicating that iButtons recorded slightly cooler *T*_mean_ in these valleys than in other landforms but still warmer than ClimateNA. Differences in *T*_mean_ decreased with solar radiation and indicated that iButtons and ClimateNA predicted similar values on less exposed slopes, but iButtons recorded 0.8°C higher *T*_mean_ in highly exposed areas. We found that only coniferous forests interacted significantly with solar radiation over the summer, indicating that with low coniferous cover, iButtons recorded cooler *T*_max_ (~1°C) in poorly exposed sites and about 1.5°C warmer in highly exposed areas. The effect of solar radiation in areas with high coniferous cover was similar but not as strong, and iButtons recorded approximately 0.3°C cooler *T*_max_ in poorly exposed sites and 1°C warmer in highly exposed areas.

We found that for winter *T*_min_, iButtons predicted warmer temperatures than ClimateNA (Figure S8). The difference in *T*_min_ in incised valleys was 0.39°C higher relative to other landforms, indicating that iButtons recorded cooler *T*_min_ in incised valleys than in other landforms. ClimateNA predictions were −3.28°C to −1.90°C lower than iButtons in areas with low and high topographic wetness potential, respectively. Furthermore, we found that an interaction between broadleaf forests and solar radiation led to smaller differences in winter *T*_min_ in areas with low broadleaf forest cover and poorly exposed, where ClimateNA predicted on average 1.8°C cooler temperatures than iButtons. Differences in *T*_min_ also got smaller in areas with higher broadleaf forest cover and high exposure, and ClimateNA predicted temperatures that were 2.5°C cooler than iButtons. Across metrics, elevation had mostly no significant effects on the difference between the two sources (Figures S7 and S8), except that iButtons significantly recorded ~0.86°C warmer *T*_mean_ in low elevations and ~0.12°C cooler *T*_mean_ in high elevations (Figure 6). Differences in winter *T*_mean_ were consistent as iButtons recorded warmer temperatures with increasing elevation (up to ~3.75°C; Figure S8).

iButton predictions had a higher level of spatial heterogeneity than ClimateNA, linked to the effects of topographic variables (see Figure 5 for an illustration). Differences in summer maximum temperatures were larger in the drier and hotter hills in the southeastern part of Alberta (Cypress Hills; Figure 5, third row).

## 4 DISCUSSION

In this study of a 1000+ km span of the boreal–parkland–grassland transition zone in Western Canada, we found compelling evidence that despite gentle to moderate amounts of terrain (hill and valley systems), local topography and vegetation still have significant buffering effects on local climates. We found that not only did elevation drive differences in local climates, but roughness, aspect, landform, and vegetation also exerted significant effects. More specifically, topographically shaded sites were between 0.7 and 2.9°C cooler than flat, exposed sites in both summer and winter, whereas sites with more surrounding forest cover were up to 0.7°C cooler in summer and 2°C warmer in winter than non‐forested sites. By extension, we demonstrated that local topographic effects on temperature are not fully captured in commonly used downscaled gridded climate data products.

### 4.1 Effects of topographic factors and vegetation on local climate

#### 4.1.1 Elevation

Elevation was one of the strongest single predictors for most temperature metrics. We found that elevation decreased mean summer temperatures by almost 6°C over the 1400‐m elevation gradient that we evaluated, and increased minimum winter temperatures by up to 5.5°C, indicating winter temperature inversions. However, we found more support for models relating temperature metrics to a combination of local topography and vegetation cover. Models that accounted only for elevation received relatively little support and, in general, had lower explanatory power relative to full models. Hence, our results demonstrate that a combination of topographic factors is needed to explain local temperature variations, and therefore local heterogeneity. Landscapes with more gentle terrain, such as the prairie–parkland–boreal plain transition in Alberta, are projected to exhibit high climate change velocity compared with mountainous regions (Brito‐Morales et al., 2018; Carroll et al., 2015). However, we found that the existing topography in these transition zones creates substantial thermal heterogeneity not captured by gridded climate data products.

#### 4.1.2 Solar radiation

Not surprisingly, we found an important influence of aspect and slope, via differences in solar radiation, with highly exposed slopes being up to 2.9°C warmer than shaded areas for summer *T*_max_. However, the effect was smaller than what has been found in mountain systems (Geiger et al., 1995; Gruber et al., 2004; Huang et al., 2008; Suggitt et al., 2011). For instance, differences of 6°C in mean annual temperature between the north‐ and south‐facing slopes have been observed in steep mountainous terrain (e.g., Swiss Alps, Gruber et al., 2004), whereas smaller mountains (~300 m elevation gradients) have shown differences of 7°C in maximum temperatures and 1.3°C in mean temperatures (Wales and England, Suggitt et al., 2011). These differences are much greater than what we found and may be related to the shape of the topography we sampled, which can influence wind turbulence and the dynamics of cold and warm air. Relatedly, differences in climate conditions, steepness of elevational gradients, and instrumentation can also lead to steeper temperature gradients (Geiger et al., 1995). Perhaps, most importantly, the angle of a slope influences the solar radiation difference between aspects and probably explains much of the difference between results found here and those from studies in more rugged terrain. Finally, these differences could also be related to differences in radiation shielding (Maclean et al., 2021; Terando et al., 2017) and the efficacy of our shielding versus simpler approaches (see Suggitt et al., 2011 for more details).

#### 4.1.3 Topographic wetness

We did not find an effect of topographic wetness on maximum temperatures for the summer period, but we found evidence that it decreased winter minimum and mean temperatures. The compound topographic index (CTI) is a proxy for among other things soil water accumulation associated with topography (Gessler et al., 1995; MacMillan et al., 2000). In moisture‐limited systems, such as ours, decreased soil moisture should lead to increased summer temperatures via reduced evapotranspiration (Schwingshackl et al., 2017; Seneviratne et al., 2010). The fact that we did not observe this effect may be due to the digital elevation model (DEM) proxy used, which does not consider soil texture or other aspects of water storage capacity. Furthermore, CTI is scale‐dependent and DEM resolution influences the catchment area upstream and leads to more irregular flow pathways, which ultimately affects calculated indices (Sørensen & Seibert, 2007). The negative relationship with winter temperatures that we found suggests the potential for cold‐air pooling in low‐lying areas. However, it is important to note that CTI is a relative metric, in the sense that ravines at higher elevations can still have high CTI and likewise lower elevation areas can have low CTI.

#### 4.1.4 Terrain roughness

We found some evidence that local terrain variability may affect temperatures at local scales because the topographic roughness index (TRI) decreased summer *T*_max_, *T*_mean_, and *T*_range_. Roughness increases air motion and leads to greater vertical and horizontal mixing of air due to differential heat of slopes locally, which, in turn, can reduce temperature extremes (high and low) near the surface (Gloyne, 1967). Our results suggest that air mixing may be reducing both maximum and minimum extremes and that even the limited amounts of terrain roughness found in river valleys and boreal hills can partially buffer extremes in temperature. However, it is important to note that the effects of terrain roughness can be quite localized, particularly considering wind dynamics in complex topography (e.g., Helbig et al., 2017). Consequently, iButtons could be experiencing high degrees of variation in local temperature due to differential winds, even though we detected lower temperatures on average (Wood & Mason, 1993).

#### 4.1.5 Landform

Relative to other landforms, the temperature was generally lower in incised valleys during the summer, but we did not observe lower winter minimum temperatures in valley bottoms, relative to other landforms. We did find a positive effect of elevation on the minimum temperature in the winter, suggesting that winter temperature inversions are common in larger valleys. However, our landform categories considered a variation of neighboring cells at a small scale (300‐m radius around each pixel). Therefore, “valleys” and “ridgetops” represent small ravines and coulees that are less prone to temperature inversions. Rather they are likely associated with topographic shading, leading to negative effects on local temperatures overall.

#### 4.1.6 Vegetation cover

Our results examining the effect of vegetation cover partially supported previous studies, in that surrounding forest cover led to decreased mean temperatures in summer and increased minimum temperatures in winter (e.g., De Frenne et al., 2021). Consistent with other studies from boreal forests (as summarized by De Frenne et al., 2021), we found a stronger positive buffering effect on winter minimum temperatures compared to summer mean temperatures. This could be at least partly explained by the type of shielding we used for our iButtons, which is very effective at blocking direct solar radiation and can be slightly biased toward cooler temperatures in forested environments relative to commercially available shielding (Holden et al., 2013). Interestingly, we found that broadleaf vegetation cover had positive effects on summer temperature maxima. This could be the result of the moisture‐limited conditions that characterize the boreal–parkland–grassland transition zone (e.g., Koster et al., 2004; Seneviratne et al., 2010) because the cooling effects by evapotranspiration decrease under moisture‐limited conditions (Davis et al., 2019). Deciduous‐dominated areas may be drier than coniferous vegetation, which may reflect in warmer temperatures (Martin‐Guay et al., 2022). In addition, forest cover in the prairie and parkland portions of our study area is closely associated with north‐facing slopes, which we found to be an important negative predictor of maximum temperature (via the solar radiation metric we used), so the effect may be captured within that variable.

Canopy structure, including height and tree age, as well as moisture, has been proposed as important factors promoting the cooling of local climates (De Frenne et al., 2021; Milling et al., 2018; Renaud & Rebetez, 2009; von Arx et al., 2012). Moreover, the canopy energy balance in boreal forests displays different patterns depending on forest composition. Coniferous forest canopies have higher aerodynamic roughness relative to deciduous dominated forests, which could lead to a decrease in air temperature locally (Blanken et al., 2001; McCaughey et al., 1997). Although we did not measure local canopy cover, this agrees with our results that increasing levels of surrounding coniferous and mixedwood forest had a negative effect on summer *T*_mean_. In addition, forested plots can retain heat within the understory by blocking outgoing longwave radiation at night, thereby exhibiting higher minimum temperatures relative to treeless areas (Davis et al., 2019; De Frenne et al., 2021). Although we found that the surrounding forest cover buffered minimum annual temperatures (i.e., winter *T*_min_), we did not find a diurnal buffering of minimum temperature in summer, suggesting that other factors like topographic shading may have been overriding.

### 4.2 Mapped local climate and comparison with ClimateNA

Compared to a downscaled gridded climate product (ClimateNA), our estimates showed a similar regional pattern of air temperature but highlighted the thermal heterogeneity of the landscape. Elevation had mostly no noticeable influence on the difference between the two sources of data in summer, which suggests that they are capturing similar lapse rates and regional patterns in warm months. However, iButton data indicated a larger reduction in winter minimum and average temperatures at higher elevations, suggesting winter temperature inversions that are not captured in ClimateNA (e.g., Figures S10 and S11). Locally, the magnitude of the difference between the two estimates was greatest in areas where iButton data predicted warmer temperatures (i.e., exposed and/or non‐forested slopes), but smaller where our estimates predicted cooler temperatures (i.e., areas with high topographic shading). Since local climates are a result of the effects of local ecosystem functioning and landscape properties that modify the climate at larger scales (mesoclimate; Bailey, 2009; Chen et al., 1999; Geiger et al., 1995), gridded climate products do not capture the resulting thermal nuances in the landscape.

We also observed a substantial effect of latitude on the difference between ClimateNA and iButton measurements (generally positive in summer and negative in winter). Such differences may be related to the negative relationship between elevation and latitude in Alberta. Another possibility is the fact that we centered our solar radiation potential variable at a single latitude (see Methods section) and solar radiation decreases with latitude, which could be driving the difference with our northern sites. Of course, estimates from either source of data may have inherent biases derived from different instrumentation (weather stations vs. local climate sensors; Ashcroft, 2018). For instance, differences in *T*_mean_ between the two products were smaller than differences in *T*_min_ and *T*_max_, suggesting either that iButtons are more susceptible to temperature extremes (Maclean et al., 2021) or that ClimateNA overestimates them. iButton warm biases could be considerable in open environments if heat shields are not properly equipped (Maclean et al., 2021), although we do not believe that was an issue in our study, given the effectiveness of the Holden et al. (2013) shield design. Acknowledging these limitations, it is clear that interpolated climate products such as ClimateNA cannot fully describe the thermal heterogeneity that has implications for climate change adaptation at local and regional scales (e.g., Greenwood et al., 2016).

### 4.3 Implications for climate change adaptation

Overall, we found evidence that the combined effects of local topography and vegetation exerted a substantial influence on local temperatures. Also, their impact was comparable in magnitude to the cooling effect of elevation, even in the moderate topography of the boreal–parkland–prairie transition zone. Notably, some of the topographic and vegetation effects analyzed may promote a level of cooling that falls within near‐term climate change projections for this region. We found that areas with a low incidence of solar radiation, such as north‐facing slopes and incised ravines, may have maximum temperatures that are up to 0.7°C and 2.9°C cooler than surrounding areas during summer, whereas terrain roughness may further contribute 1.62°C. Furthermore, we found that sites with greater levels of surrounding coniferous and mixed forest cover can experience local mean summer temperatures that are nearly 1°C cooler than open sites, supporting the notion that the inherent local buffering capacity of forests might be on the same order of magnitude as expected future warming (Frey et al., 2016). Combined, the buffering capacity of topographic and vegetation effects may be comparable to the expected ~2°C increase by 2050 and ~3°C by 2100 in the boreal plains (Price et al., 2013). Boreal species that can shift to or persist in these cooler areas might be able to compensate for regional warming.

A contemporary example of this cooling effect is the presence of coniferous forest patches on north‐facing slopes in central Alberta, at the limit of drought tolerance for boreal trees. These patches are relict populations of boreal forests that once occupied the southern part of the region (e.g., Hampe & Jump, 2011). Thus, they may be analogs for how the current boreal forests in the north may be distributed in the future under climate change. In other words, the norm across northern Alberta could become patches of coniferous and mixed forest, mainly on north‐facing slopes and other sheltered sites, interspersed with grassland and open parkland. Conversely, south‐facing slopes and areas with low surrounding forest cover are more susceptible to warming and rapid climate change at the local scale. Our results suggest that the combination of forest cover and topographic setting has the potential to buffer the effects of near‐term climate change, although the level of future persistence is uncertain (De Frenne et al., 2021; De Lombaerde et al., 2022; Lembrechts & Nijs, 2020; Zellweger et al., 2020).

Our results provide empirical support for topographically and vegetation‐mediated temperature variation that may result in refugia for forest‐associated species under a warming climate (Stralberg et al., 2020). Understory‐dwelling plants and animals could benefit directly from canopy shading, whereas birds and other wildlife can benefit from forest patches retained by cooler conditions, which could be used as stepping stones as their climate niches shift northward with continued climate change. With the adoption of conservation measures and targeted forest management practices, topographically sheltered forest stands may serve as “slow‐lanes” that buffer the negative effects of climate change in the short term and provide safe havens in the long term (Morelli et al., 2020). Such practices could include the implementation of riparian buffers, strategic retention patches, and afforestation (Greenwood et al., 2016). Conservation and management strategies that target refugia and the landscape features that promote them can serve as efficient investments for short‐ and long‐term conservation goals in a changing climate.

## AUTHOR CONTRIBUTIONS

**Cesar A. Estevo:** Conceptualization (equal); Data curation (lead); Formal analysis (lead); Funding acquisition (supporting); Investigation (equal); Methodology (equal); Project administration (equal). **Diana Stralberg:** Conceptualization (equal); Data curation (equal); Formal analysis (supporting); Funding acquisition (equal); Investigation (equal); Methodology (equal); Project administration (equal); Resources (equal); Supervision (lead); Visualization (equal); Writing – review & editing (equal). **Scott E. Nielsen:** Conceptualization (equal); Funding acquisition (equal); Investigation (equal); Methodology (equal); Project administration (supporting); Resources (equal); Supervision (supporting); Visualization (equal); Writing – review & editing (equal). **Erin Bayne:** Conceptualization (equal); Funding acquisition (equal); Investigation (equal); Methodology (supporting); Project administration (supporting); Resources (equal); Supervision (lead); Visualization (equal); Writing – review & editing (equal).

## CONFLICT OF INTEREST

None declared.

## Supporting information

Appendix S1

Appendix S2

## Data Availability

Data used for this research are available through Dryad under the title “*Topographic and vegetation drivers of thermal heterogeneity along the boreal–grassland transition zone in western Canada*: *implications for climate change refugia*”, https://doi.org/10.5061/dryad.f7m0cfxw2. The data are also be available at SoilTemp (https://soiltemp.weebly.com).

## References

[ece39008-bib-0001] ABMI . (2010). Wall‐to‐wall Land Cover Map Version 2.1 (ABMIw2wLCV2010v1.0) from the Alberta Biodiversity Monitoring Institute was used, in whole or part, to create this product . http://www.abmi.ca

[ece39008-bib-0002] Ashcroft, M. B. (2018). Which is more biased: Standardized weather stations or microclimatic sensors? Ecology and Evolution, 8(11), 5231–5232. 10.1002/ece3.3965 29938045 PMC6010914

[ece39008-bib-0003] Ashcroft, M. B. , Gollan, J. R. , Warton, D. I. , & Ramp, D. (2012). A novel approach to quantify and locate potential microrefugia using topoclimate, climate stability, and isolation from the matrix. Global Change Biology, 18(6), 1866–1879. 10.1111/j.1365-2486.2012.02661.x

[ece39008-bib-0004] Bailey, R. G. (2009). Ecosystem geography. Springer New York.

[ece39008-bib-0005] Barber, Q. E. , Nielsen, S. E. , & Hamann, A. (2016). Assessing the vulnerability of rare plants using climate change velocity, habitat connectivity, and dispersal ability: A case study in Alberta, Canada. Regional Environmental Change, 16(5), 1433–1441. 10.1007/s10113-015-0870-6

[ece39008-bib-0006] Barry, R. G. (2008). Mountain weather and climate, 3rd ed. Cambridge University Press.

[ece39008-bib-0007] Barry, R. , & Blanken, P. (2016). Microclimate and local climate. Cambridge University Press.

[ece39008-bib-0008] Barton, K. (2020). MuMIn: Multi‐model inference. R Package Version, 1(43), 17. https://CRAN.R‐project.org/package=MuMIn

[ece39008-bib-0009] Blanken, P. D. , Black, T. A. , Neumann, H. H. , den Hartog, G. , Yang, P. C. , Nesic, Z. , & Lee, X. (2001). The seasonal water and energy exchange above and within a boreal aspen forest. Journal of Hydrology, 245(1–4), 118–136. 10.1016/S0022-1694(01)00343-2

[ece39008-bib-0010] Briga, M. , & Verhulst, S. (2015). Large diurnal temperature range increases bird sensitivity to climate change. Scientific Reports, 5(1), 16600. 10.1038/srep16600 26563993 PMC4643245

[ece39008-bib-0011] Brito‐Morales, I. , García Molinos, J. , Schoeman, D. S. , Burrows, M. T. , Poloczanska, E. S. , Brown, C. J. , Ferrier, S. , Harwood, T. D. , Klein, C. J. , McDonald‐Madden, E. , Moore, P. J. , Pandolfi, J. M. , Watson, J. E. M. , Wenger, A. S. , & Richardson, A. J. (2018). Climate velocity can inform conservation in a warming world. Trends in Ecology & Evolution, 33(6), 441–457. 10.1016/j.tree.2018.03.009 29716742

[ece39008-bib-0013] Burnham, K. P. , & Anderson, D. R. (Eds.) (2004). Model selection and multimodel inference. Springer New York.

[ece39008-bib-0014] Cantlon, J. E. (1953). Vegetation and microclimates on north and south slopes of Cushetunk mountain, New Jersey. Ecological Monographs, 23(3), 241–270. 10.2307/1943593

[ece39008-bib-0015] Carroll, C. , Lawler, J. J. , Roberts, D. R. , & Hamann, A. (2015). Biotic and climatic velocity identify contrasting areas of vulnerability to climate change. PLoS One, 10(10), e0140486. 10.1371/journal.pone.0140486 26466364 PMC4605713

[ece39008-bib-0016] Chen, J. , Franklin, J. F. , & Spies, T. A. (1993). Contrasting microclimates among clearcut, edge, and interior of old‐growth Douglas‐fir forest. Agricultural and Forest Meteorology, 63(3–4), 219–237. 10.1016/0168-1923(93)90061-L

[ece39008-bib-0017] Chen, J. , Saunders, S. C. , Crow, T. R. , Naiman, R. J. , Brosofske, K. D. , Mroz, G. D. , Brookshire, B. L. , & Franklin, J. F. (1999). Microclimate in forest ecosystem and landscape ecology. BioScience, 49(4), 288–297. 10.2307/1313612

[ece39008-bib-0018] Clements, C. B. , Whiteman, C. D. , & Horel, J. D. (2003). Cold‐air‐pool structure and evolution in a mountain basin: Peter Sinks, Utah. Journal of Applied Meteorology, 42, 19.

[ece39008-bib-0019] Daly, C. , Conklin, D. R. , & Unsworth, M. H. (2010). Local atmospheric decoupling in complex topography alters climate change impacts. International Journal of Climatology, 30(12), 1857–1864. 10.1002/joc.2007

[ece39008-bib-0020] Davis, K. T. , Dobrowski, S. Z. , Holden, Z. A. , Higuera, P. E. , & Abatzoglou, J. T. (2019). Microclimatic buffering in forests of the future: The role of local water balance. Ecography, 42(1), 1–11. 10.1111/ecog.03836

[ece39008-bib-0021] De Frenne, P. , Lenoir, J. , Luoto, M. , Scheffers, B. R. , Zellweger, F. , Aalto, J. , Ashcroft, M. B. , Christiansen, D. M. , Decocq, G. , De Pauw, K. , Govaert, S. , Greiser, C. , Gril, E. , Hampe, A. , Jucker, T. , Klinges, D. H. , Koelemeijer, I. A. , Lembrechts, J. J. , Marrec, R. , … Hylander, K. (2021). Forest microclimates and climate change: Importance, drivers and future research agenda. Global Change Biology, 27, 2279–2297. 10.1111/gcb.15569 33725415

[ece39008-bib-0022] De Frenne, P. , Rodríguez‐Sánchez, F. , Coomes, D. A. , Baeten, L. , Verstraeten, G. , Vellend, M. , Bernhardt‐Römermann, M. , Brown, C. D. , Brunet, J. , Cornelis, J. , Decocq, G. M. , Dierschke, H. , Eriksson, O. , Gilliam, F. S. , Hédl, R. , Heinken, T. , Hermy, M. , Hommel, P. , Jenkins, M. A. , … Verheyen, K. (2013). Microclimate moderates plant responses to macroclimate warming. Proceedings of the National Academy of Sciences of the United States of America, 110(46), 18561–18565. 10.1073/pnas.1311190110 24167287 PMC3832027

[ece39008-bib-0023] De Frenne, P. , Zellweger, F. , Rodríguez‐Sánchez, F. , Scheffers, B. R. , Hylander, K. , Luoto, M. , Vellend, M. , Verheyen, K. , & Lenoir, J. (2019). Global buffering of temperatures under forest canopies. Nature Ecology & Evolution, 3(5), 744–749. 10.1038/s41559-019-0842-1 30936433

[ece39008-bib-0024] De Lombaerde, E. , Vangansbeke, P. , Lenoir, J. , Van Meerbeek, K. , Lembrechts, J. , Rodríguez‐Sánchez, F. , Luoto, M. , Scheffers, B. , Haesen, S. , Aalto, J. , Christiansen, D. M. , De Pauw, K. , Depauw, L. , Govaert, S. , Greiser, C. , Hampe, A. , Hylander, K. , Klinges, D. , Koelemeijer, I. , … De Frenne, P. (2022). Maintaining forest cover to enhance temperature buffering under future climate change. Science of the Total Environment, 810, 151338. 10.1016/j.scitotenv.2021.151338 34748832

[ece39008-bib-0025] Dobrowski, S. Z. (2011). A climatic basis for microrefugia: The influence of terrain on climate: A climatic basis for microrefugia. Global Change Biology, 17(2), 1022–1035. 10.1111/j.1365-2486.2010.02263.x

[ece39008-bib-0026] Dyke, A. S. (2007). Late quaternary vegetation history of Northern North America based on pollen, macrofossil, and faunal remains*. Géographie Physique et Quaternaire, 59(2–3), 211–262. 10.7202/014755ar

[ece39008-bib-0027] Elsen, P. R. , Farwell, L. S. , Pidgeon, A. M. , & Radeloff, V. C. (2020). Landsat 8 TIRS‐derived relative temperature and thermal heterogeneity predict winter bird species richness patterns across the conterminous United States. Remote Sensing of Environment, 236, 111514. 10.1016/j.rse.2019.111514

[ece39008-bib-0028] Frey, S. J. K. , Hadley, A. S. , Johnson, S. L. , Schulze, M. , Jones, J. A. , & Betts, M. G. (2016). Spatial models reveal the microclimatic buffering capacity of old‐growth forests. Science Advances, 2(4), e1501392. 10.1126/sciadv.1501392 27152339 PMC4846426

[ece39008-bib-0029] Fuentes‐Hurtado, M. , Hof, A. R. , & Jansson, R. (2016). Paleodistribution modeling suggests glacial refugia in Scandinavia and out‐of‐Tibet range expansion of the Arctic fox. Ecology and Evolution, 6(1), 170–180. 10.1002/ece3.1859 26811782 PMC4716496

[ece39008-bib-0030] Fulton, R. J. (Ed.) (1989). Quaternary geology of Canada and Greenland. Geological Society of America.

[ece39008-bib-0031] Geiger, R. , Aron, R. H. , & Todhunter, P. (1995). The climate near the ground. Vieweg Teubner Verlag.

[ece39008-bib-0032] George, A. D. , Connette, G. M. , Thompson, F. R. , & Faaborg, J. (2017). Resource selection by an ectothermic predator in a dynamic thermal landscape. Ecology and Evolution, 7(22), 9557–9566. 10.1002/ece3.3440 29187989 PMC5696430

[ece39008-bib-0033] Gessler, P. E. , Moore, I. D. , McKENZIE, N. J. , & Ryan, P. J. (1995). Soil‐landscape modelling and spatial prediction of soil attributes. International Journal of Geographical Information Systems, 9(4), 421–432. 10.1080/02693799508902047

[ece39008-bib-0034] Gloyne, R. W. (1967). Wind as a factor in hill climates. In J. A. Taylor (Ed.), Weather and agriculture (pp. 59–67). Elsevier.

[ece39008-bib-0035] Greenwood, O. , Mossman, H. L. , Suggitt, A. J. , Curtis, R. J. , & Maclean, I. M. D (2016). Using in situ management to conserve biodiversity under climate change. Journal of Applied Ecology, 53(3), 885–894. 10.1111/1365-2664.12602 27609987 PMC4991270

[ece39008-bib-0036] Gruber, S. , Hoelzle, M. , & Haeberli, W. (2004). Rock‐wall temperatures in the Alps: Modelling their topographic distribution and regional differences. Permafrost and Periglacial Processes, 15(3), 299–307. 10.1002/ppp.501

[ece39008-bib-0038] Hampe, A. , & Jump, A. S. (2011). Climate relicts: Past, present, future. Annual Review of Ecology, Evolution, and Systematics, 42(1), 313–333. 10.1146/annurev-ecolsys-102710-145015

[ece39008-bib-0039] Hannah, L. , Flint, L. , Syphard, A. D. , Moritz, M. A. , Buckley, L. B. , & McCullough, I. M. (2014). Fine‐grain modeling of species’ response to climate change: Holdouts, stepping‐stones, and microrefugia. Trends in Ecology & Evolution, 29(7), 390–397. 10.1016/j.tree.2014.04.006 24875589

[ece39008-bib-0040] Helbig, N. , Mott, R. , van Herwijnen, A. , Winstral, A. , & Jonas, T. (2017). Parameterizing surface wind speed over complex topography. Journal of Geophysical Research: Atmospheres, 122(2), 651–667. 10.1002/2016JD025593

[ece39008-bib-0041] Hogg, E. H. (1994). Climate and the southern limit of the western Canadian boreal forest’. Canadian Journal of Forest Research, 24(9), 1835–1845. 10.1139/x94-237

[ece39008-bib-0042] Holden, Z. A. , Klene, A. E. , F. Keefe, R. , & G. Moisen, G. (2013). Design and evaluation of an inexpensive radiation shield for monitoring surface air temperatures. Agricultural and Forest Meteorology, 180, 281–286. 10.1016/j.agrformet.2013.06.011

[ece39008-bib-0043] Høyvik Hilde, C. , Pélabon, C. , Guéry, L. , Gabrielsen, G. W. , & Descamps, S. (2016). Mind the wind: Microclimate effects on incubation effort of an arctic seabird. Ecology and Evolution, 6(7), 1914–1921. 10.1002/ece3.1988 27099703 PMC4831427

[ece39008-bib-0044] Huang, S. , Rich, P. M. , Crabtree, R. L. , Potter, C. S. , & Fu, P. (2008). Modeling monthly near‐surface air temperature from solar radiation and lapse rate: Application over complex terrain in Yellowstone National Park. Physical Geography, 29(2), 158–178. 10.2747/0272-3646.29.2.158

[ece39008-bib-0045] Jenness, J. (2006). Topographic Position Index (tpi_jen.avx) extension for ArcView 3.x, v. 1.3a., Jenness Enterprises . http://www.jennessent.com/arcview/tpi.htm

[ece39008-bib-0046] Keppel, G. , Robinson, T. P. , Wardell‐Johnson, G. W. , Yates, C. J. , Van Niel, K. P. , Byrne, M. , & Schut, A. G. T. (2017). A low‐altitude mountain range as an important refugium for two narrow endemics in the Southwest Australian Floristic Region biodiversity hotspot. Annals of Botany, 119(2), 289–300. 10.1093/aob/mcw182 27634576 PMC5321060

[ece39008-bib-0047] Keppel, G. , Van Niel, K. P. , Wardell‐Johnson, G. W. , Yates, C. J. , Byrne, M. , Mucina, L. , Schut, A. G. T. , Hopper, S. D. , & Franklin, S. E. (2012). Refugia: Identifying and understanding safe havens for biodiversity under climate change: Identifying and understanding refugia. Global Ecology and Biogeography, 21(4), 393–404. 10.1111/j.1466-8238.2011.00686.x

[ece39008-bib-0048] Koster, R. D. , Dirmeyer, P. A. , Guo, Z. , Bonan, G. , Chan, E. , Cox, P. , Gordon, C. T. , Kanae, S. , Kowalczyk, E. , Lawrence, D. , Liu, P. , Lu, C.‐H. , Malyshev, S. , McAvaney, B. , Mitchell, K. , Mocko, D. , Oki, T. , Oleson, K. , Pitman, A. , … Yamada, T. (2004). Regions of strong coupling between soil moisture and precipitation. Science, 305(5687), 1138–1140. 10.1126/science.1100217 15326351

[ece39008-bib-0049] Lampei, C. , Wunder, J. , Wilhalm, T. , & Schmid, K. J. (2019). Microclimate predicts frost hardiness of alpine *Arabidopsis thaliana* populations better than elevation. Ecology and Evolution, 9(23), 13017–13029. 10.1002/ece3.5659 31871626 PMC6912909

[ece39008-bib-0050] Leipold, M. , Tausch, S. , Poschlod, P. , & Reisch, C. (2017). Species distribution modeling and molecular markers suggest longitudinal range shifts and cryptic northern refugia of the typical calcareous grassland species *Hippocrepis comosa* (horseshoe vetch). Ecology and Evolution, 7(6), 1919–1935. 10.1002/ece3.2811 28331599 PMC5355195

[ece39008-bib-0052] Lembrechts, J. J. , & Nijs, I. (2020). Microclimate shifts in a dynamic world. Science, 368(6492), 711–712. 10.1126/science.abc1245 32409462

[ece39008-bib-0104] Lenoir, J. , Hattab, T. , & Pierre, G. (2016). Climatic microrefugia under anthropogenic climate change: Implications for species redistribution. Ecography, 40(2), 253–266. 10.1111/ecog.02788

[ece39008-bib-0054] Letten, A. D. , Ashcroft, M. B. , Keith, D. A. , Gollan, J. R. , & Ramp, D. (2013). The importance of temporal climate variability for spatial patterns in plant diversity. Ecography, 36(12), 1341–1349. 10.1111/j.1600-0587.2013.00346.x

[ece39008-bib-0055] Loarie, S. R. , Duffy, P. B. , Hamilton, H. , Asner, G. P. , Field, C. B. , & Ackerly, D. D. (2009). The velocity of climate change. Nature, 462(7276), 1052–1055. 10.1038/nature08649 20033047

[ece39008-bib-0056] Lookingbill, T. R. , & Urban, D. L. (2003). Spatial estimation of air temperature differences for landscape‐scale studies in montane environments. Agricultural and Forest Meteorology, 114(3), 141–151. 10.1016/S0168-1923(02)00196-X

[ece39008-bib-0057] Maclean, I. M. D. , Duffy, J. P. , Haesen, S. , Govaert, S. , De Frenne, P. , Vanneste, T. , Lenoir, J. , Lembrechts, J. J. , Rhodes, M. W. , & Van Meerbeek, K. (2021). On the measurement of microclimate. Methods in Ecology and Evolution, 12(8), 1397–1410. 10.1111/2041-210X.13627

[ece39008-bib-0058] MacMillan, R. A. , Pettapiece, W. W. , Nolan, S. C. , & Goddard, T. W. (2000). A generic procedure for automatically segmenting landforms into landform elements using DEMs, heuristic rules and fuzzy logic. Fuzzy Sets and Systems, 113(1), 81–109. 10.1016/S0165-0114(99)00014-7

[ece39008-bib-0059] Martin‐Guay, M.‐O. , Belluau, M. , Côté, B. , Handa, I. T. , Jewell, M. D. , Khlifa, R. , Munson, A. D. , Rivest, M. , Whalen, J. K. , & Rivest, D. (2022). Tree identity and diversity directly affect soil moisture and temperature but not soil carbon ten years after planting. Ecology and Evolution, 12(1), e8509. 10.1002/ece3.8509 35136558 PMC8809433

[ece39008-bib-0060] McCaughey, J. H. , Amiro, B. D. , Robertson, A. W. , & Spittlehouse, D. L. (1997). Forest environments. In W. G. Bailey , T. R. Oke , & W. R. Rouse (Eds.), The surface climates of Canada (pp. 247–276). McGill‐Queen’s University Press.

[ece39008-bib-0061] McCune, B. (2007). Improved estimates of incident radiation and heat load using non‐parametric regression against topographic variables. Journal of Vegetation Science, 18(5), 751–754. 10.1111/j.1654-1103.2007.tb02590.x

[ece39008-bib-0062] McCune, B. , & Keon, D. (2002). Equations for potential annual direct incident radiation and heat load. Journal of Vegetation Science, 13(4), 603–606. 10.1111/j.1654-1103.2002.tb02087.x

[ece39008-bib-0063] Milling, C. R. , Rachlow, J. L. , Olsoy, P. J. , Chappell, M. A. , Johnson, T. R. , Forbey, J. S. , Shipley, L. A. , & Thornton, D. H. (2018). Habitat structure modifies microclimate: An approach for mapping fine‐scale thermal refuge. Methods in Ecology and Evolution, 9(6), 1648–1657. 10.1111/2041-210X.13008

[ece39008-bib-0064] Morelli, T. L. , Barrows, C. W. , Ramirez, A. R. , Cartwright, J. M. , Ackerly, D. D. , Eaves, T. D. , Ebersole, J. L. , Krawchuk, M. A. , Letcher, B. H. , Mahalovich, M. F. , Meigs, G. W. , Michalak, J. L. , Millar, C. I. , Quiñones, R. M. , Stralberg, D. , & Thorne, J. H. (2020). Climate‐change refugia: Biodiversity in the slow lane. Frontiers in Ecology and the Environment, 18(5), 228–234. 10.1002/fee.2189 33424494 PMC7787983

[ece39008-bib-0065] Morelli, T. L. , Daly, C. , Dobrowski, S. Z. , Dulen, D. M. , Ebersole, J. L. , Jackson, S. T. , Lundquist, J. D. , Millar, C. I. , Maher, S. P. , Monahan, W. B. , Nydick, K. R. , Redmond, K. T. , Sawyer, S. C. , Stock, S. , & Beissinger, S. R. (2016). Managing climate change refugia for climate adaptation. PLoS One, 11(8), e0159909. 10.1371/journal.pone.0159909 27509088 PMC4980047

[ece39008-bib-0066] Moritz, S. , & Bartz‐Beielstein, T. (2017). imputeTS: Time series missing value imputation in R. The R Journal, 9(1), 207–218. 10.32614/RJ-2017-009

[ece39008-bib-0067] Moss, E. H. (1955). The vegetation of Alberta. The Botanical Review, 21(9), 493–567. 10.1007/BF02872442

[ece39008-bib-0068] Nakagawa, S. , & Schielzeth, H. (2013). A general and simple method for obtaining *R* ^2^ from generalized linear mixed‐effects models. Methods in Ecology and Evolution, 4(2), 133–142. 10.1111/j.2041-210x.2012.00261.x

[ece39008-bib-0069] Nevo, E. (2012). “Evolution Canyon”, a potential microscale monitor of global warming across life. Proceedings of the National Academy of Sciences of the United States of America, 109(8), 2960–2965. 10.1073/pnas.1120633109 22308456 PMC3286920

[ece39008-bib-0070] Nielsen, S. E. , Boyce, M. S. , & Stenhouse, G. B. (2004). Grizzly bears and forestry: I. Selection of clearcuts by grizzly bears in west‐central Alberta, Canada. Forest Ecology and Management, 199(1), 51–65. 10.1016/j.foreco.2004.04.014

[ece39008-bib-0071] Nielsen, S. E. , & Haney, A. (1998). Gradient responses for understory species in a Bracken grassland and northern dry forest ecosystem of northeast Wisconsin. Transactions of the Wisconsin Academy of Sciences Arts and Letters, 86, 149.

[ece39008-bib-0072] Norris, C. , Hobson, P. , & Ibisch, P. L. (2012). Microclimate and vegetation function as indicators of forest thermodynamic efficiency. Journal of Applied Ecology, 49(3), 562–570. 10.1111/j.1365-2664.2011.02084.x

[ece39008-bib-0073] Pinheiro, J. , Bates, D. , DebRoy, S. , Sarkar, D. , & R Core Team . (2007). Linear and nonlinear mixed effects models. R Package Version, 3(57), 1–89.

[ece39008-bib-0074] Price, D. T. , Alfaro, R. I. , Brown, K. J. , Flannigan, M. D. , Fleming, R. A. , Hogg, E. H. , Girardin, M. P. , Lakusta, T. , Johnston, M. , McKenney, D. W. , Pedlar, J. H. , Stratton, T. , Sturrock, R. N. , Thompson, I. D. , Trofymow, J. A. , & Venier, L. A. (2013). Anticipating the consequences of climate change for Canada’s boreal forest ecosystems. Environmental Reviews, 21(4), 322–365. 10.1139/er-2013-0042

[ece39008-bib-0075] R Core Team . (2013). R: A language and environment for statistical computing. R Foundation for Statistical Computing.

[ece39008-bib-0076] Rehfeldt, G. E. , Crookston, N. L. , Sáenz‐Romero, C. , & Campbell, E. M. (2012). North American vegetation model for land‐use planning in a changing climate: A solution to large classification problems. Ecological Applications, 22(1), 119–141. 10.1890/11-0495.1 22471079

[ece39008-bib-0077] Renaud, V. , & Rebetez, M. (2009). Comparison between open‐site and below‐canopy climatic conditions in Switzerland during the exceptionally hot summer of 2003. Agricultural and Forest Meteorology, 149(5), 873–880. 10.1016/j.agrformet.2008.11.006

[ece39008-bib-0078] Rho, P. (2002). Wetness: An Avenue Script for ArcView 3.2 . http://www.arcscripts.esri.com/details.asp

[ece39008-bib-0079] Riley, S. , Degloria, S. , & Elliot, S. D. (1999). A terrain ruggedness index that quantifies topographic heterogeneity. International Journal of Science, 5, 23–27.

[ece39008-bib-0080] Scheffers, B. R. , Edwards, D. P. , Diesmos, A. , Williams, S. E. , & Evans, T. A. (2014). Microhabitats reduce animal’s exposure to climate extremes. Global Change Biology, 20, 495–503. 10.1111/gcb.12439 24132984

[ece39008-bib-0081] Schneider, R. R. (2013). Alberta’s natural subregions under a changing climate: Past, present and future. University of Alberta Libraries.

[ece39008-bib-0082] Schooler, S. L. , Johnson, M. D. , Njoroge, P. , & Bean, W. T. (2020). Shade trees preserve avian insectivore biodiversity on coffee farms in a warming climate. Ecology and Evolution, 10(23), 12960–12972. 10.1002/ece3.6879 33304508 PMC7713971

[ece39008-bib-0083] Schwingshackl, C. , Hirschi, M. , & Seneviratne, S. I. (2017). Quantifying spatiotemporal variations of soil moisture control on surface energy balance and near‐surface air temperature. Journal of Climate, 30(18), 7105–7124. 10.1175/JCLI-D-16-0727.1

[ece39008-bib-0084] Sears, M. W. , Raskin, E. , & Angilletta, M. J. (2011). The world is not flat: Defining relevant thermal landscapes in the context of climate change. Integrative and Comparative Biology, 51(5), 666–675. 10.1093/icb/icr111 21937668

[ece39008-bib-0085] Seneviratne, S. I. , Corti, T. , Davin, E. L. , Hirschi, M. , Jaeger, E. B. , Lehner, I. , Orlowsky, B. , & Teuling, A. J. (2010). Investigating soil moisture–climate interactions in a changing climate: A review. Earth‐Science Reviews, 99(3–4), 125–161. 10.1016/j.earscirev.2010.02.004

[ece39008-bib-0086] Sørensen, R. , & Seibert, J. (2007). Effects of DEM resolution on the calculation of topographical indices: TWI and its components. Journal of Hydrology, 347(1–2), 79–89. 10.1016/j.jhydrol.2007.09.001

[ece39008-bib-0087] Stralberg, D. , Arseneault, D. , Baltzer, J. L. , Barber, Q. E. , Bayne, E. M. , Boulanger, Y. , Brown, C. D. , Cooke, H. A. , Devito, K. , Edwards, J. , Estevo, C. A. , Flynn, N. , Frelich, L. E. , Hogg, E. H. , Johnston, M. , Logan, T. , Matsuoka, S. M. , Moore, P. , Morelli, T. L. , … Whitman, E. (2020). Climate‐change refugia in boreal North America: What, where, and for how long? Frontiers in Ecology and the Environment, 18(5), 261–270. 10.1002/fee.2188

[ece39008-bib-0088] Stralberg, D. , Matsuoka, S. M. , Hamann, A. , Bayne, E. M. , Sólymos, P. , Schmiegelow, F. K. A. , Wang, X. , Cumming, S. G. , & Song, S. J. (2015). Projecting boreal bird responses to climate change: The signal exceeds the noise. Ecological Applications, 25(1), 52–69. 10.1890/13-2289.1 26255357

[ece39008-bib-0089] Strong, W. L. , & Hills, L. V. (2005). Late‐glacial and Holocene palaeovegetation zonal reconstruction for central and north‐central North America: Late‐glacial and Holocene palaeovegetation zonal reconstruction. Journal of Biogeography, 32(6), 1043–1062. 10.1111/j.1365-2699.2004.01223.x

[ece39008-bib-0090] Suggitt, A. J. , Gillingham, P. K. , Hill, J. K. , Huntley, B. , Kunin, W. E. , Roy, D. B. , & Thomas, C. D. (2011). Habitat microclimates drive fine‐scale variation in extreme temperatures. Oikos, 120(1), 1–8. 10.1111/j.1600-0706.2010.18270.x

[ece39008-bib-0091] Suggitt, A. J. , Wilson, R. J. , Isaac, N. J. B. , Beale, C. M. , Auffret, A. G. , August, T. , Bennie, J. J. , Crick, H. Q. P. , Duffield, S. , Fox, R. , Hopkins, J. J. , Macgregor, N. A. , Morecroft, M. D. , Walker, K. J. , & Maclean, I. M. D. (2018). Extinction risk from climate change is reduced by microclimatic buffering. Nature Climate Change, 8(8), 713–717. 10.1038/s41558-018-0231-9

[ece39008-bib-0092] Swanson, F. J. , Kratz, T. K. , Caine, N. , & Woodmansee, R. G. (1988). Landform effects on ecosystem patterns and processes. BioScience, 38(2), 92–98. 10.2307/1310614

[ece39008-bib-0093] Terando, A. J. , Youngsteadt, E. , Meineke, E. K. , & Prado, S. G. (2017). Ad hoc instrumentation methods in ecological studies produce highly biased temperature measurements. Ecology and Evolution, 7(23), 9890–9904. 10.1002/ece3.3499 29238523 PMC5723608

[ece39008-bib-0094] Thornthwaite, C. W. (1954). Topoclimatology. In Proceedings of the Toronto meteorological conference, 9–15 Sept 1953 (pp. 227–232). Royal Meterological Society.

[ece39008-bib-0095] Vanwalleghem, T. , & Meentemeyer, R. K. (2009). Predicting forest microclimate in heterogeneous landscapes. Ecosystems, 12(7), 1158–1172. 10.1007/s10021-009-9281-1

[ece39008-bib-0096] von Arx, G. , Dobbertin, M. , & Rebetez, M. (2012). Spatio‐temporal effects of forest canopy on understory microclimate in a long‐term experiment in Switzerland. Agricultural and Forest Meteorology, 166–167, 144–155. 10.1016/j.agrformet.2012.07.018

[ece39008-bib-0097] Wang, T. , Hamann, A. , Spittlehouse, D. , & Carroll, C. (2016). Locally downscaled and spatially customizable climate data for historical and future periods for North America. PLoS One, 11(6), e0156720. 10.1371/journal.pone.0156720 27275583 PMC4898765

[ece39008-bib-0098] Williams, R. , & Thorp, T. (2015). Characteristics of springtime nocturnal temperature inversions in a high latitude environment. Weather, 70, S37–S43. 10.1002/wea.2554

[ece39008-bib-0099] Wolff, C. L. , Demarais, S. , Brooks, C. P. , & Barton, B. T. (2020). Behavioral plasticity mitigates the effect of warming on white‐tailed deer. Ecology and Evolution, 10(5), 2579–2587. 10.1002/ece3.6087 32185003 PMC7069326

[ece39008-bib-0101] Wood, N. , & Mason, P. (1993). The Pressure force induced by neutral, turbulent flow over hills. Quarterly Journal of the Royal Meteorological Society, 119(514), 1233–1267. 10.1002/qj.49711951402

[ece39008-bib-0102] Zellweger, F. , De Frenne, P. , Lenoir, J. , Vangansbeke, P. , Verheyen, K. , Bernhardt‐Römermann, M. , Baeten, L. , Hédl, R. , Berki, I. , Brunet, J. , Van Calster, H. , Chudomelová, M. , Decocq, G. , Dirnböck, T. , Durak, T. , Heinken, T. , Jaroszewicz, B. , Kopecký, M. , Máliš, F. , … Coomes, D. (2020). Forest microclimate dynamics drive plant responses to warming. Science, 368(6492), 72–775. 10.1126/science.aba6880 32409476

