# Supplementary material for: Topographic and vegetation drivers of thermal heterogeneity along the boreal–grassland transition zone in western Canada: Implications for climate change refugia
Source: Ecol Evol. 2022 Jun 22;12(6):e9008. doi: 10.1002/ece3.9008 (PMC9217894; doi:10.1002/ece3.9008)
Supplement: Supplementary file 1 — Appendix S1 [file ECE3-12-e9008-s002.docx]

**Topographic and vegetation drivers of thermal refugia along the boreal–grassland transition zone in western Canada**

Estevo, C. A.^1^; Stralberg, D.^2^; Nielsen, S.E.^3^; Bayne, E.^1^

^1^ Department of Biological Sciences, University of Alberta, Edmonton, Canada

^2^ Natural Resources Canada, Northern Forestry Centre, Edmonton, Alberta, Canada

^3^ Department of Renewable Resources, University of Alberta, Edmonton, Canada

# A[ppendix S1](#other_s1)

[**TABLE S1**](#aptab_modelranking)**: Model ranking and standardized coefficients for all temperature metrics for the summer and winter months between 2014 and 2020 in river valley and hill systems in Alberta, Canada. Only the top three models are presented. Please refer to** [**Table 2**](#tab_modelscompared) **for variables in each model. K - number of parameters, ω - weighted AICc of the model, LL - negative log-likelihood. * denote significant coefficients at ɑ=0.05.**

|  |  |  |  |  |  |  |  |  | **Standardized β Coefficient** | | | | | | | | | | |
| --- | --- | --- | --- | --- | --- | --- | --- | --- | --- | --- | --- | --- | --- | --- | --- | --- | --- | --- | --- |
| **Season** | **Metric** | **Model Name** | **K** | **ΔAICc** | **ω** | **LL** | **R2m** | **R2c** | **Intercept** | **Elevation** | **Lat** | **Solar Radiation** | **Incised Valleys** | **Ridge Top** | **Ter. Roughness Index** | **Comp. Topo. Index** | **Broadleaf** | **Conifer** | **Mixedwood** |
| **Summer** | **T_max_** | Topodiversity and Vegetation | 17 | 0.00 | 0.55 | -2451.64 | 0.39 | 0.84 | 22.68 * | -1.41 * | -3.38 * | 0.53 * | -0.3 * | -0.28 * | -0.22 * | - | 0.24 * | 0.15 | 0.02 |
|  |  | Full | 18 | 1.42 | 0.27 | -2451.32 | 0.39 | 0.84 | 22.67 * | -1.42 * | -3.37 * | 0.53 * | -0.26 * | -0.29 * | -0.24 * | -0.04 | 0.24 * | 0.15 | 0.01 |
|  |  | Topodiversity | 14 | 2.28 | 0.18 | -2455.86 | 0.39 | 0.84 | 22.6 * | -1.48 * | -3.22 * | 0.52 * | -0.34 * | -0.25 * | -0.21 * | - | - | - | - |
|  |  | Aspect | 11 | 20.94 | 0.00 | -2468.25 | 0.40 | 0.83 | 22.38 * | -1.47 * | -3.21 * | 0.5 * | - | - | - | - | - | - | - |
|  |  | Elevation | 10 | 123.32 | 0.00 | -2520.46 | 0.42 | 0.82 | 22.35 * | -1.53 * | -3.41 * | - | - | - | - | - | - | - | - |
|  |  | Moisture and Landform | 13 | 123.58 | 0.00 | -2517.53 | 0.42 | 0.83 | 22.51 * | -1.48 * | -3.4 * | - | -0.23 | -0.24 * | - | 0.02 | - | - | - |
|  |  | Null | 9 | 172.42 | 0.00 | -2546.03 | 0.37 | 0.83 | 22.99 * | - | -2.77 * | - | - | - | - | - | - | - | - |
|  | **T_99_** | Topodiversity | 14 | 0.00 | 0.54 | -3189.71 | 0.33 | 0.74 | 31.2 * | -1.49 * | -3.95 * | 0.66 * | -0.5 * | -0.54 * | -0.28 * | - | - | - | - |
|  |  | Topodiversity and Vegetation | 17 | 1.73 | 0.23 | -3187.50 | 0.33 | 0.75 | 31.26 * | -1.64 * | -4.21 * | 0.7 * | -0.48 * | -0.54 * | -0.3 * | - | 0.23 | 0.33 | 0.03 |
|  |  | Full | 18 | 1.77 | 0.22 | -3186.49 | 0.33 | 0.74 | 31.23 * | -1.66 * | -4.19 * | 0.7 * | -0.34 | -0.55 * | -0.33 * | -0.14 | 0.22 | 0.33 | 0.02 |
|  |  | Aspect | 11 | 11.05 | 0.00 | -3198.30 | 0.34 | 0.73 | 30.77 * | -1.65 * | -4.02 * | 0.64 * | - | - | - | - | - | - | - |
|  |  | Moisture and Landform | 13 | 59.47 | 0.00 | -3220.47 | 0.36 | 0.73 | 31.07 * | -1.49 * | -4.16 * | - | -0.26 | -0.54 * | - | -0.05 | - | - | - |
|  |  | Elevation | 10 | 60.57 | 0.00 | -3224.08 | 0.36 | 0.73 | 30.74 * | -1.71 * | -4.27 * | - | - | - | - | - | - | - | - |
|  |  | Null | 9 | 80.82 | 0.00 | -3235.22 | 0.32 | 0.74 | 31.44 * | - | -3.48 * | - | - | - | - | - | - | - | - |
|  | **T_min_** | Topodiversity and Vegetation | 17 | 0.00 | 0.63 | -1784.97 | 0.09 | 0.84 | 10.04 * | -0.49 * | -0.11 | -0.04 | -0.27 * | 0.41 * | 0.13 * | - | -0.18 * | -0.23 * | -0.19 * |
|  |  | Full | 18 | 1.03 | 0.37 | -1784.45 | 0.09 | 0.84 | 10.03 * | -0.5 * | -0.11 | -0.04 | -0.24 * | 0.41 * | 0.12 * | -0.03 | -0.18 * | -0.23 * | -0.19 * |
|  |  | Topodiversity | 14 | 27.93 | 0.00 | -1802.01 | 0.04 | 0.82 | 10.15 * | -0.38 * | -0.2 | -0.01 | -0.2 * | 0.4 * | 0.11 * | - | - | - | - |
|  |  | Moisture and Landform | 13 | 38.00 | 0.00 | -1808.07 | 0.04 | 0.81 | 10.17 * | -0.41 * | -0.2 | - | -0.2 * | 0.4 * | - | -0.05 | - | - | - |
|  |  | Elevation | 10 | 100.92 | 0.00 | -1842.58 | 0.01 | 0.78 | 10.45 * | 0.19 | 0.11 | - | - | - | - | - | - | - | - |
|  |  | Null | 9 | 101.49 | 0.00 | -1843.89 | 0.00 | 0.78 | 10.38 * | - | 0.01 | - | - | - | - | - | - | - | - |
|  |  | Aspect | 11 | 102.95 | 0.00 | -1842.58 | 0.01 | 0.78 | 10.45 * | 0.19 | 0.11 | 0 | - | - | - | - | - | - | - |
| **Summer** | **T_mean_** | Topodiversity and Vegetation | 17 | 0.00 | 0.73 | -1469.74 | 0.37 | 0.95 | 15.9 * | -1.13 * | -1.79 * | 0.12 * | -0.27 * | 0.11 * | -0.05 * | - | -0.1 * | -0.16 * | -0.13 * |
|  |  | Full | 18 | 1.99 | 0.27 | -1469.71 | 0.37 | 0.95 | 15.9 * | -1.13 * | -1.79 * | 0.12 * | -0.28 * | 0.11 * | -0.05 * | 0.01 | -0.1 * | -0.16 * | -0.13 * |
|  |  | Topodiversity | 14 | 24.29 | 0.00 | -1484.96 | 0.34 | 0.95 | 15.97 * | -1.08 * | -1.85 * | 0.14 * | -0.23 * | 0.11 * | -0.06 * | - | - | - | - |
|  |  | Aspect | 11 | 51.05 | 0.00 | -1501.41 | 0.32 | 0.94 | 16.04 * | -0.78 * | -1.73 * | 0.14 * | - | - | - | - | - | - | - |
|  |  | Moisture and Landform | 13 | 74.32 | 0.00 | -1511.00 | 0.36 | 0.95 | 15.96 * | -1.08 * | -1.95 * | - | -0.22 * | 0.11 * | - | 0.03 | - | - | - |
|  |  | Elevation | 10 | 92.73 | 0.00 | -1523.27 | 0.33 | 0.94 | 16.04 * | -0.79 * | -1.81 * | - | - | - | - | - | - | - | - |
|  |  | Null | 9 | 162.77 | 0.00 | -1559.30 | 0.30 | 0.93 | 16.37 * | - | -1.44 * | - | - | - | - | - | - | - | - |
|  | **GDD_5_** | Topodiversity and Vegetation | 17 | 0.00 | 0.62 | -7406.31 | 0.29 | 0.84 | 422.66 * | -56.84 * | -134.06 * | -2.4 | -3.72 | 2.26 | -6.62 * | - | -10.42 * | -9.87 * | -9.91 * |
|  |  | Full | 18 | 1.39 | 0.31 | -7405.97 | 0.29 | 0.84 | 423.22 * | -56.45 * | -134.21 * | -2.44 | -5.85 | 2.41 | -6.01 * | 2.23 | -10.18 * | -9.79 * | -9.79 * |
|  |  | Topodiversity | 14 | 5.26 | 0.04 | -7412.02 | 0.27 | 0.84 | 429.25 * | -47.18 * | -136.59 * | -1.17 | 0.17 | 0.79 | -7.82 * | - | - | - | - |
|  |  | Elevation | 10 | 8.23 | 0.01 | -7417.58 | 0.27 | 0.84 | 427.45 * | -48.86 * | -138.43 * | - | - | - | - | - | - | - | - |
|  |  | Aspect | 11 | 9.66 | 0.00 | -7417.28 | 0.28 | 0.84 | 427.43 * | -49.04 * | -139.63 * | -1.84 | - | - | - | - | - | - | - |
|  |  | Moisture and Landform | 13 | 10.27 | 0.00 | -7415.54 | 0.27 | 0.84 | 429.27 * | -44.66 * | -136.45 * | - | -1.62 | 0.31 | - | 4.93 | - | - | - |
|  |  | Null | 9 | 25.87 | 0.00 | -7427.42 | 0.25 | 0.85 | 447.22 * | - | -114.49 * | - | - | - | - | - | - | - | - |
|  | **T_range_** | Topodiversity and Vegetation | 17 | 0.00 | 0.71 | -2668.83 | 0.36 | 0.73 | 12.56 * | -1.02 * | -3.02 * | 0.56 * | -0.13 | -0.68 * | -0.35 * | - | 0.34 * | 0.34 * | 0.19 * |
|  |  | Full | 18 | 2.06 | 0.25 | -2668.83 | 0.36 | 0.73 | 12.56 * | -1.02 * | -3.02 * | 0.56 * | -0.13 | -0.68 * | -0.35 * | 0 | 0.34 * | 0.34 * | 0.19 * |
|  |  | Topodiversity | 14 | 5.64 | 0.04 | -2674.72 | 0.38 | 0.70 | 12.38 * | -1.17 * | -2.79 * | 0.52 * | -0.22 | -0.64 * | -0.31 * | - | - | - | - |
|  |  | Aspect | 11 | 49.24 | 0.00 | -2699.59 | 0.42 | 0.68 | 11.85 * | -1.67 * | -3.02 * | 0.49 * | - | - | - | - | - | - | - |
|  |  | Moisture and Landform | 13 | 98.50 | 0.00 | -2722.18 | 0.40 | 0.68 | 12.27 * | -1.16 * | -2.94 * | - | -0.13 | -0.64 * | - | 0.08 | - | - | - |
|  |  | Elevation | 10 | 114.17 | 0.00 | -2733.07 | 0.43 | 0.67 | 11.82 * | -1.73 * | -3.2 * | - | - | - | - | - | - | - | - |
|  |  | Null | 9 | 157.92 | 0.00 | -2755.96 | 0.34 | 0.69 | 12.54 * | - | -2.44 * | - | - | - | - | - | - | - | - |
| **Winter** | **T_max_** | Topodiversity and Vegetation | 17 | 0.00 | 0.40 | -1839.46 | 0.33 | 0.93 | -4.97 * | 0.22 | -2.87 * | 0.34 * | -0.28 * | 0.01 | 0.06 | - | 0.04 | -0.08 | 0.06 |
|  |  | Full | 18 | 0.03 | 0.40 | -1838.44 | 0.33 | 0.93 | -4.98 * | 0.22 | -2.87 * | 0.34 * | -0.23 * | 0.01 | 0.05 | -0.06 | 0.04 | -0.07 | 0.06 |
|  |  | Topodiversity | 14 | 1.37 | 0.20 | -1843.24 | 0.32 | 0.93 | -5.01 * | 0.04 | -2.95 * | 0.35 * | -0.31 * | 0.02 | 0.06 | - | - | - | - |
|  |  | Aspect | 11 | 15.75 | 0.00 | -1853.50 | 0.33 | 0.93 | -4.96 * | 0.35 * | -2.78 * | 0.35 * | - | - | - | - | - | - | - |
|  |  | Moisture and Landform | 13 | 88.38 | 0.00 | -1887.77 | 0.34 | 0.93 | -4.99 * | 0.05 | -3.1 * | - | -0.26 * | 0.02 | - | -0.07 | - | - | - |
|  |  | Elevation | 10 | 100.79 | 0.00 | -1897.04 | 0.34 | 0.93 | -4.93 * | 0.36 * | -2.94 * | - | - | - | - | - | - | - | - |
|  |  | Null | 9 | 103.26 | 0.00 | -1899.30 | 0.34 | 0.93 | -5.09 * | - | -3.14 * | - | - | - | - | - | - | - | - |
|  | **T_99_** | Full | 18 | 0.00 | 0.80 | -2724.59 | 0.18 | 0.32 | 4.64 * | -0.06 | -3.13 * | 0.39 * | -0.64 * | -0.11 | 0.45 * | 0.18 * | -0.62 * | -0.7 * | -0.49 * |
|  |  | Topodiversity and Vegetation | 17 | 2.83 | 0.20 | -2727.04 | 0.18 | 0.32 | 4.62 * | -0.06 | -3.1 * | 0.39 * | -0.47 * | -0.13 | 0.4 * | - | -0.62 * | -0.7 * | -0.5 * |
|  |  | Topodiversity | 14 | 35.91 | 0.00 | -2746.67 | 0.18 | 0.32 | 4.91 * | 0.15 | -3.44 * | 0.49 * | -0.29 | -0.15 | 0.32 * | - | - | - | - |
|  |  | Aspect | 11 | 51.87 | 0.00 | -2757.73 | 0.18 | 0.31 | 4.89 * | 0.26 | -3.38 * | 0.49 * | - | - | - | - | - | - | - |
|  |  | Null | 9 | 87.81 | 0.00 | -2777.74 | 0.18 | 0.32 | 4.82 * | - | -3.67 * | - | - | - | - | - | - | - | - |
|  |  | Moisture and Landform | 13 | 88.94 | 0.00 | -2774.22 | 0.18 | 0.33 | 5.01 * | -0.01 | -3.69 * | - | -0.53 * | -0.09 | - | 0.09 | - | - | - |
|  |  | Elevation | 10 | 89.22 | 0.00 | -2777.42 | 0.19 | 0.32 | 4.93 * | 0.27 | -3.53 * | - | - | - | - | - | - | - | - |
|  | **T_min_** | Full | 18 | 0.00 | 1.00 | -2487.68 | 0.11 | 0.85 | -12.51 * | 1.46 * | -0.7 | 0.14 | -0.25 | 0.17 | -0.14 | -0.28 * | 0.5 * | 0.3 * | 0.37 * |
|  |  | Topodiversity and Vegetation | 17 | 12.03 | 0.00 | -2494.73 | 0.11 | 0.85 | -12.47 * | 1.48 * | -0.75 | 0.15 * | -0.5 * | 0.21 | -0.05 | - | 0.51 * | 0.3 * | 0.38 * |
|  |  | Moisture and Landform | 13 | 19.26 | 0.00 | -2502.46 | 0.09 | 0.84 | -12.76 * | 1.12 * | -0.61 | - | -0.35 | 0.16 | - | -0.27 * | - | - | - |
|  |  | Topodiversity | 14 | 33.28 | 0.00 | -2508.44 | 0.08 | 0.84 | -12.72 * | 1.06 * | -0.66 | 0.09 | -0.65 * | 0.22 | -0.02 | - | - | - | - |
|  |  | Elevation | 10 | 46.37 | 0.00 | -2519.08 | 0.11 | 0.84 | -12.58 * | 1.71 * | -0.32 | - | - | - | - | - | - | - | - |
|  |  | Aspect | 11 | 47.15 | 0.00 | -2518.45 | 0.11 | 0.84 | -12.58 * | 1.71 * | -0.29 | 0.08 | - | - | - | - | - | - | - |
|  |  | Null | 9 | 84.14 | 0.00 | -2538.99 | 0.05 | 0.83 | -13.35 * | - | -1.28 * | - | - | - | - | - | - | - | - |
| **Winter** | **T_mean_** | Full | 18 | 0.00 | 0.99 | -2095.69 | 0.18 | 0.91 | -8.85 * | 0.73 * | -1.79 * | 0.18 * | -0.23 | 0.09 | -0.11 * | -0.17 * | 0.27 * | 0.11 | 0.23 * |
|  |  | Topodiversity and Vegetation | 17 | 8.54 | 0.01 | -2100.99 | 0.18 | 0.91 | -8.83 * | 0.73 * | -1.82 * | 0.19 * | -0.38 * | 0.11 | -0.06 | - | 0.28 * | 0.11 | 0.24 * |
|  |  | Topodiversity | 14 | 26.18 | 0.00 | -2112.91 | 0.16 | 0.91 | -8.99 * | 0.39 | -1.82 * | 0.17 * | -0.48 * | 0.12 | -0.05 | - | - | - | - |
|  |  | Moisture and Landform | 13 | 28.35 | 0.00 | -2115.01 | 0.17 | 0.91 | -9 * | 0.47 * | -1.83 * | - | -0.28 * | 0.09 | - | -0.15 * | - | - | - |
|  |  | Aspect | 11 | 41.73 | 0.00 | -2123.75 | 0.18 | 0.91 | -8.91 * | 0.86 * | -1.56 * | 0.16 * | - | - | - | - | - | - | - |
|  |  | Elevation | 10 | 50.68 | 0.00 | -2129.25 | 0.18 | 0.91 | -8.89 * | 0.87 * | -1.62 * | - | - | - | - | - | - | - | - |
|  |  | Null | 9 | 70.08 | 0.00 | -2139.97 | 0.16 | 0.90 | -9.29 * | - | -2.11 * | - | - | - | - | - | - | - | - |
|  | **GDD_5_** | Topodiversity | 14 | 0.00 | 0.48 | -2245.28 | 0.02 | 0.08 | 1.4 * | 0.04 | -1.36 * | 0.19 * | -0.21 * | 0.02 | -0.03 | - | - | - | - |
|  |  | Topodiversity and Vegetation | 17 | 1.56 | 0.22 | -2242.97 | 0.02 | 0.08 | 1.36 * | 0.04 | -1.29 * | 0.18 * | -0.25 * | 0.02 | -0.01 | - | -0.11 | -0.13 * | -0.04 |
|  |  | Aspect | 11 | 1.75 | 0.20 | -2249.23 | 0.02 | 0.08 | 1.44 * | 0.29 | -1.24 * | 0.19 * | - | - | - | - | - | - | - |
|  |  | Full | 18 | 2.98 | 0.11 | -2242.65 | 0.02 | 0.08 | 1.37 * | 0.05 | -1.29 * | 0.18 * | -0.27 * | 0.03 | 0 | 0.03 | -0.11 | -0.13 * | -0.04 |
|  |  | Moisture and Landform | 13 | 31.16 | 0.00 | -2261.89 | 0.02 | 0.08 | 1.42 * | 0.05 | -1.47 * | - | -0.25 * | 0.03 | - | 0.04 | - | - | - |
|  |  | Elevation | 10 | 33.45 | 0.00 | -2266.11 | 0.02 | 0.08 | 1.45 * | 0.29 | -1.33 * | - | - | - | - | - | - | - | - |
|  |  | Null | 9 | 34.19 | 0.00 | -2267.49 | 0.02 | 0.08 | 1.33 * | - | -1.49 * | - | - | - | - | - | - | - | - |
|  | **T_range_** | Full | 18 | 0.00 | 1.00 | -2193.60 | 0.39 | 0.64 | 7.61 * | -1.23 * | -2.05 * | 0.19 * | -0.14 | -0.22 | 0.19 * | 0.27 * | -0.46 * | -0.4 * | -0.3 * |
|  |  | Topodiversity and Vegetation | 17 | 16.10 | 0.00 | -2202.69 | 0.38 | 0.63 | 7.56 * | -1.26 * | -2.02 * | 0.18 * | 0.1 | -0.26 | 0.11 | - | -0.47 * | -0.4 * | -0.31 * |
|  |  | Topodiversity | 14 | 38.62 | 0.00 | -2217.04 | 0.35 | 0.59 | 7.75 * | -1.09 * | -2.21 * | 0.24 * | 0.21 | -0.27 | 0.06 | - | - | - | - |
|  |  | Moisture and Landform | 13 | 39.42 | 0.00 | -2218.47 | 0.36 | 0.61 | 7.84 * | -1.1 * | -2.34 * | - | -0.06 | -0.23 | - | 0.24 * | - | - | - |
|  |  | Aspect | 11 | 40.90 | 0.00 | -2221.25 | 0.36 | 0.60 | 7.61 * | -1.44 * | -2.4 * | 0.25 * | - | - | - | - | - | - | - |
|  |  | Elevation | 10 | 56.06 | 0.00 | -2229.85 | 0.37 | 0.61 | 7.63 * | -1.42 * | -2.49 * | - | - | - | - | - | - | - | - |
|  |  | Null | 9 | 92.39 | 0.00 | -2249.04 | 0.31 | 0.60 | 8.23 * | - | -1.74 * | - | - | - | - | - | - | - | - |


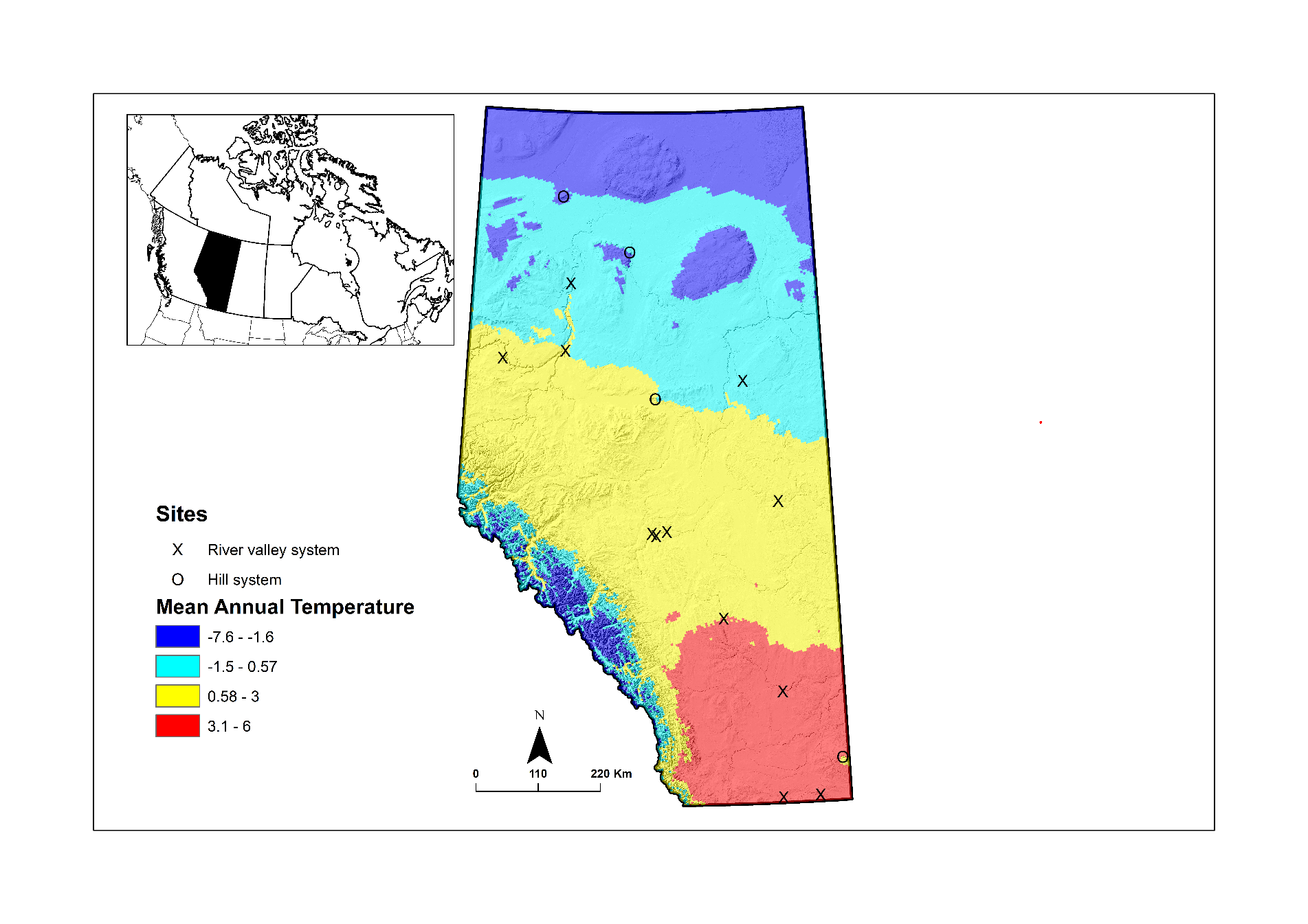


**(^o^C)**

**Fig. S1: mean annual temperature gradient and location of sampling sites in river and hill systems in Alberta, Canada. Mean annual temperature refers**


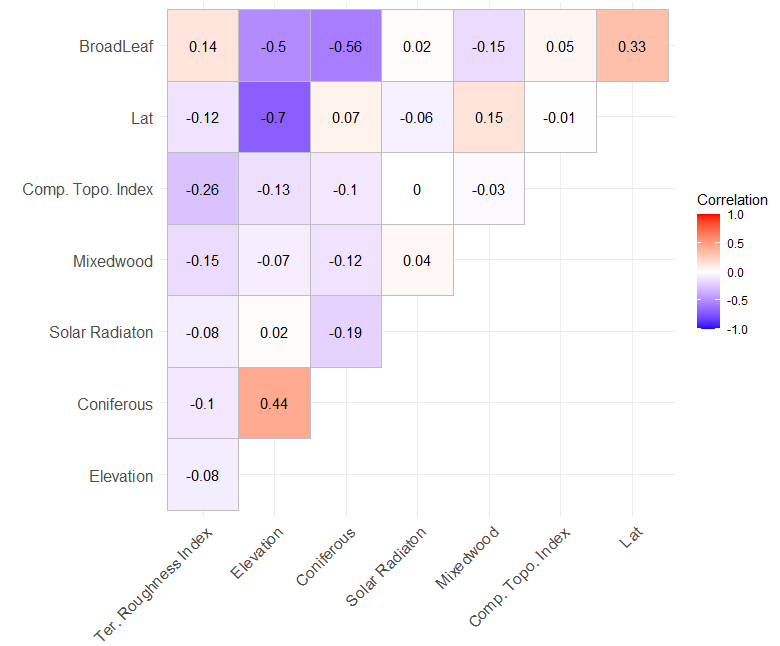


**Fig. S2: Correlation between continuous exploratory variables used in the linear mixed models. Comp. Topo. Index = Compound Topographic Index, Ter. Roughness Index = Terrain Roughness Index, Lat = Latitude. Please refer to the main text for details about each variable.**


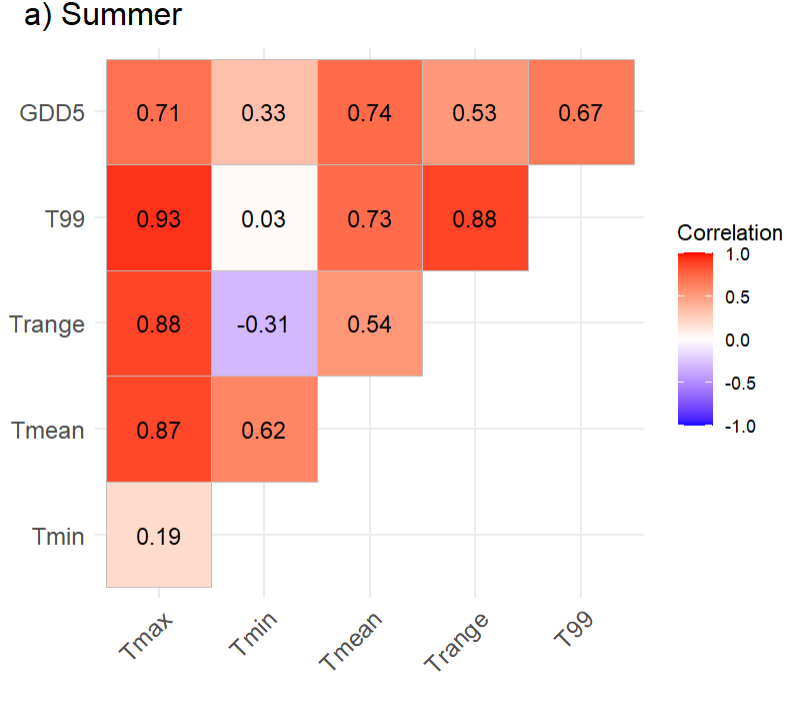


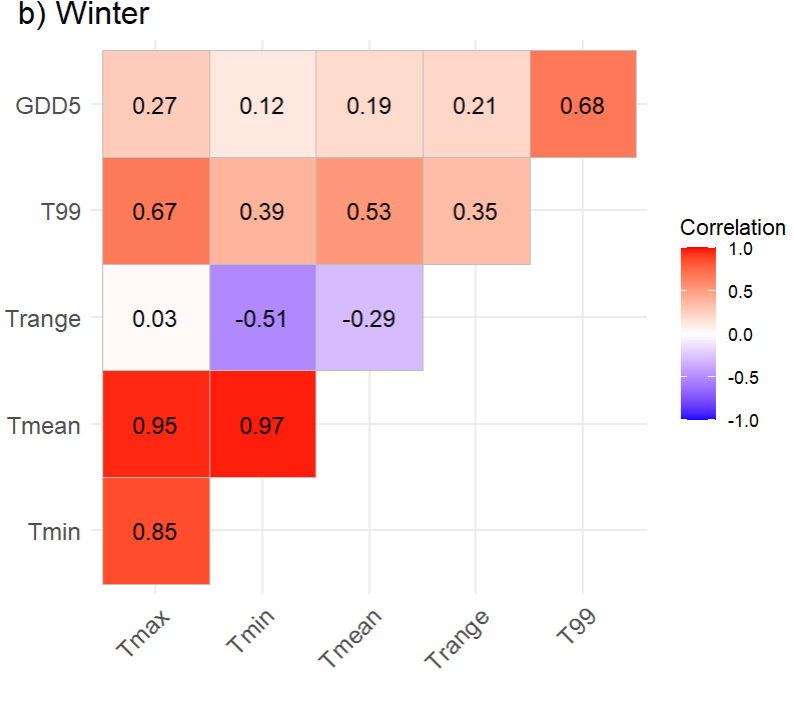


**Fig. S3: Correlation between continuous response variables used in the linear mixed models for the summer (a) and winter (b). Metrics represent monthly averages of daily maxima (T_max_), minima (T_min_), mean (T_mean_), the 99^th^ percentile of daily T_max_, and growing degree days above 5 ^o^C (GDD5). Please refer to the main text for details about each metric.**


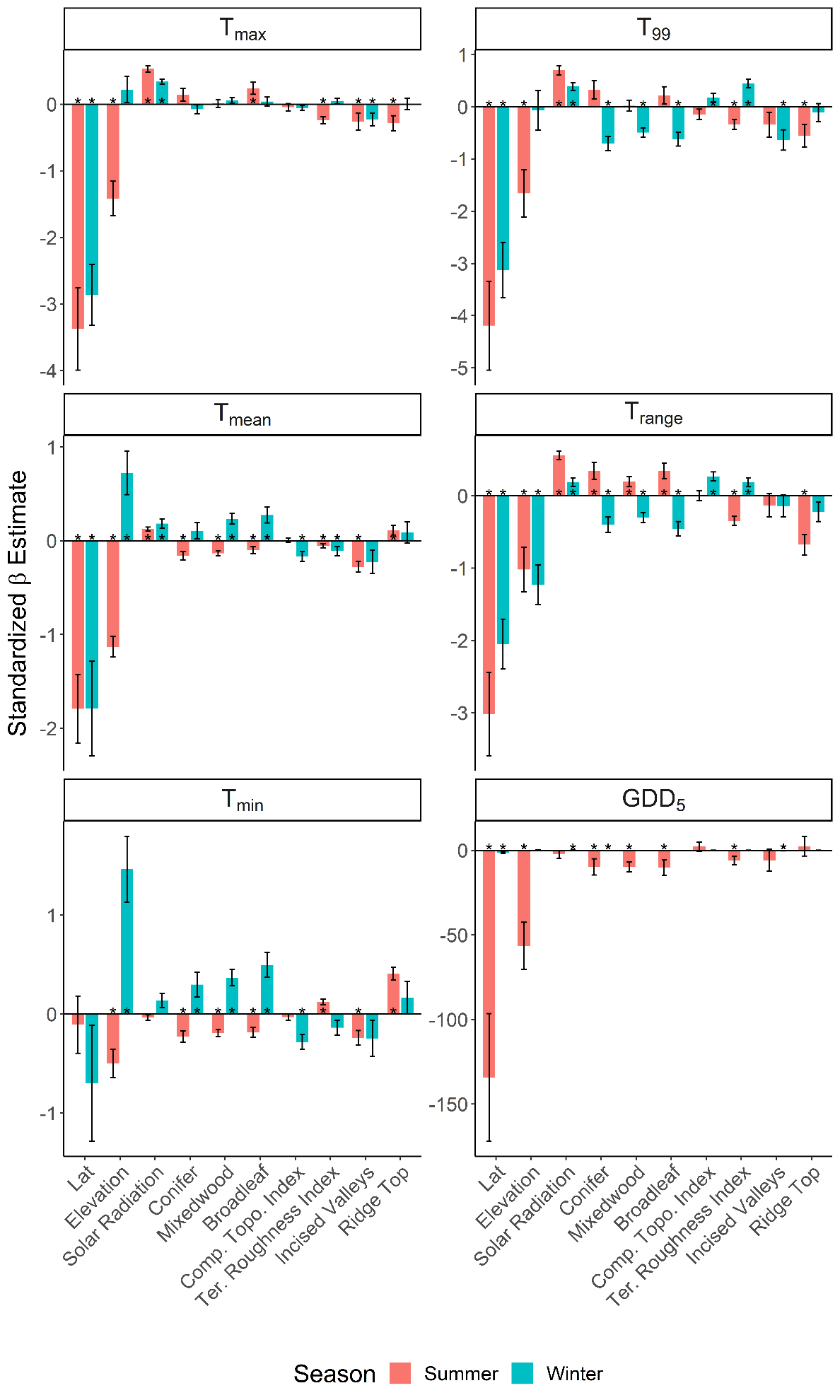


[**Fig. S**](#suppl_allbetas)**4: Standardized beta coefficients of all variables and temperature metrics of the full model for the summer and winter seasons over river valley and hill systems in Alberta, Canada. Error bars represent standard errors and * indicate significant standardized estimates at ɑ=0.05. An explanation for each temperature metric can be found in the main text.**

##
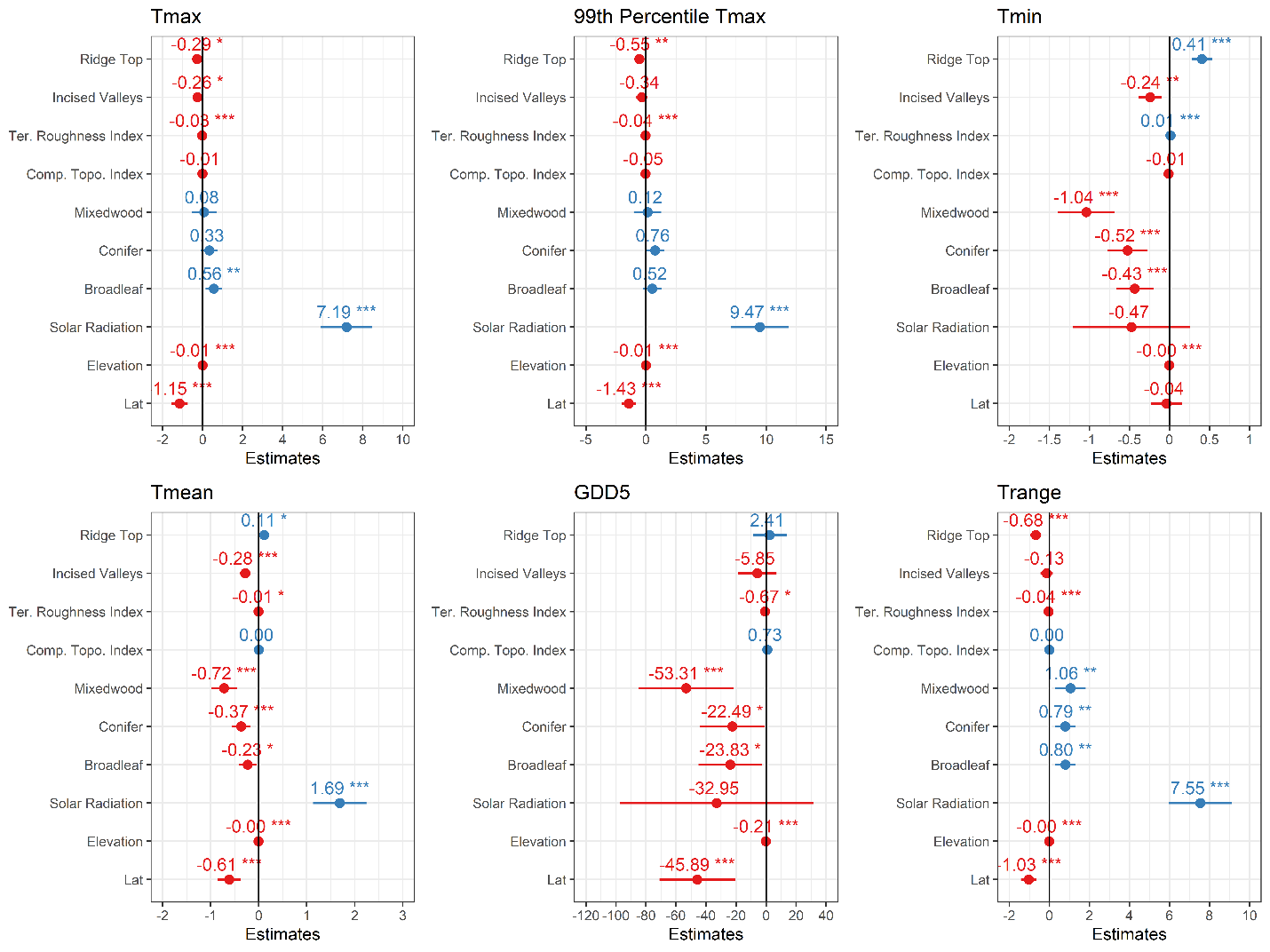


**Fig. S5: Unstandardized beta coefficients for topographic and vegetation cover effects on summer temperatures (2014-2020) at river valley and hill sites in Alberta, Canada. Refer to the main text for an explanation of each temperature metric. Bars indicate 95% confidence intervals with estimated coefficient on top of each line. Asterisks indicate significant estimates where the p-value is between 0 - 0.001 (‘***’), 0.001 - 0.01 (‘**’), and 0.01 - 0.05 (‘*’).**


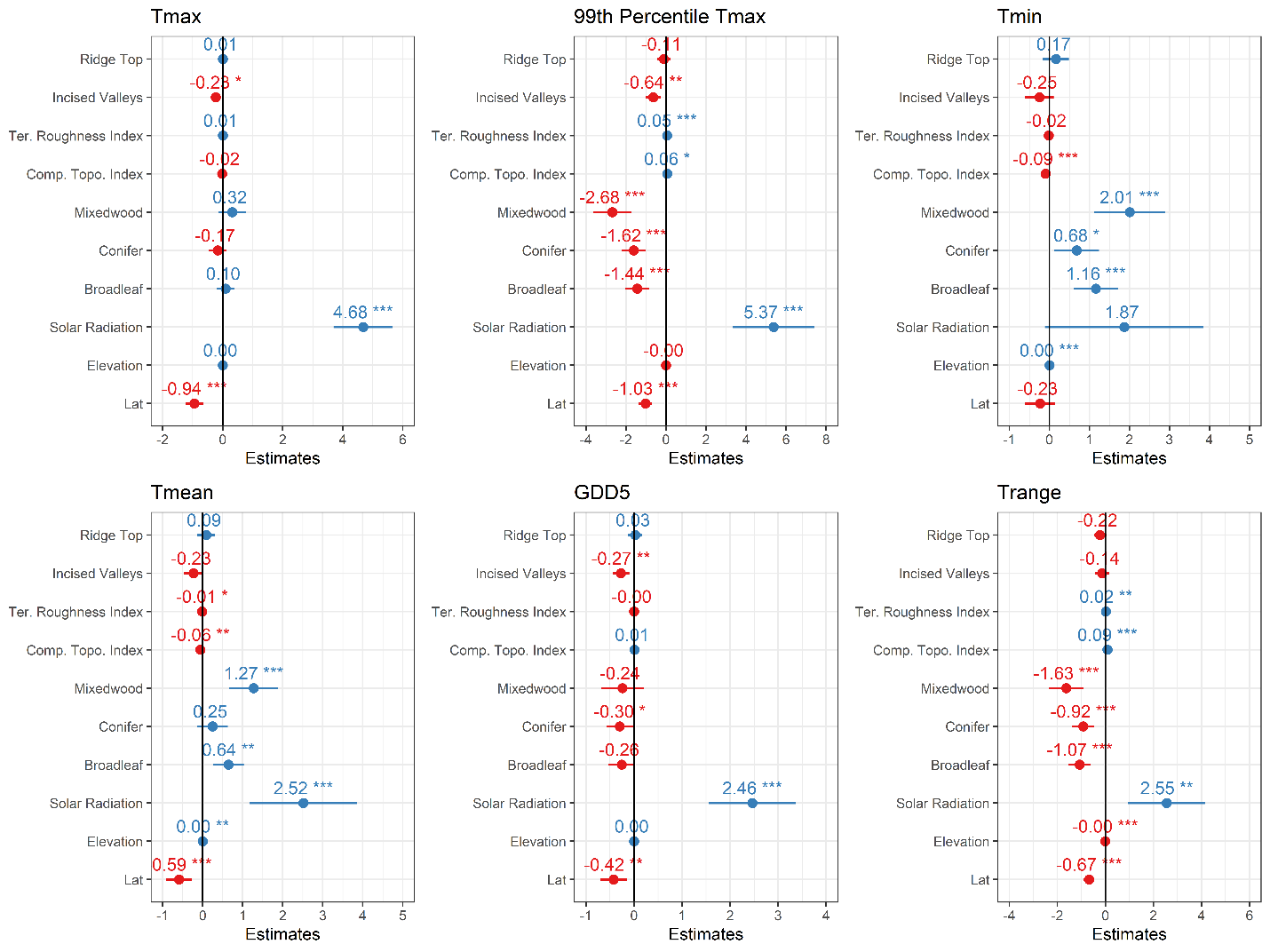


**Fig. S6: Unstandardized beta coefficients for topographic and vegetation cover effects on winter temperatures (2014-2020) at river valley and hill sites in Alberta, Canada. Refer to the main text for explanation of each temperature metric. Bars indicate 95% confidence intervals with estimated coefficient on top of each line. Asterisks indicate significant estimates where the p-value is between 0 - 0.001 (‘***’), 0.001 - 0.01 (‘**’), and 0.01 - 0.05 (‘*’).**
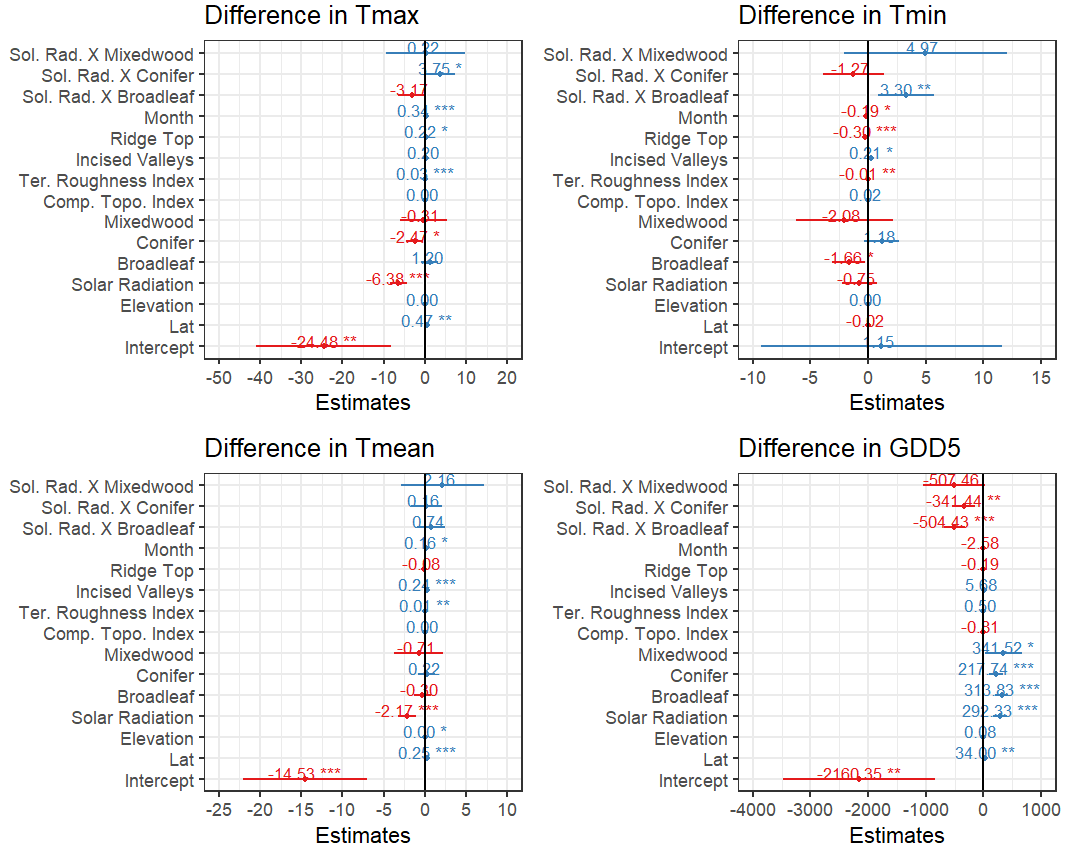


**Fig. S7: Unstandardized beta coefficients of different topographic variables and vegetation cover effects on the absolute difference between ClimateNA and iButton readings (i.e., T_Difference_ = T_ClimateNA_-T_iButton_) in different river valley and hill systems in Alberta, Canada during the summer 2014-2020. Refer to the main text for explanation of each temperature metric. Bars indicate 95% confidence intervals with estimated coefficient on top of each line. Asterisks indicate significant estimates where the p-value is between 0 - 0.001 ( ‘***’), 0.001 - 0.01 (‘**’), and 0.01 - 0.05 (‘*’).**


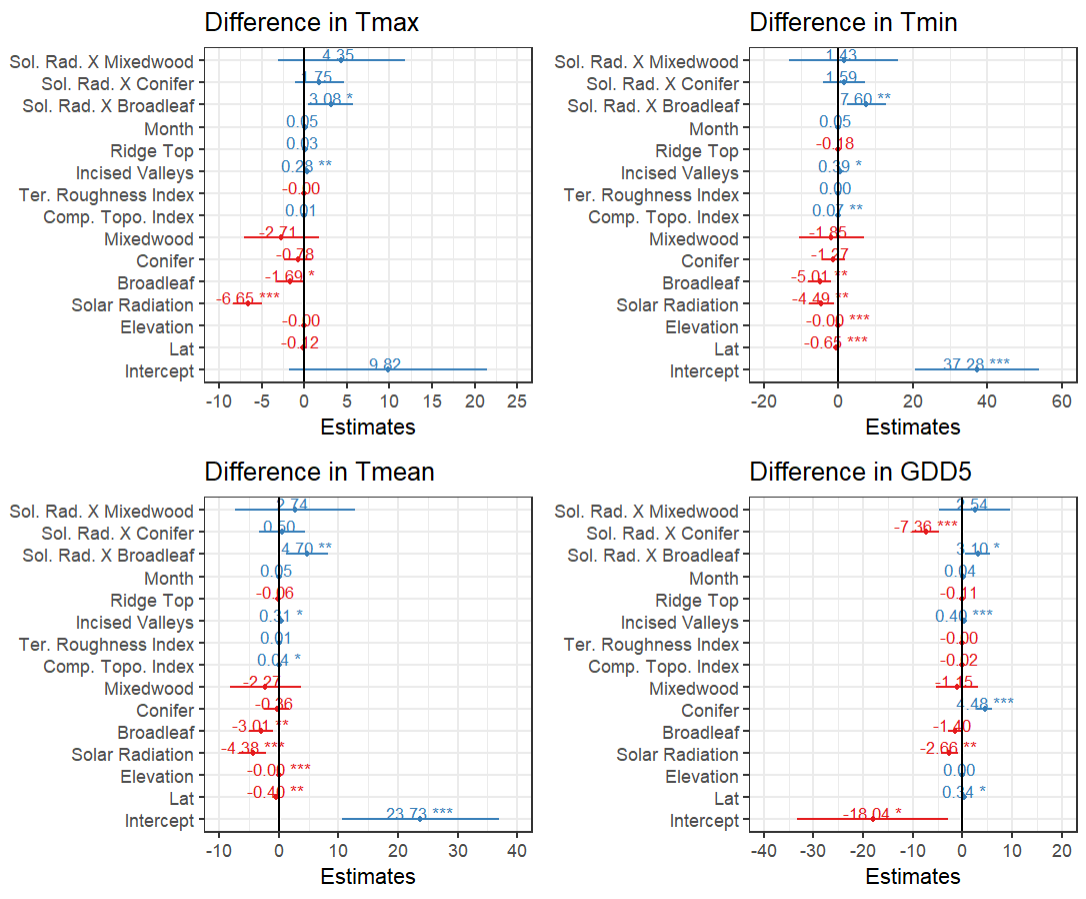


**Fig. S8: Unstandardized beta coefficients of different topographic variables and vegetation cover effects on the absolute difference between ClimateNA and iButton readings (i.e., T_Difference_ = T_ClimateNA_-T_iButton_) in different river valley and hill systems in Alberta, Canada during the winter 2014-2020. Refer to the main text for explanation of each temperature metrics. Bars indicate 95% confidence intervals with estimated coefficient on top of each line. Asterisks indicate significant estimates where the p-value is between 0 - 0.001 (‘***’), 0.001 - 0.01 (‘**’), and 0.01 - 0.05 (‘*’).**


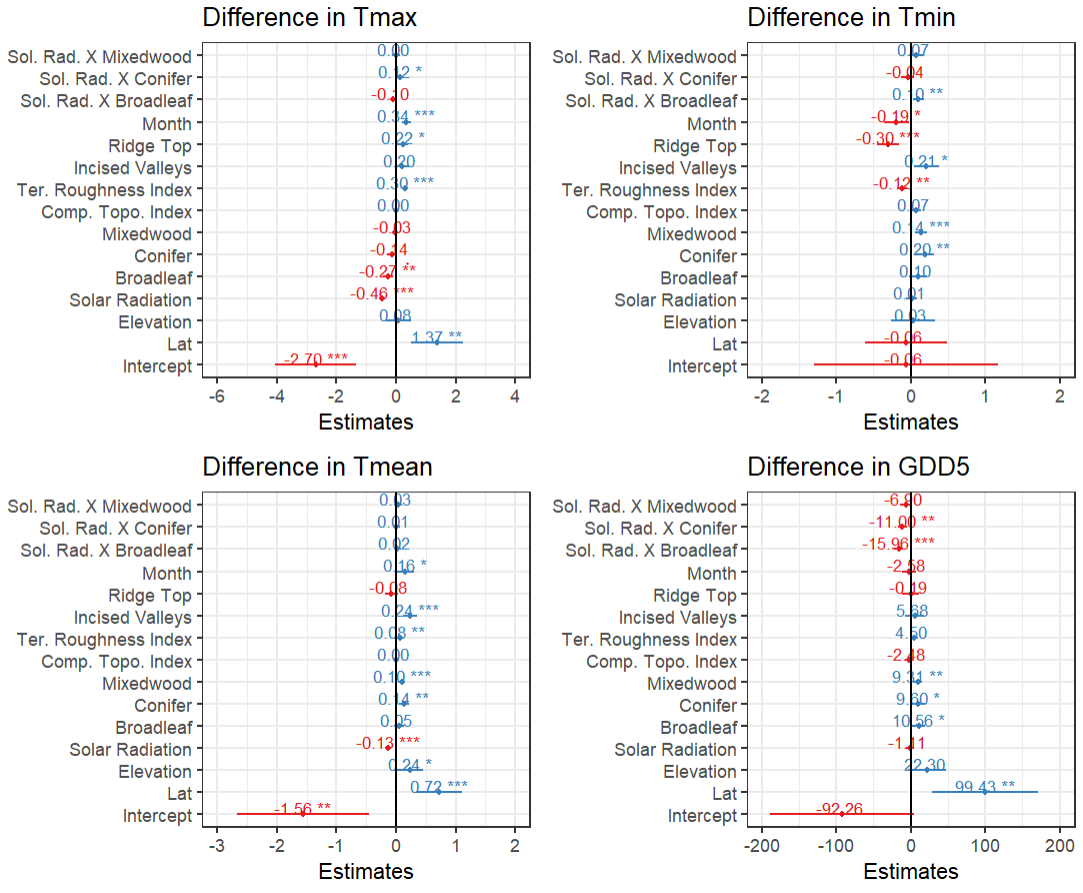


**Fig. S9: Standardized beta coefficients of different topographic variables and vegetation cover effects on the absolute difference between ClimateNA and iButton readings (i.e., T_Difference_ = T_ClimateNA_-T_iButton_) in different river valley and hill systems in Alberta, Canada during the summer 2014-2020. Refer to the main text for explanation of each temperature metrics. Bars indicate 95% confidence intervals with estimated coefficient on top of each line. Asterisks indicate significant estimates where the p-value is between 0 - 0.001 ( ‘***’), 0.001 - 0.01 (‘**’), and 0.01 - 0.05 (‘*’).**


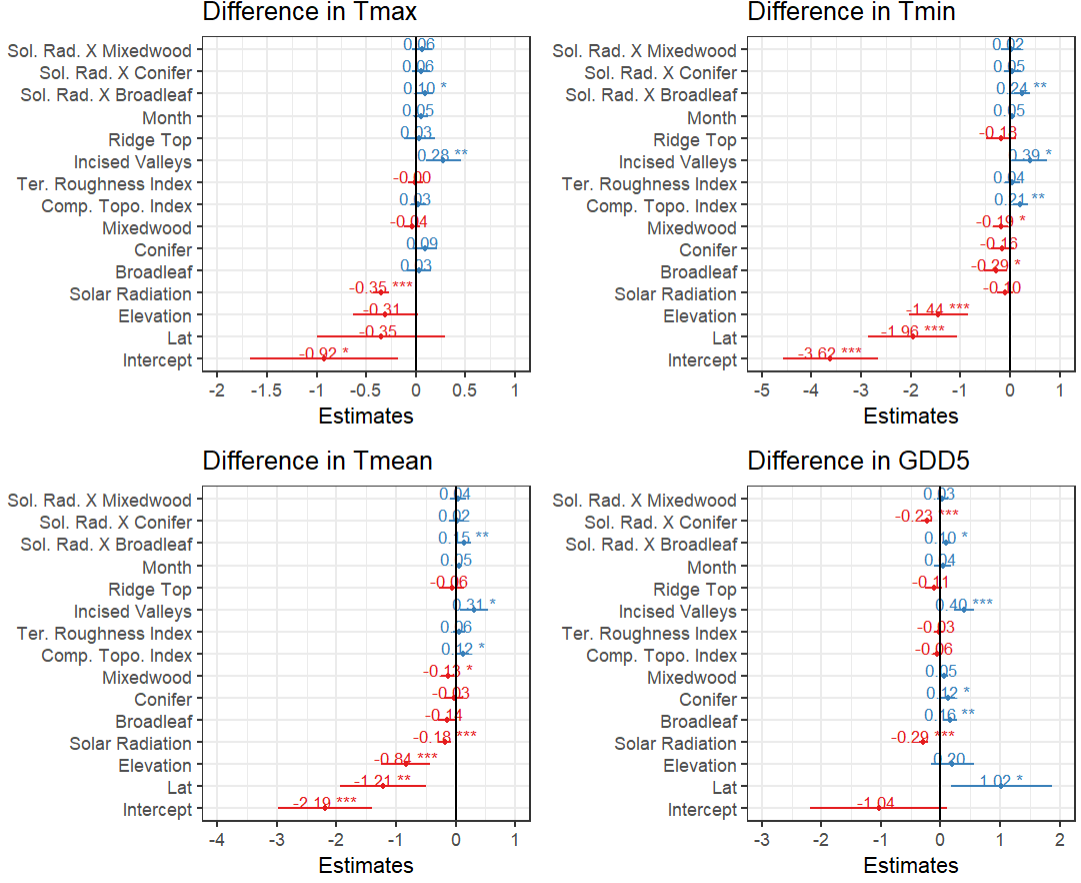
 **Fig. S10: Standardized beta coefficients of different topographic variables and vegetation cover effects on the absolute difference between ClimateNA and iButton readings (i.e., T_Difference_ = T_ClimateNA_-T_iButton_) in different river valley and hill systems in Alberta, Canada during the winter 2014-2020. Refer to the main text for explanation of each temperature metrics. Bars indicate 95% confidence intervals with estimated coefficient on top of each line. Asterisks indicate significant estimates where the p-value is between 0 - 0.001 ( ‘***’), 0.001 - 0.01 (‘**’), and 0.01 - 0.05 (‘*’).**

**
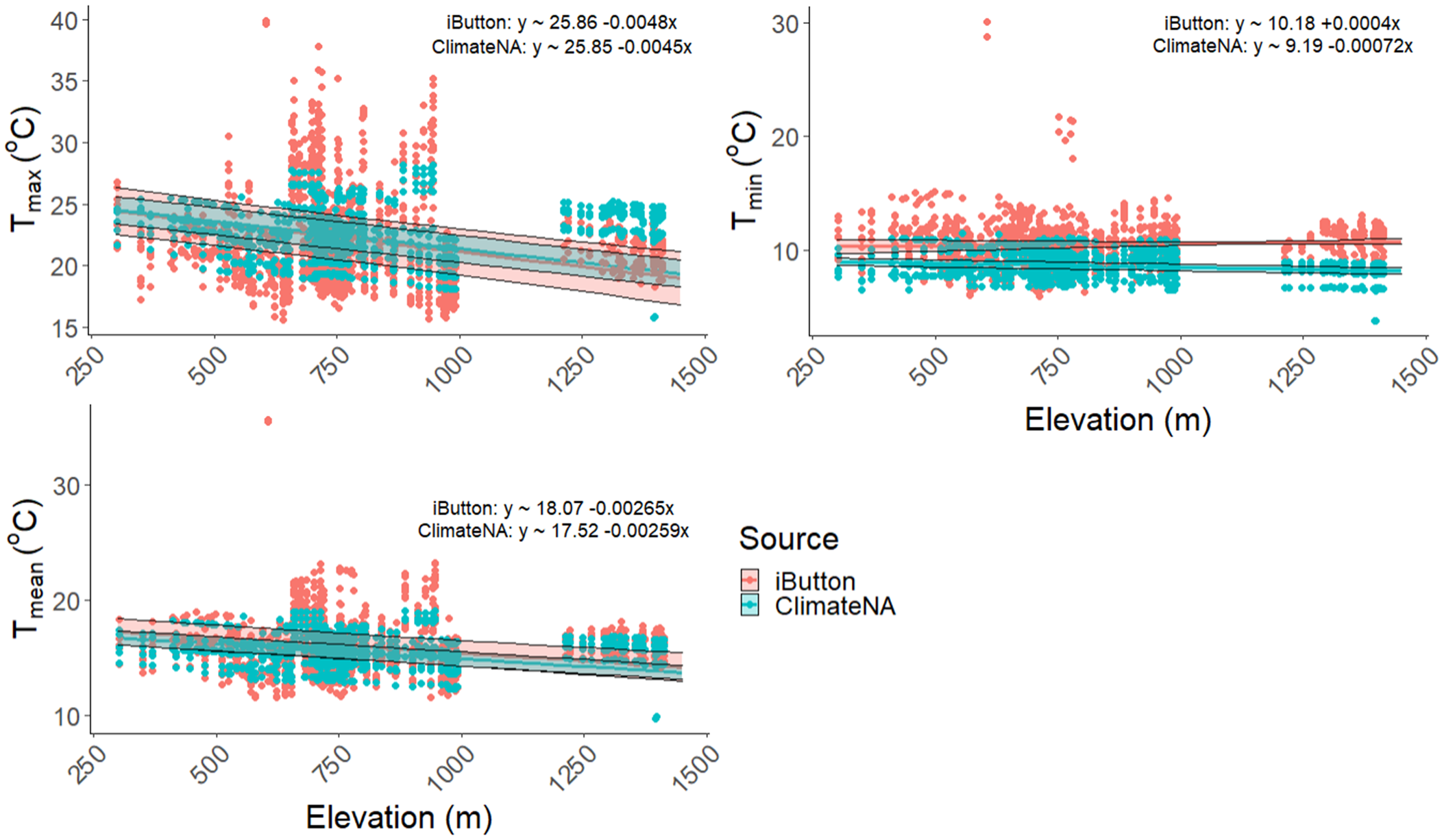
 Fig. S11: The effect of elevation over ClimateNA and iButton readings monthly average of daily maxima (T_max_), minima (T_min_) and mean (T_mean_) in different river valley and hill systems in Alberta, Canada during the summer months of 2014-2020. Different sources of data are plotted with different colors.**
