# Supplementary material for: Topographic and vegetation drivers of thermal heterogeneity along the boreal–grassland transition zone in western Canada: Implications for climate change refugia
Source: Ecol Evol. 2022 Jun 22;12(6):e9008. doi: 10.1002/ece3.9008 (PMC9217894; doi:10.1002/ece3.9008)
Supplement: Supplementary file 2 — Appendix S2 [file ECE3-12-e9008-s001.docx]

**Topographic and vegetation drivers of thermal refugia along the boreal–grassland transition zone in western Canada**

Estevo, C. A.^1^; Stralberg, D.^2^; Nielsen, S.E.^3^; Bayne, E.^1^

^1^ Department of Biological Sciences, University of Alberta, Edmonton, Canada

^2^ Natural Resources Canada, Northern Forestry Centre, Edmonton, Alberta, Canada

^3^ Department of Renewable Resources, University of Alberta, Edmonton, Canada

# Appendix S2


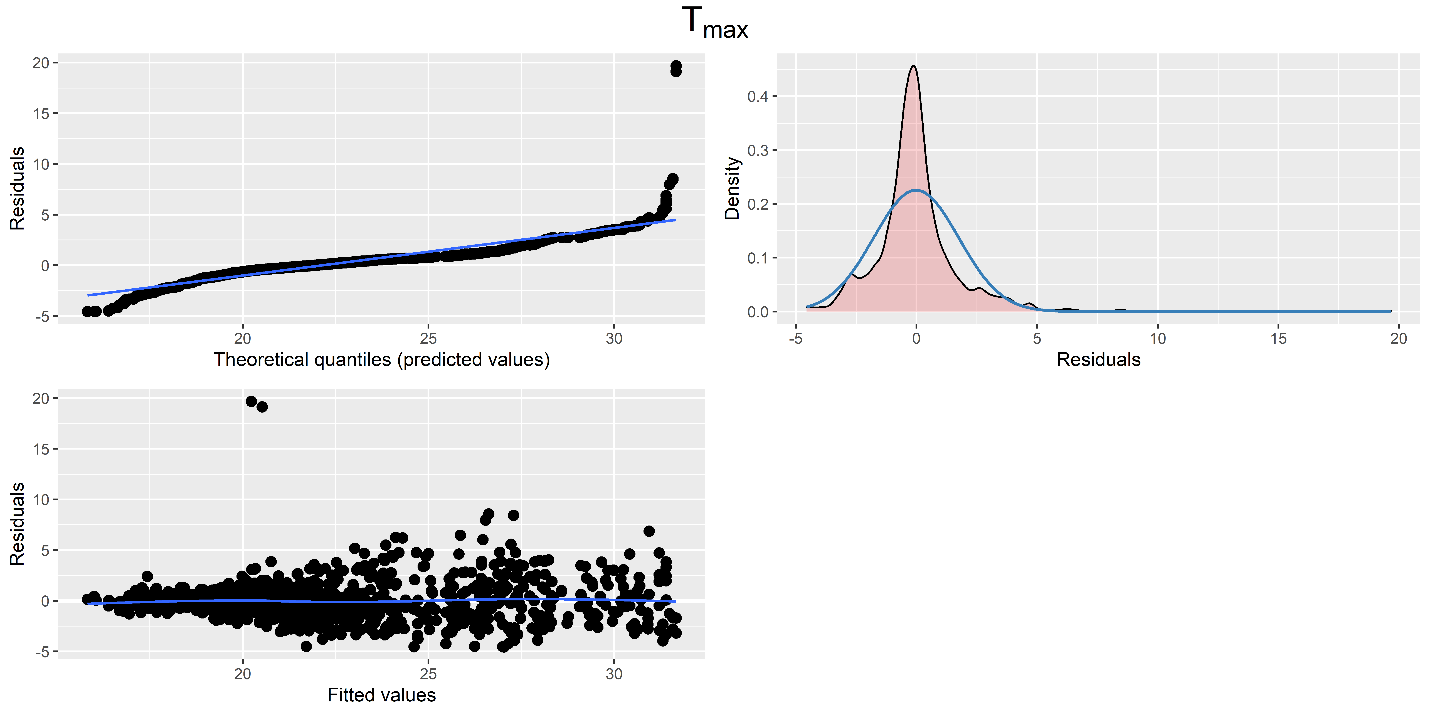
 **Figure S12: Model diagnostics for the monthly average of the daily maximum temperature for the *summer* season. Residuals are for the *full model* with *scaled* variables. Please refer to the Methods section in the main text for an explanation of each metric.**


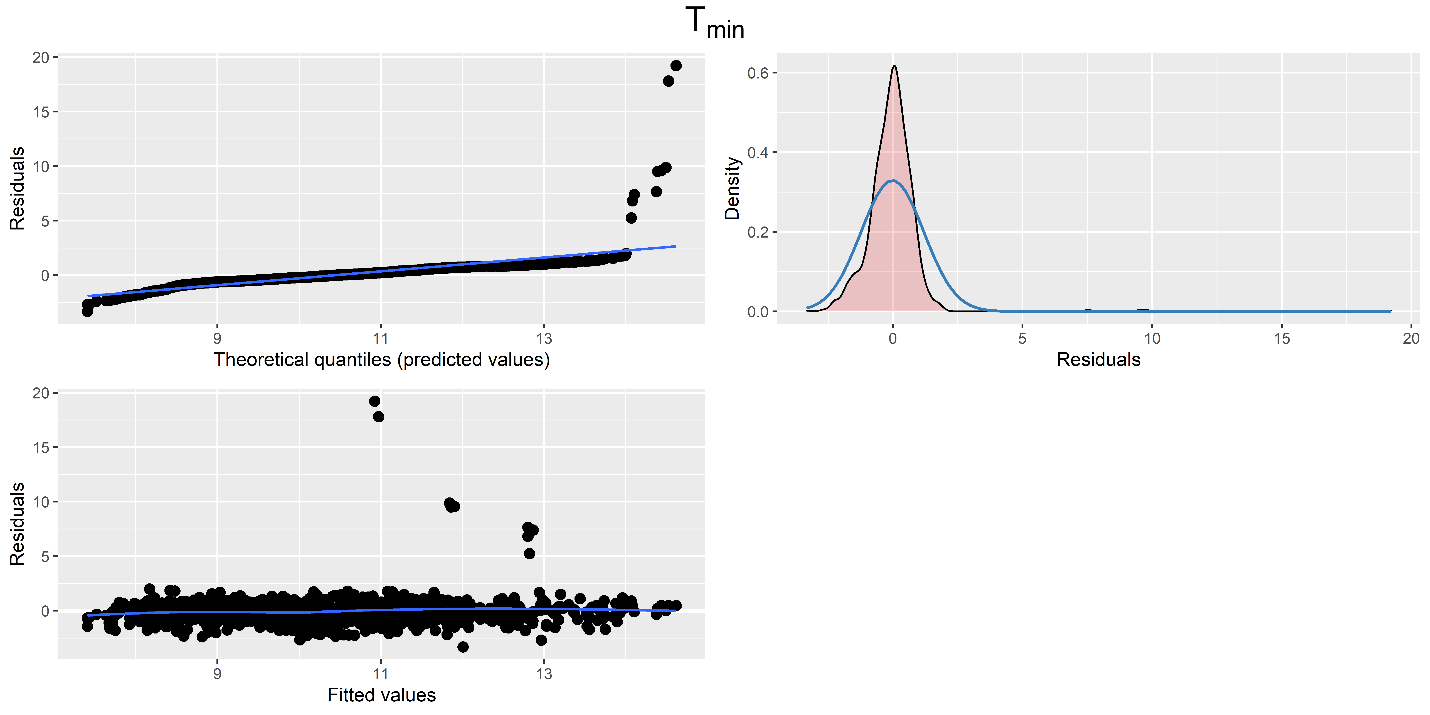
 **Figure S13: Model diagnostics for the monthly average of the daily minimum temperature for the *summer* season. Residuals are for the *full model* with *scaled* variables. Please refer to the Methods section in the main text for an explanation of each metric.**


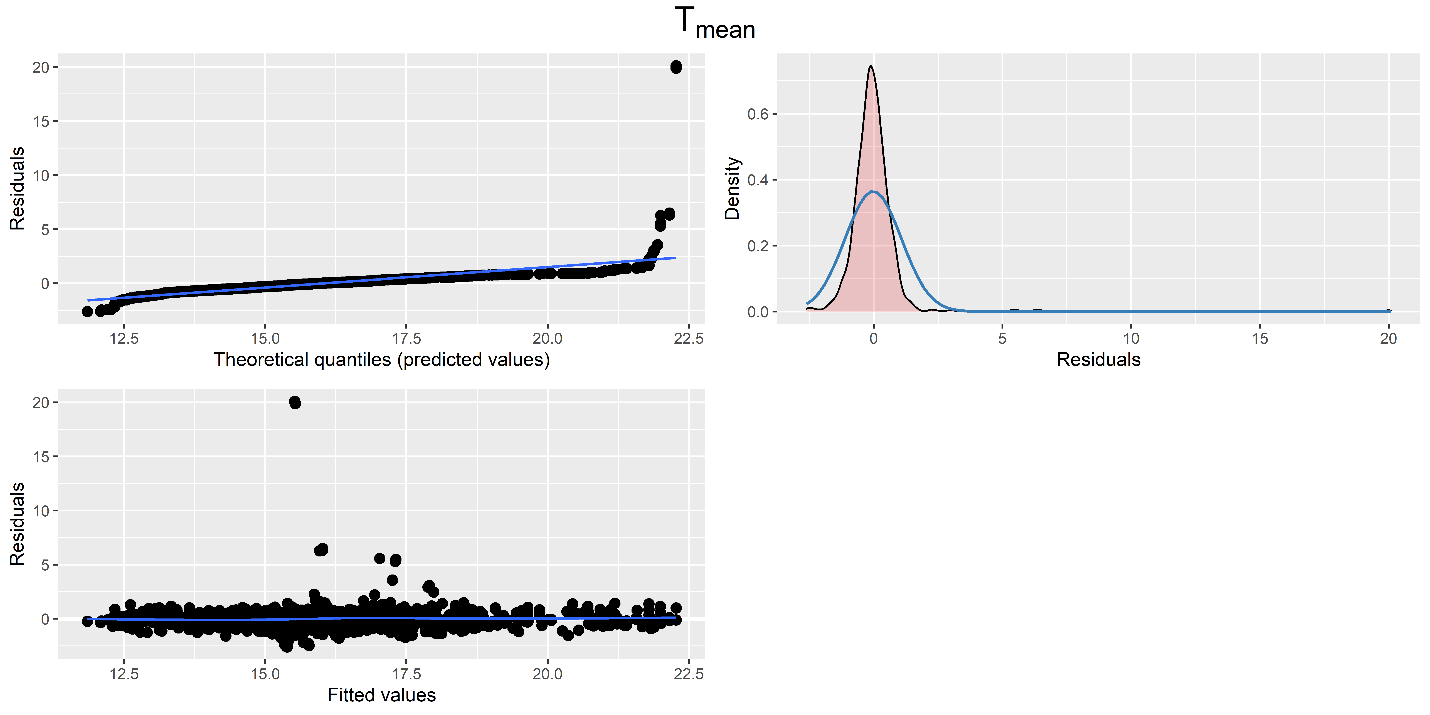
 **Figure S14: Model diagnostics for the monthly average of the daily mean temperature for the *summer* season. Residuals are for the *full model* with *scaled* variables. Please refer to the Methods section in the main text for an explanation of each metric.**


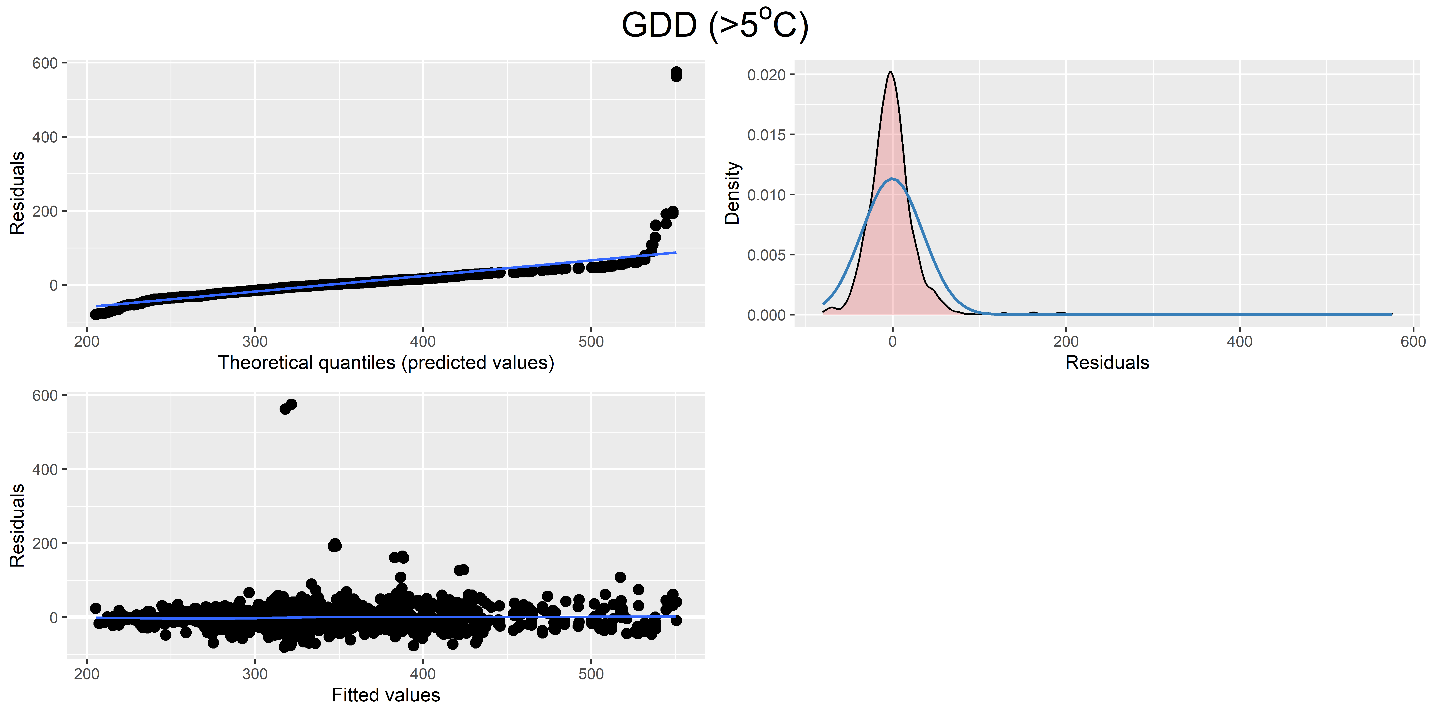
 **Figure S15: Model diagnostics for the monthly average growing degree days above 5 ^o^C for the *summer* season. Residuals are for the *full model* with *scaled* variables. Please refer to the Methods section in the main text for an explanation of each metric.**
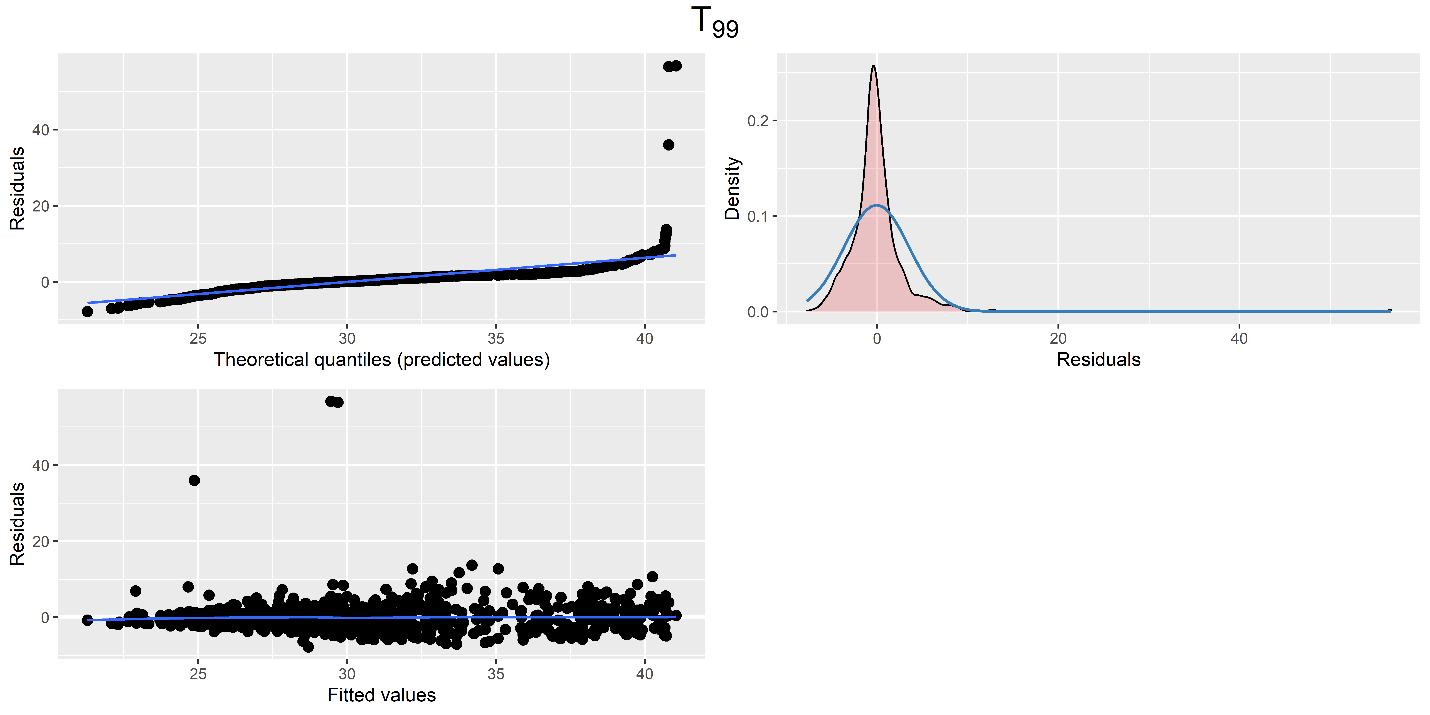
 **Figure S16: Model diagnostics for the 99^th^ percentile of daily maxima for the *summer* season. Residuals are for the *full model* with *scaled* variables. Please refer to the Methods section in the main text for an explanation of each metric.**


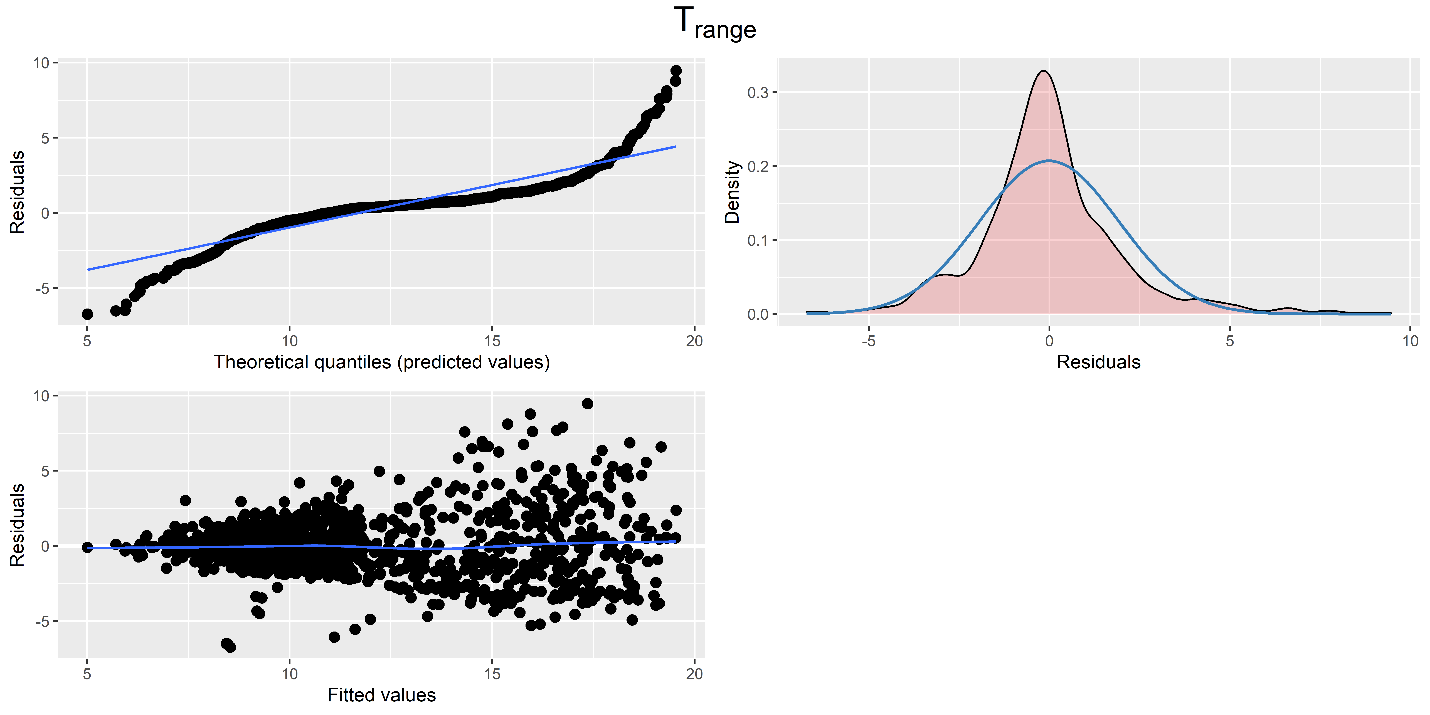
 **Figure S17: Model diagnostics for the monthly average of daily temperature range for the *summer* season. Residuals are for the *full model* with *scaled* variables. Please refer to the Methods section in the main text for an explanation of each metric.**


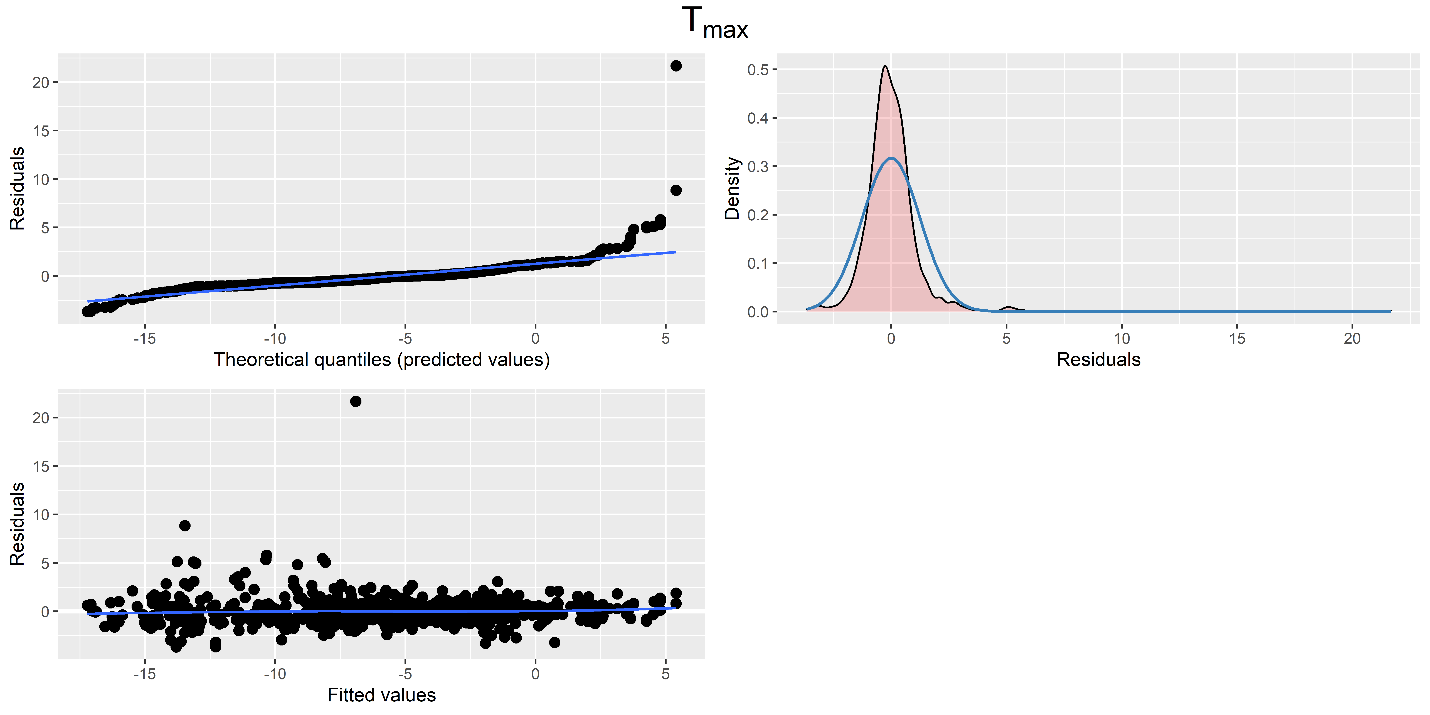

**Figure S18: Model diagnostics for the monthly average of the daily maximum temperature for the *winter* season. Residuals are for the *full model* with *scaled* variables. Please refer to the Methods section in the main text for an explanation of each metric.**


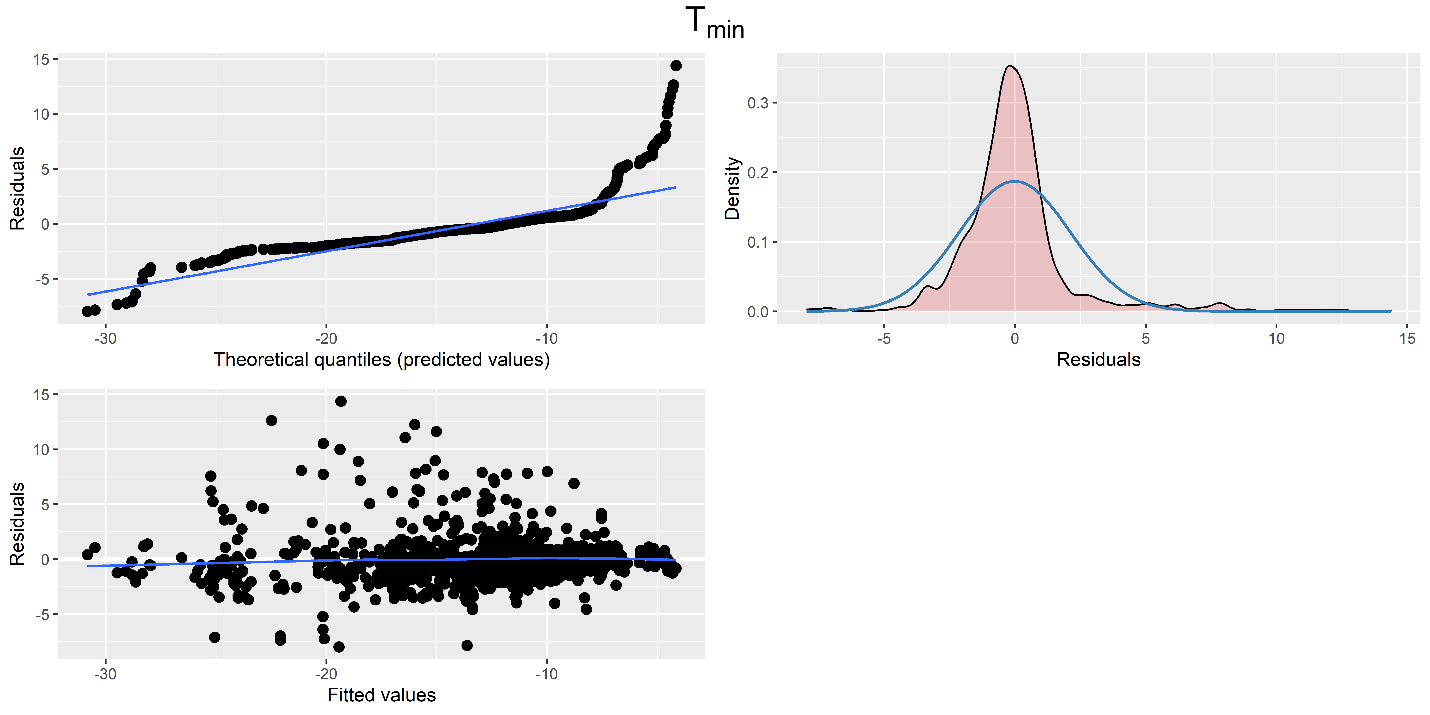

**Figure S19: Model diagnostics for the monthly average of the daily minimum temperature for the *winter* season. Residuals are for the *full model* with *scaled* variables. Please refer to the Methods section in the main text for an explanation of each metric.**
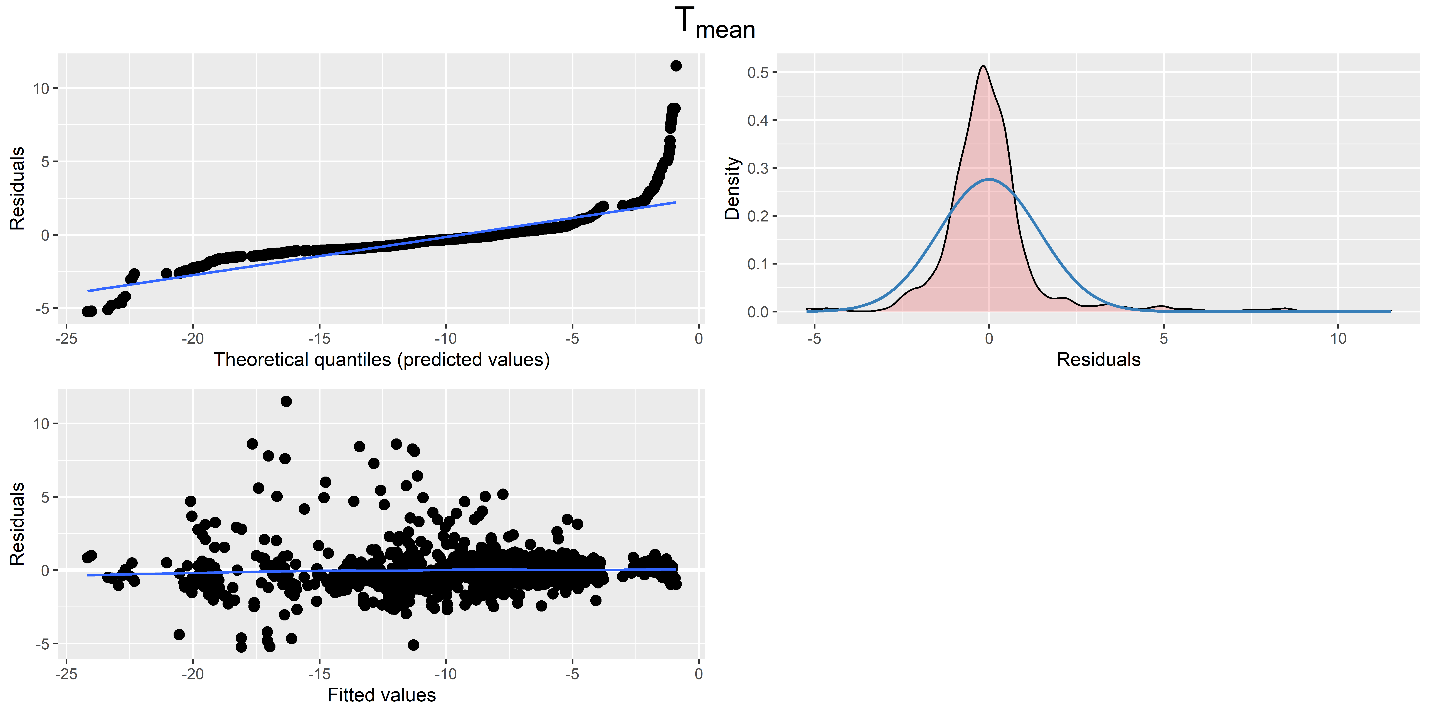
 **Figure S20: Model diagnostics for the monthly average of the daily mean temperature for the *winter* season. Residuals are for the *full model* with *scaled* variables. Please refer to the Methods section in the main text for an explanation of each metric.**


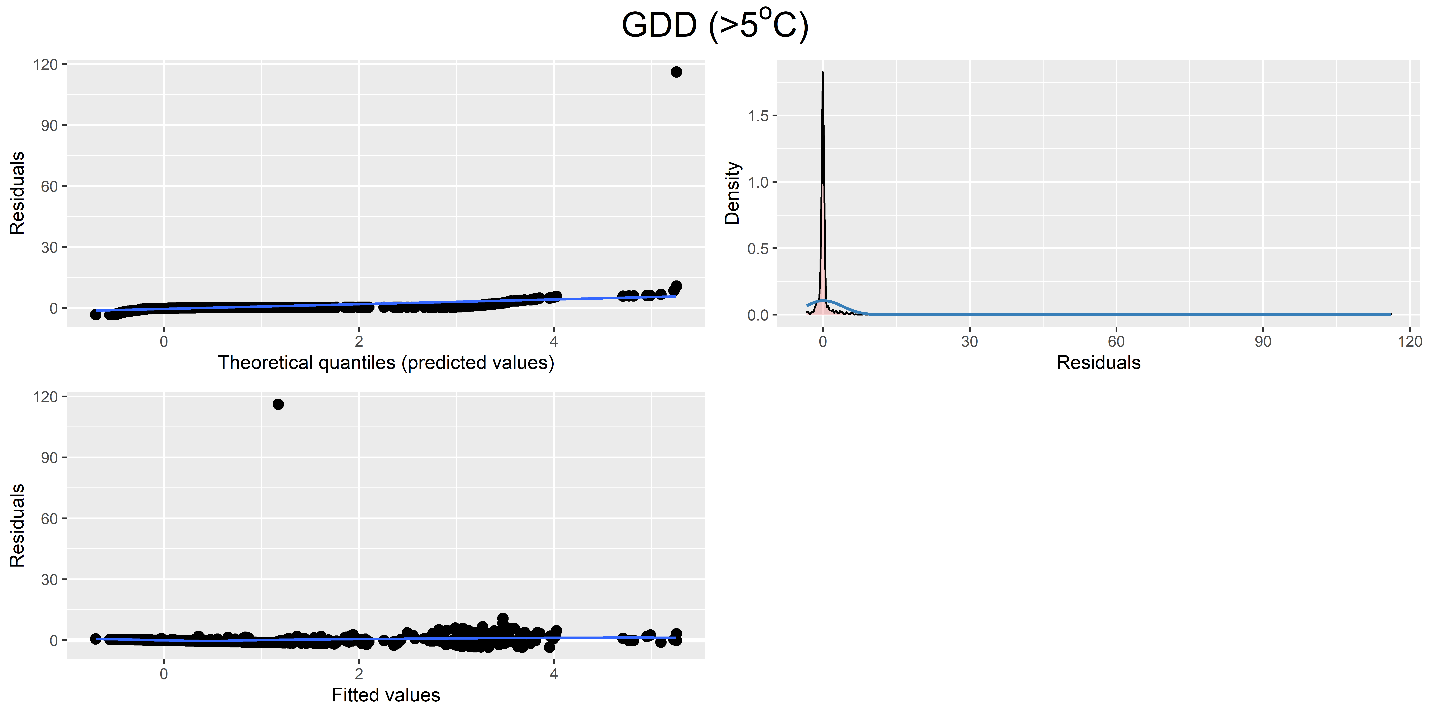
 **Figure S21: Model diagnostics for the monthly average growing degree days above 5 ^o^C for the *winter* season. Residuals are for the *full model* with *scaled* variables. Please refer to the Methods section in the main text for an explanation of each metric.**
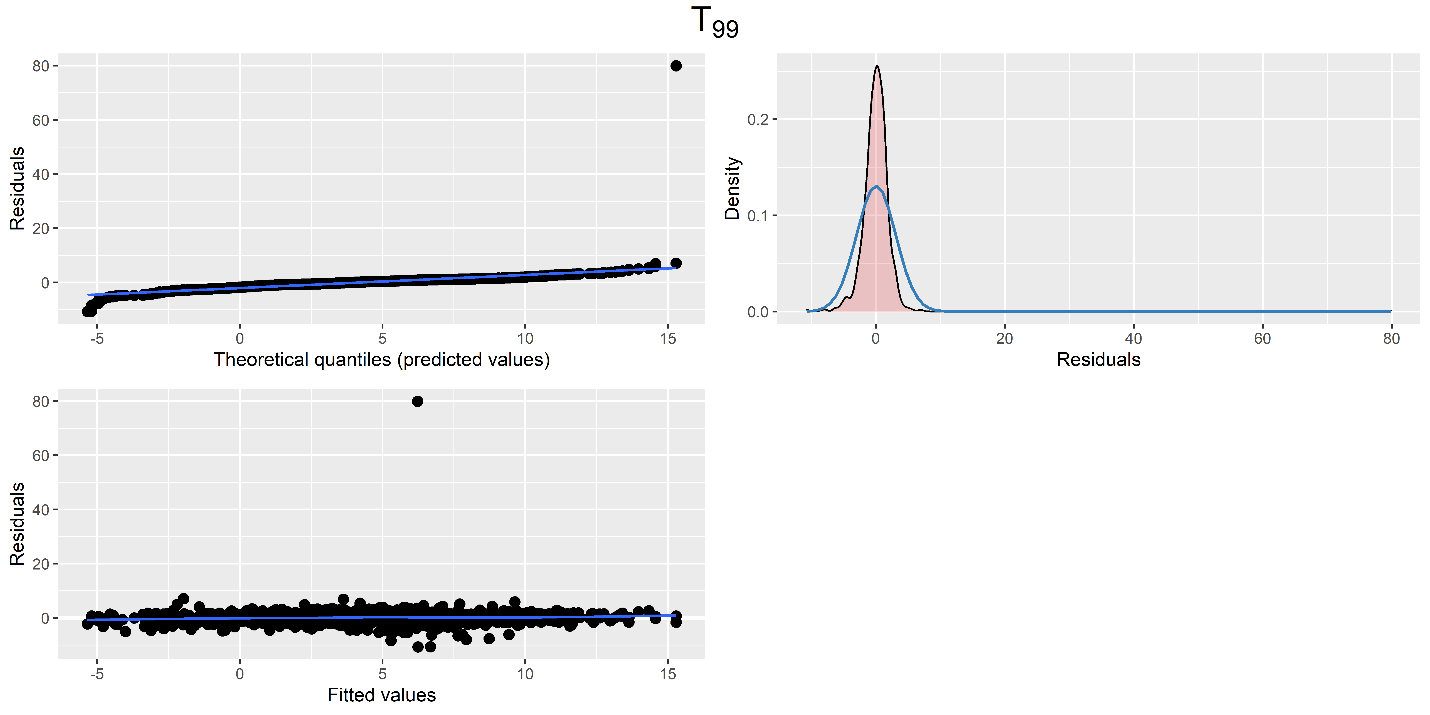
 **Figure S22: Model diagnostics for the 99^th^ percentile of daily maxima for the *winter* season. Residuals are for the *full model* with *scaled* variables. Please refer to the Methods section in the main text for an explanation of each metric.**


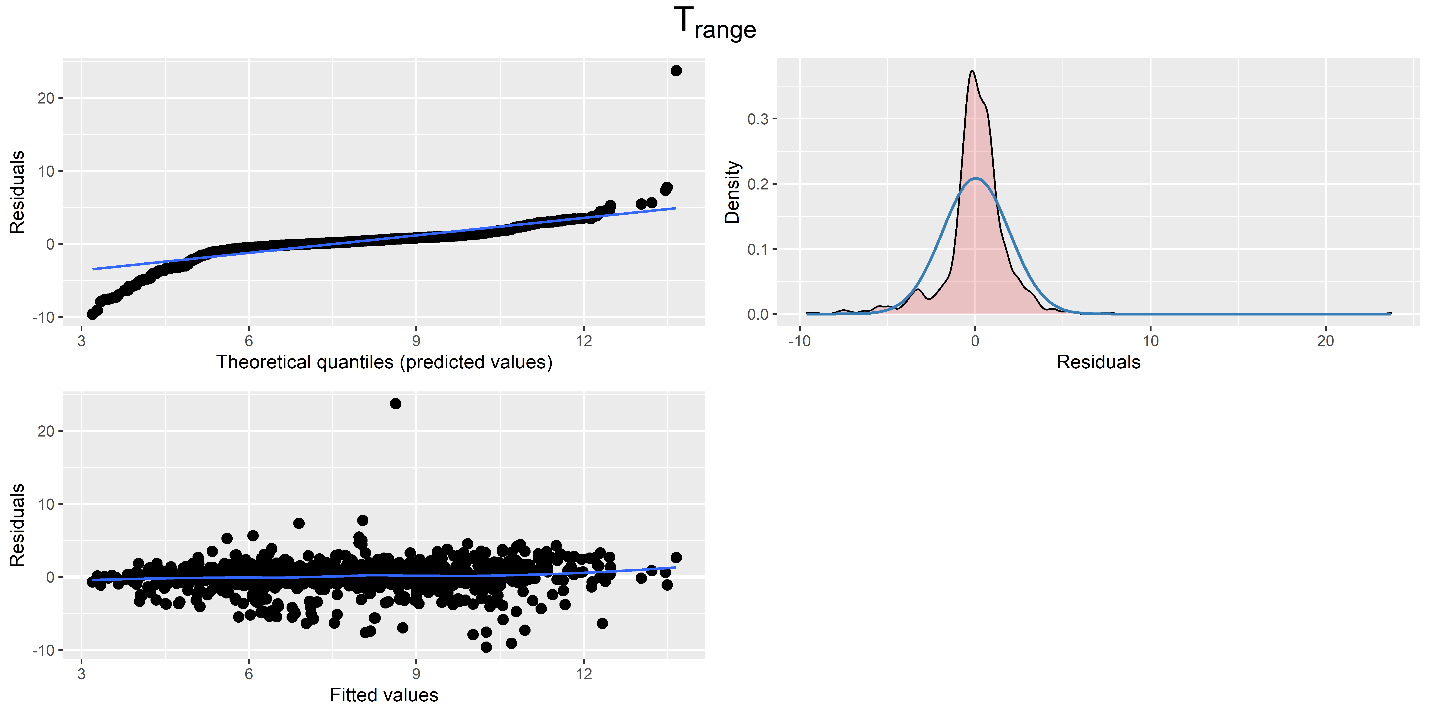
 **Figure S23: Model diagnostics for the monthly average of daily temperature range for the *winter* season. Residuals are for the *full model* with *scaled* variables. Please refer to the Methods section in the main text for an explanation of each metric.**.


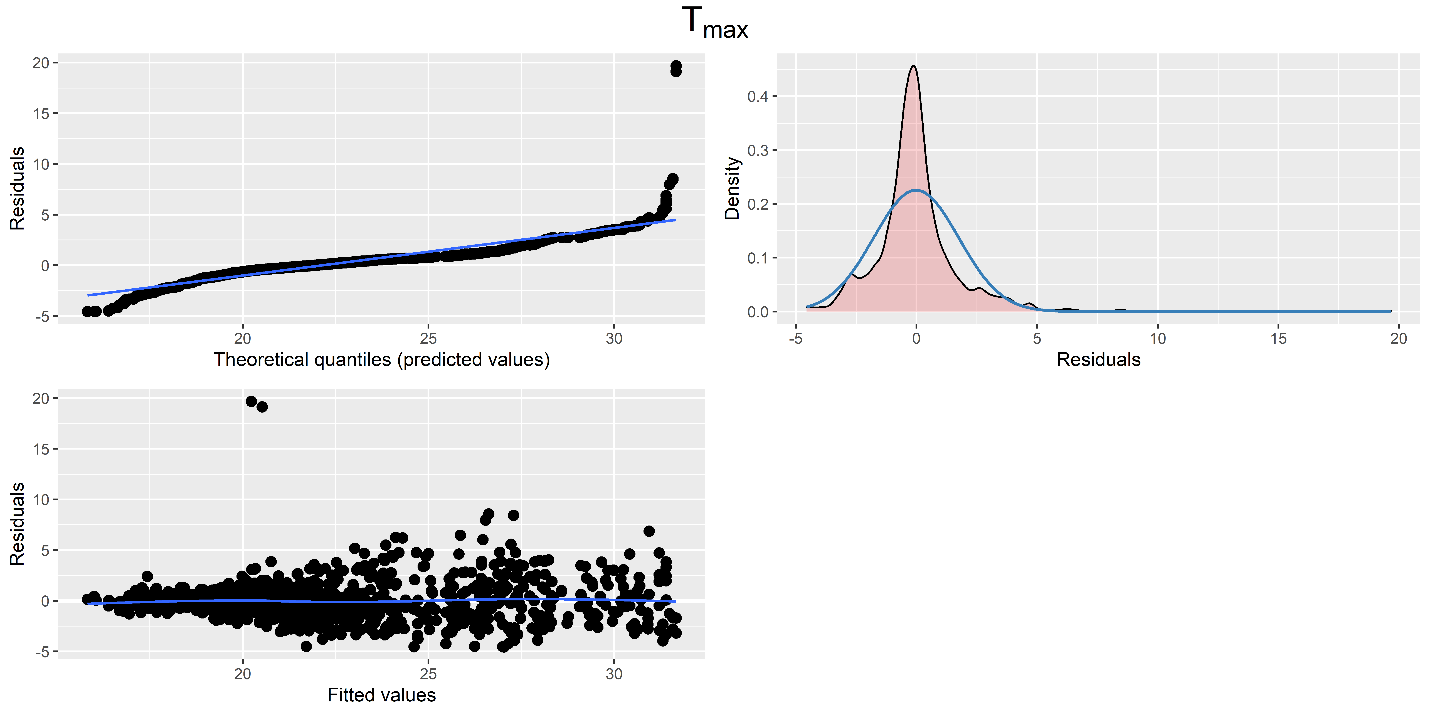

**Figure S24: Model diagnostics for the monthly average of the daily maximum temperature for the *summer* season. Residuals are for the *full model* with *unscaled* variables. Please refer to the Methods section in the main text for an explanation of each metric.**


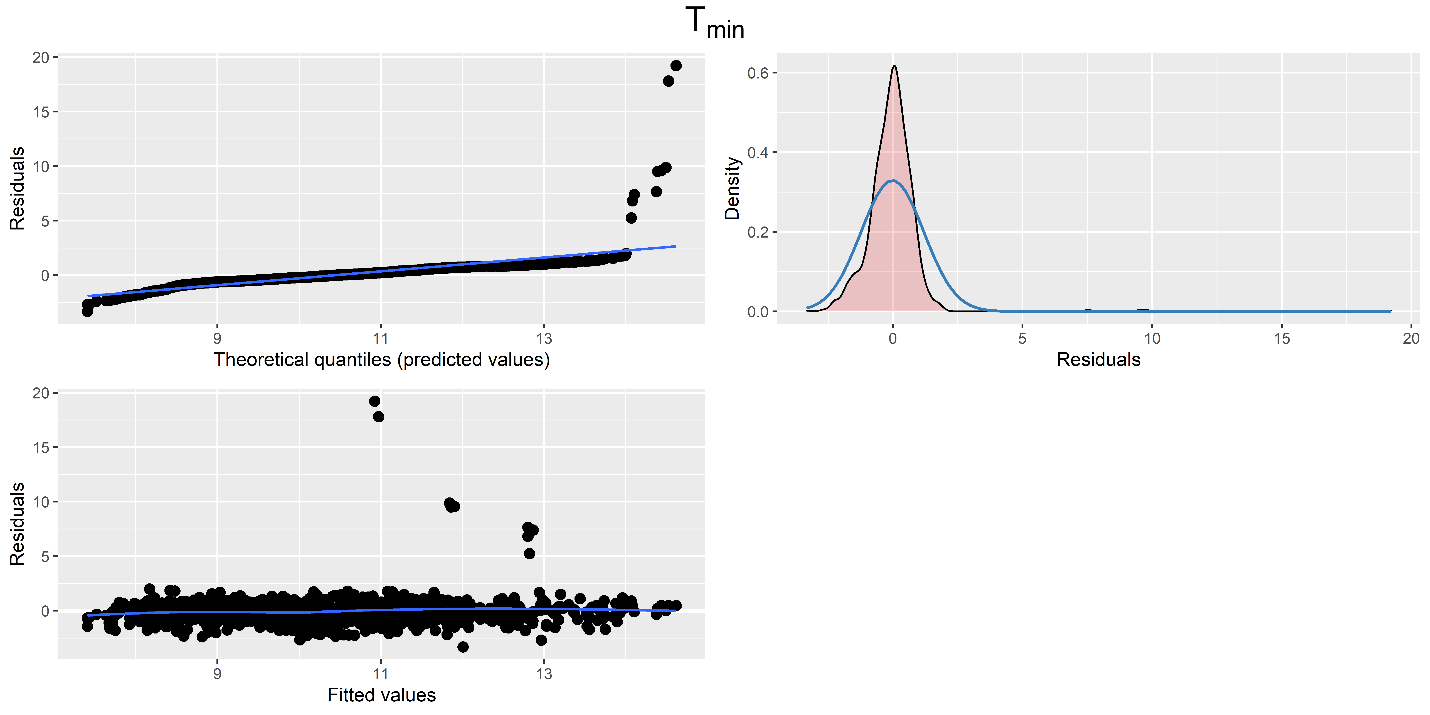

**Figure S25: Model diagnostics for the monthly average of the daily minimum temperature for the *summer* season. Residuals are for the *full model* with *unscaled* variables. Please refer to the Methods section in the main text for an explanation of each metric.**


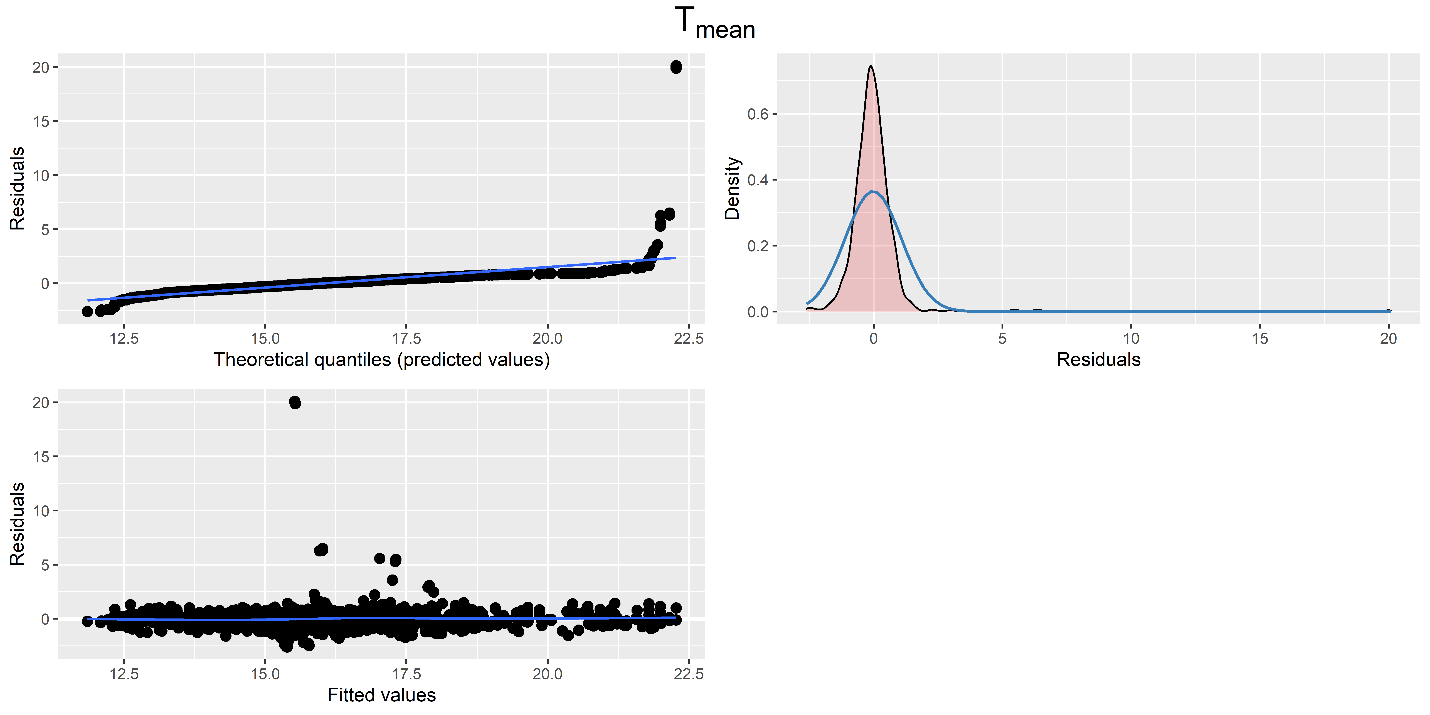

**Figure S26: Model diagnostics for the monthly average of the daily mean temperature for the *summer* season. Residuals are for the *full model* with *unscaled* variables. Please refer to the Methods section in the main text for an explanation of each metric.**


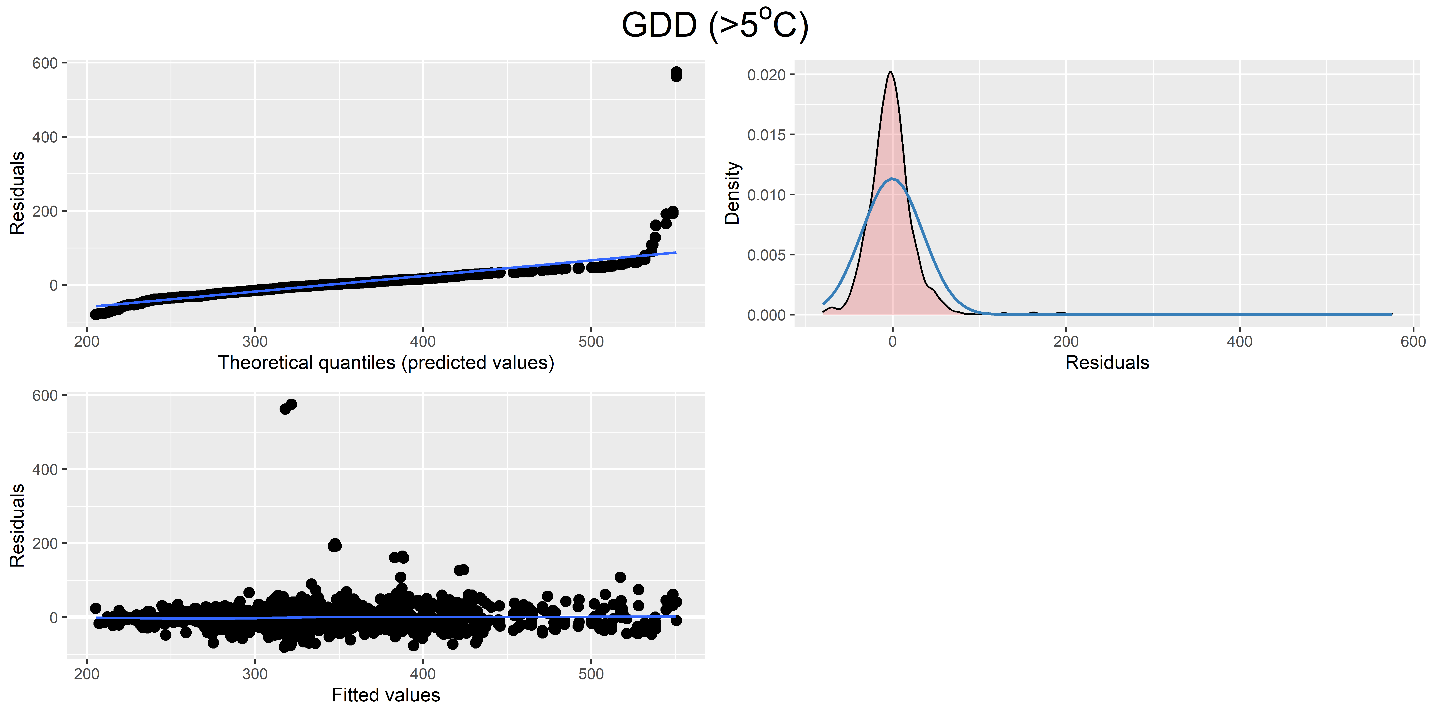
 **Figure S27: Model diagnostics for the monthly average growing degree days above 5 ^o^C for the *summer* season. Residuals are for the *full model* with *unscaled* variables. Please refer to the Methods section in the main text for an explanation of each metric.**


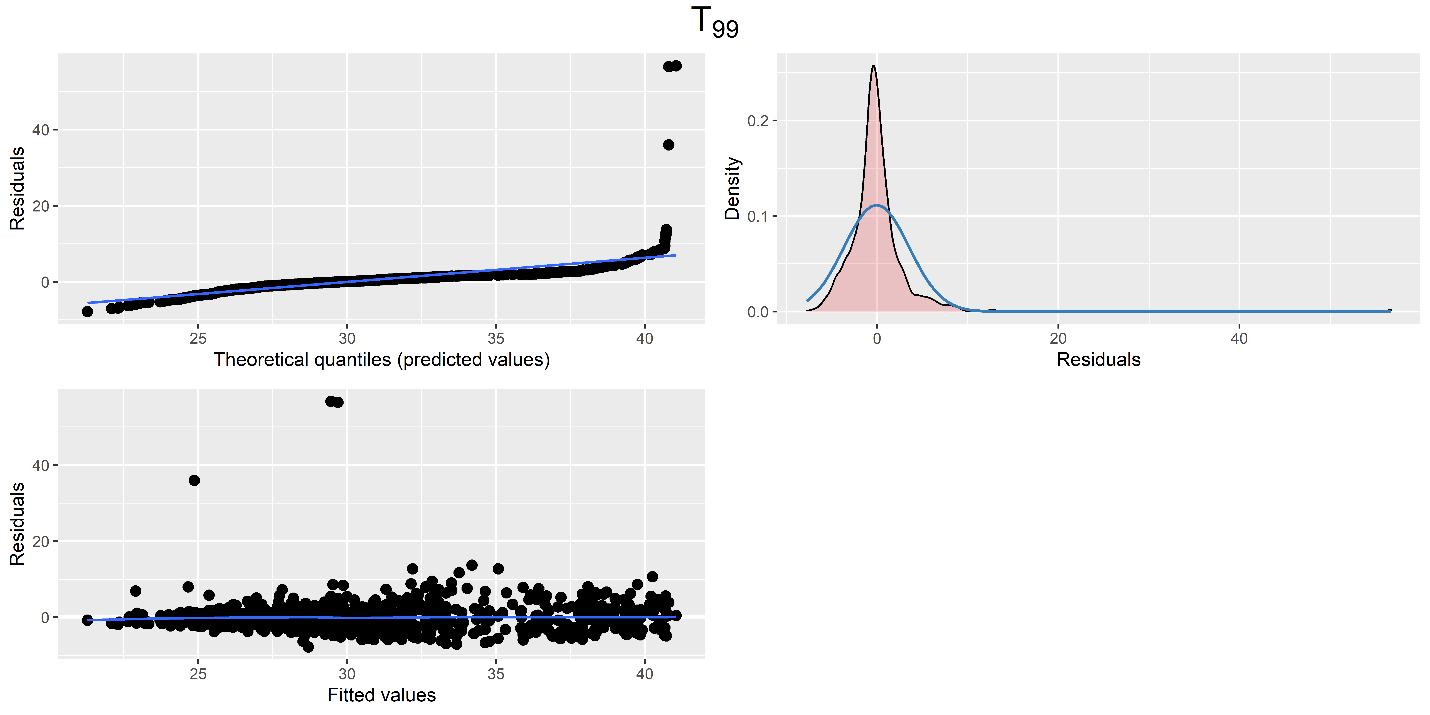

 **Figure S28: Model diagnostics for the 99^th^ percentile of daily maxima for the *summer* season. Residuals are for the *full model* with *unscaled* variables. Please refer to the Methods section in the main text for an explanation of each metric.**


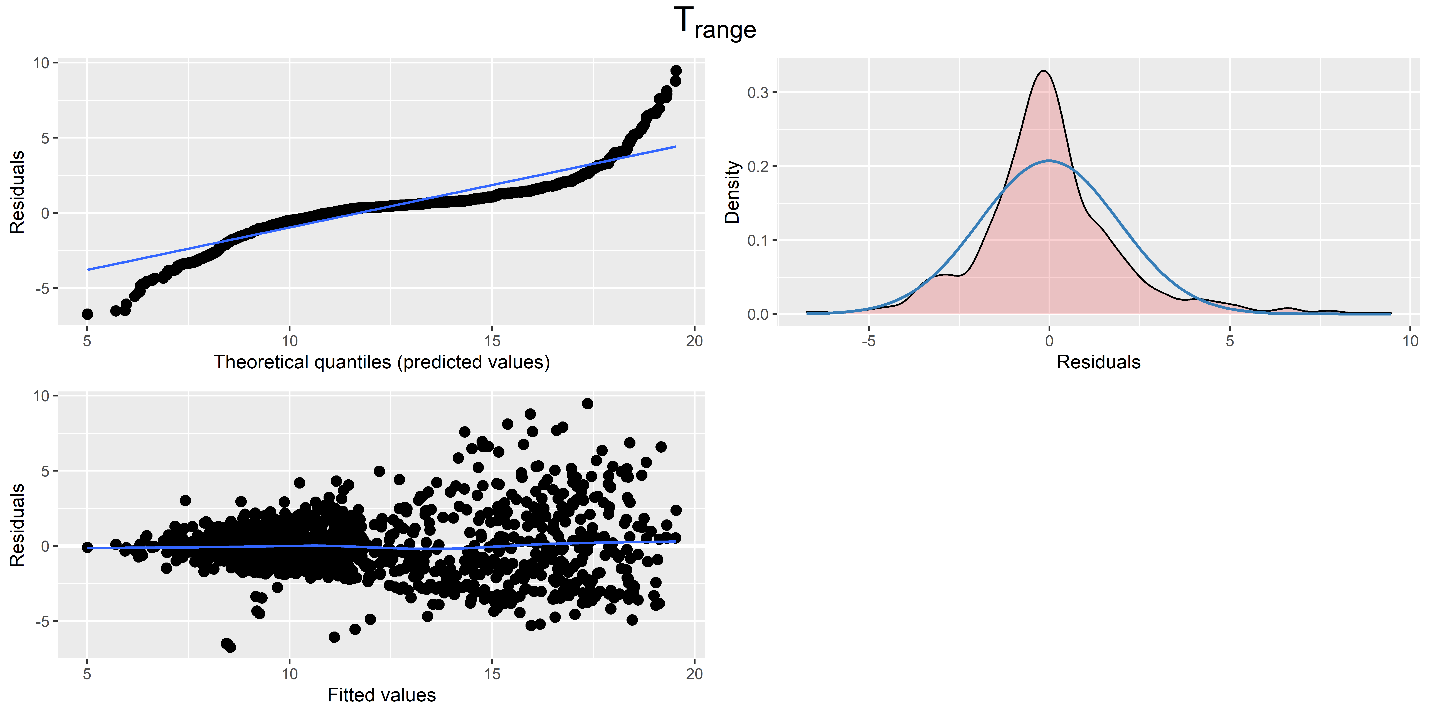
 **Figure S29: Model diagnostics for the monthly average of daily temperature range for the *summer* season. Residuals are for the *full model* with *unscaled* variables. Please refer to the Methods section in the main text for an explanation of each metric.**


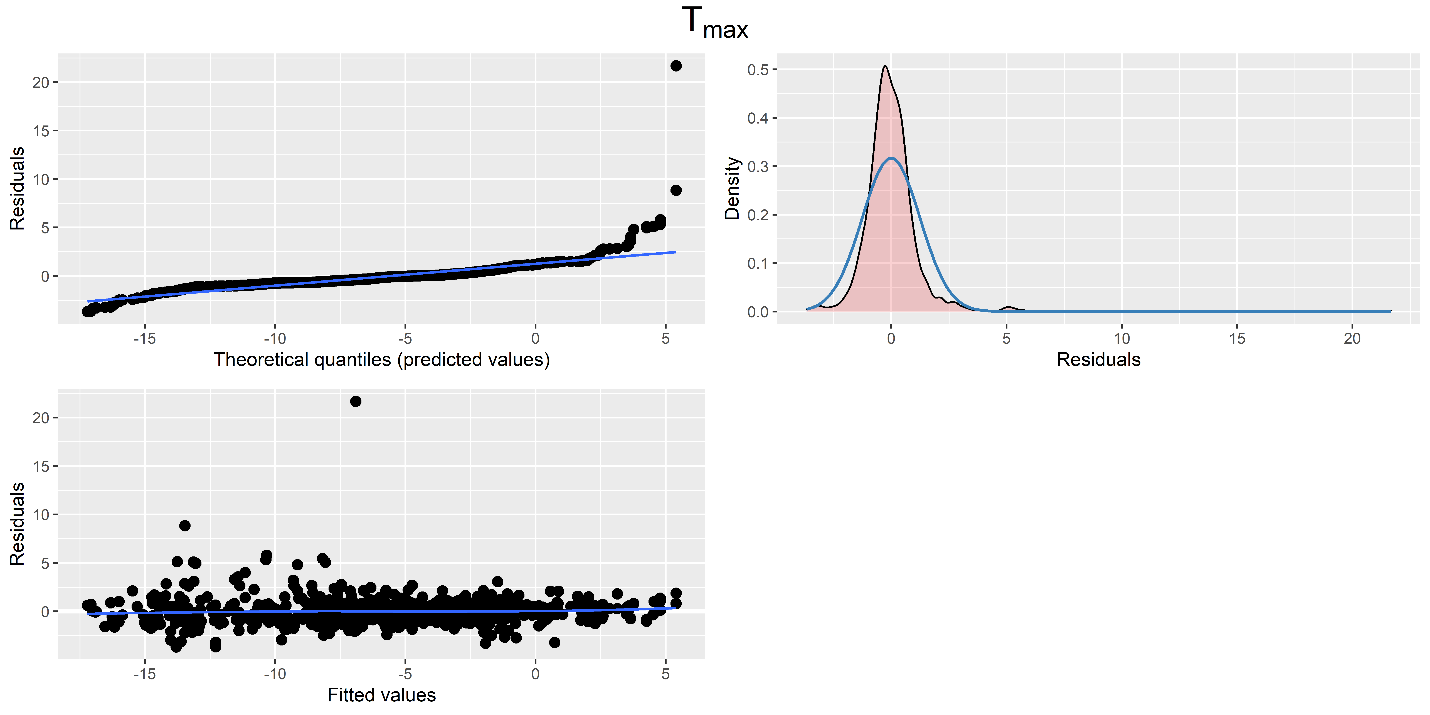

**Figure S30: Model diagnostics for the monthly average of the daily maximum temperature for the *winter* season. Residuals are for the *full model* with *unscaled* variables. Please refer to the Methods section in the main text for an explanation of each metric.**


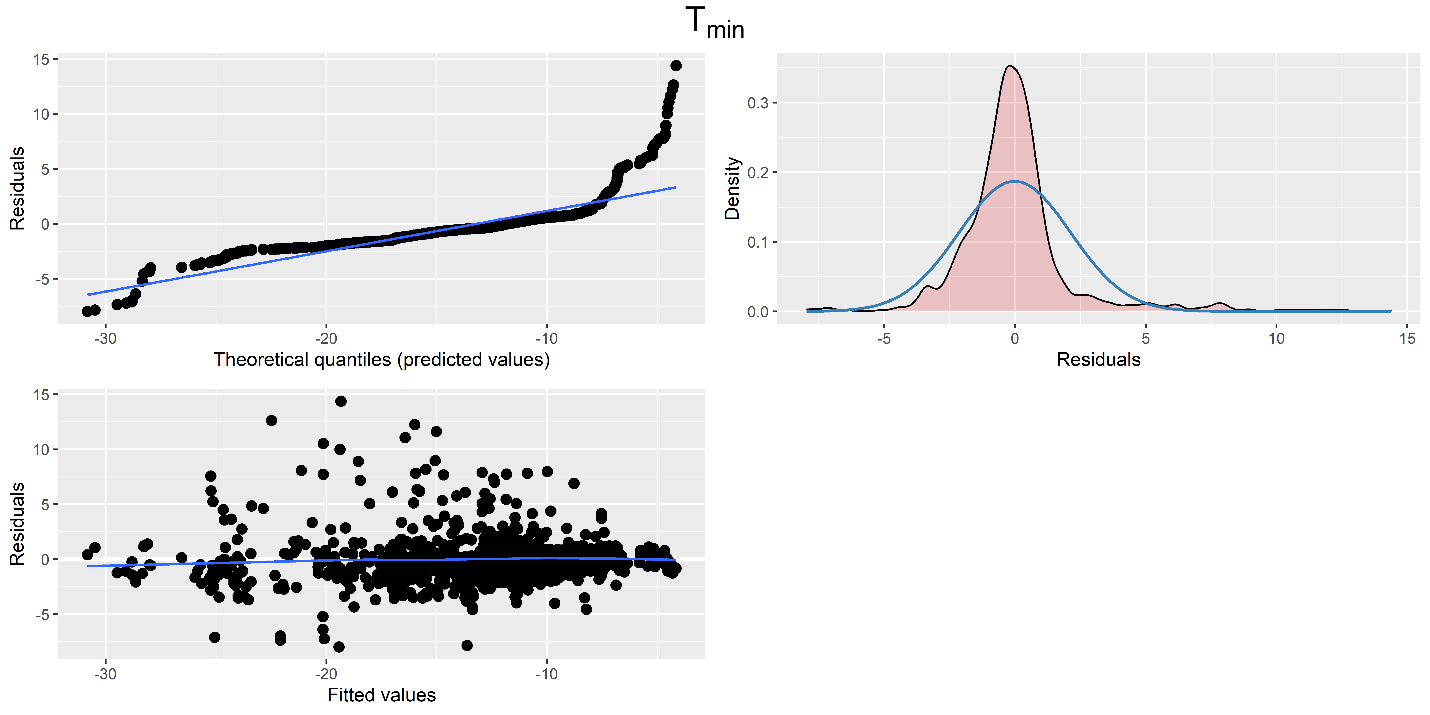

**Figure S31: Model diagnostics for the monthly average of the daily minimum temperature for the *winter* season. Residuals are for the *full model* with *unscaled* variables. Please refer to the Methods section in the main text for an explanation of each metric.**


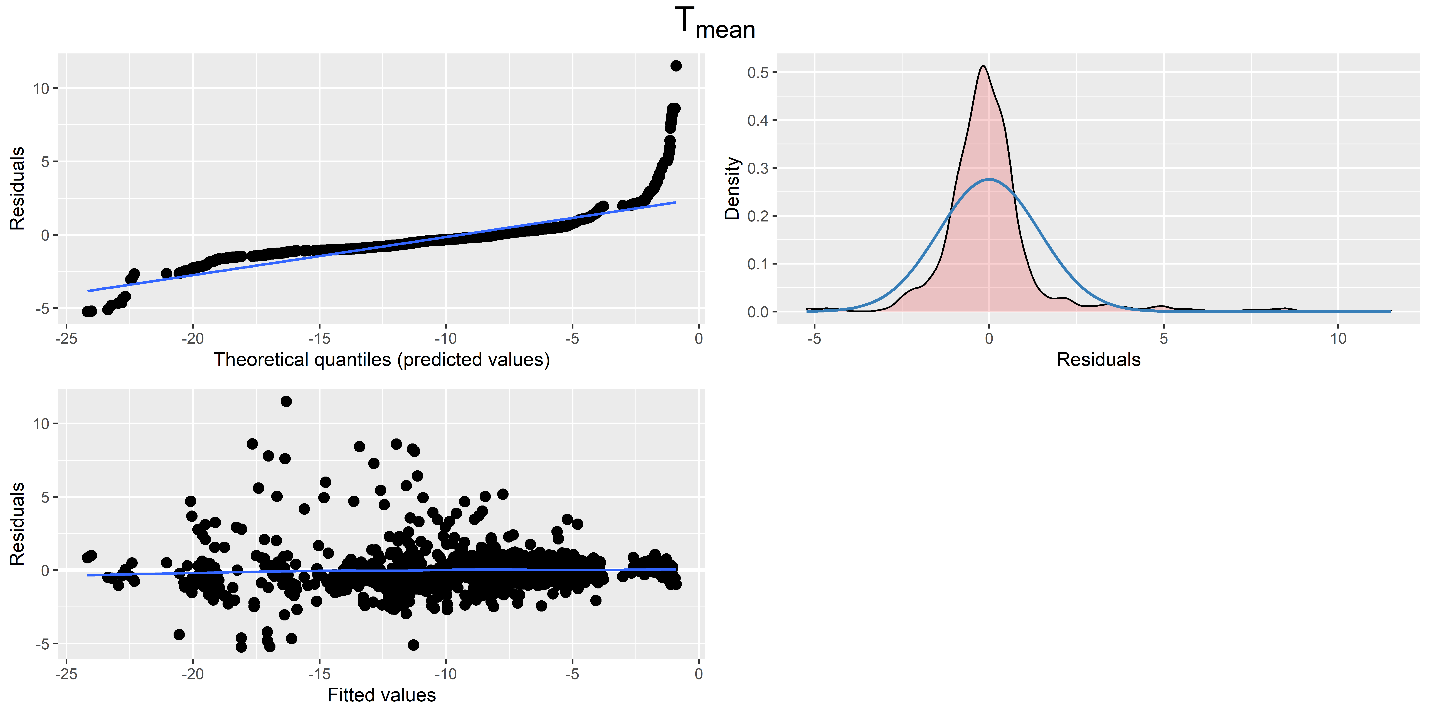


**Figure S32: Model diagnostics for the monthly average of the daily mean temperature for the *winter* season. Residuals are for the *full model* with *unscaled* variables. Please refer to the Methods section in the main text for an explanation of each metric.**


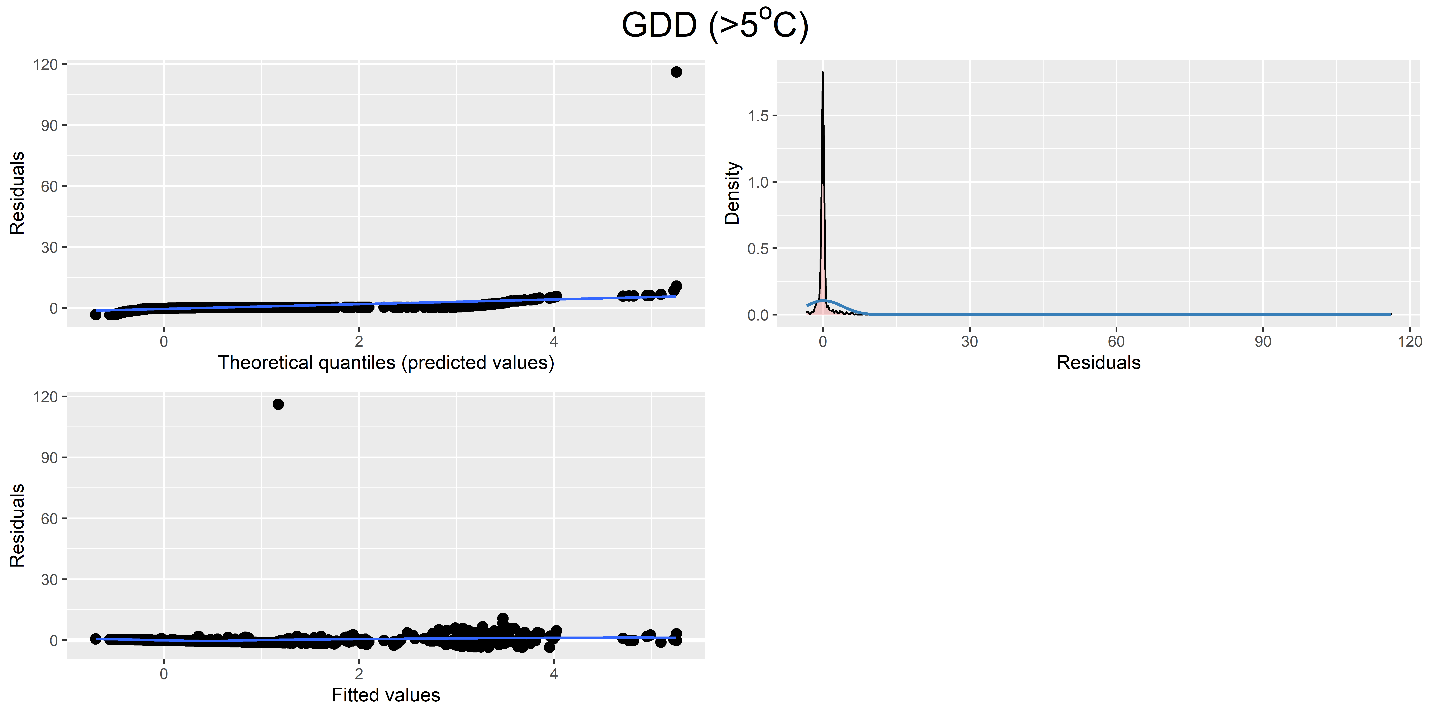


**Figure S33: Model diagnostics for the monthly average growing degree days above 5 ^o^C for the *winter* season. Residuals are for the *full model* with *unscaled* variables. Please refer to the Methods section in the main text for an explanation of each metric.**


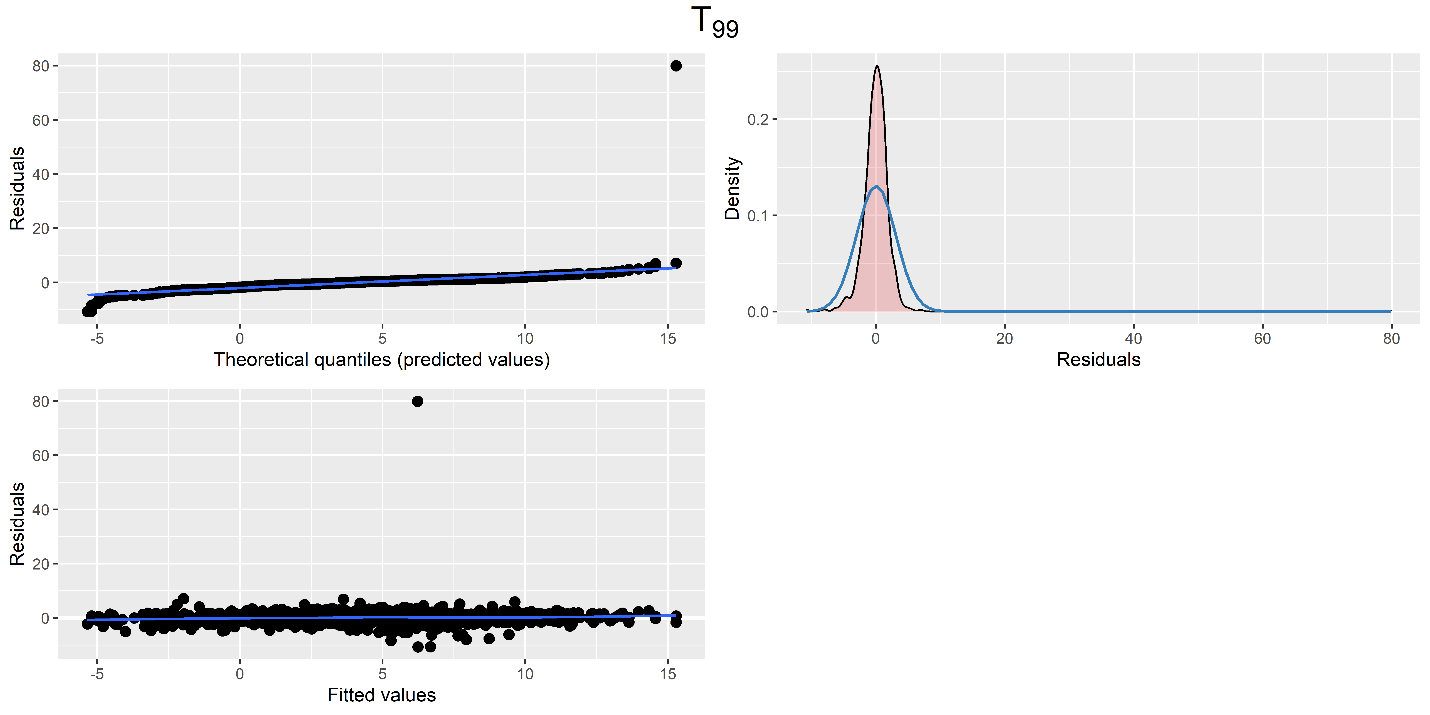


**Figure S34: Model diagnostics for the 99^th^ percentile of daily maxima for the *winter* season. Residuals are for the *full model* with *unscaled* variables. Please refer to the Methods section in the main text for an explanation of each metric.**


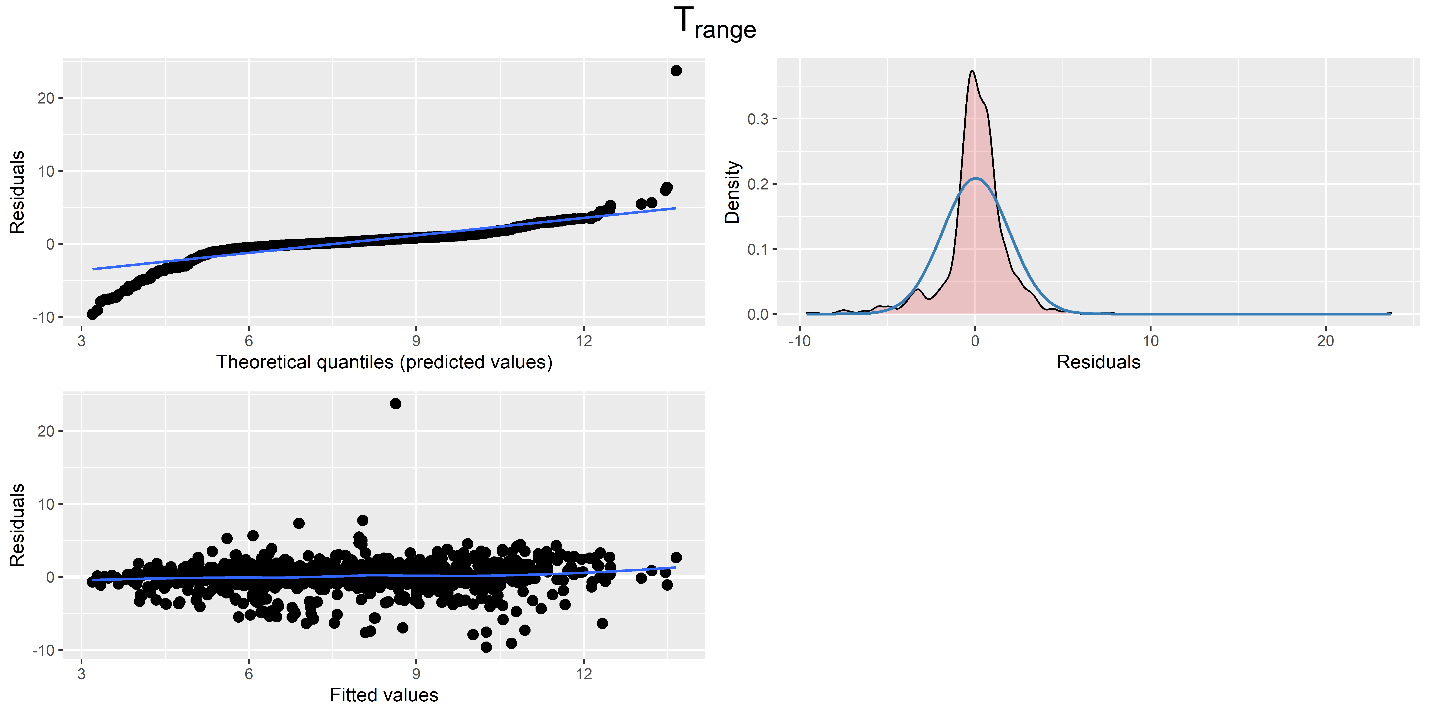
 **Figure S35: Model diagnostics for the monthly average of daily temperature range for the *winter* season. Residuals are for the *full model* with *unscaled* variables. Please refer to the Methods section in the main text for an explanation of each metric.**


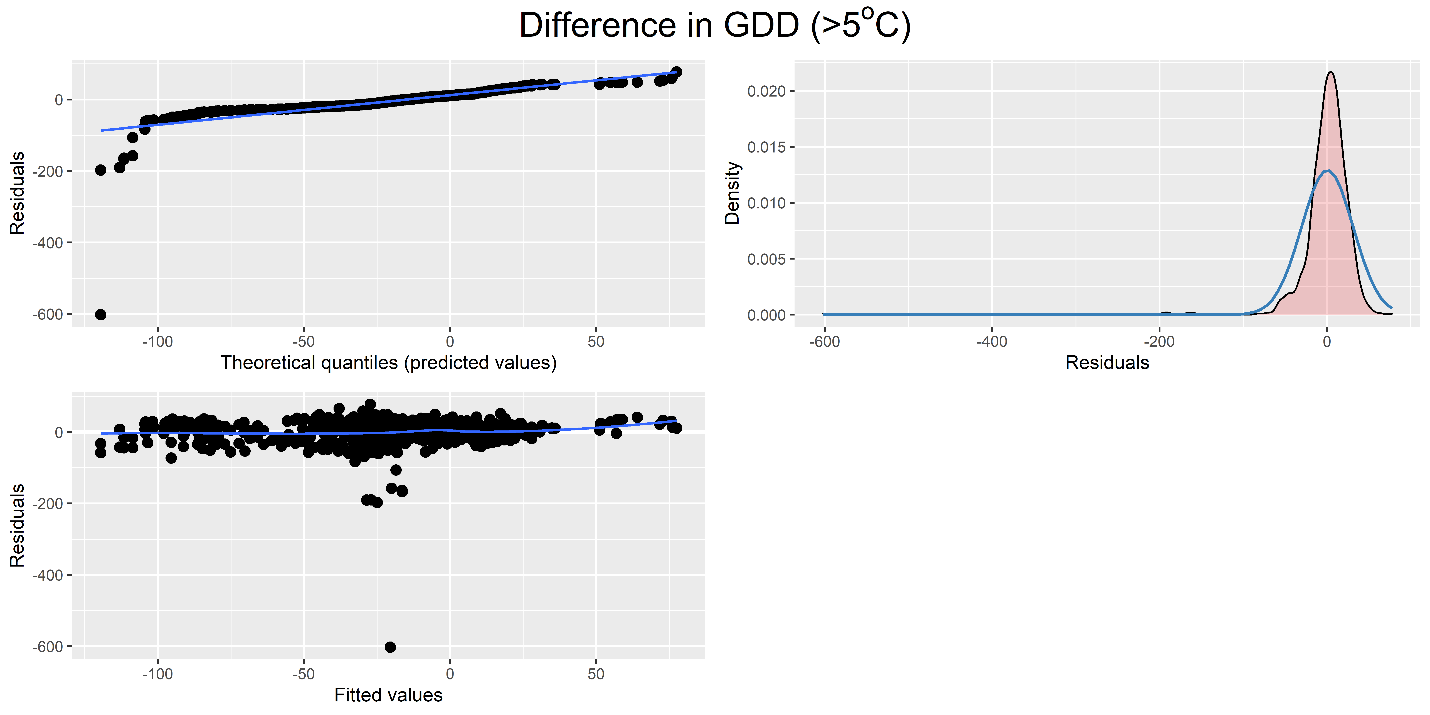

**Figure S36: Model diagnostics for the absolute difference in monthly average growing degree days above 5 ^o^C between ClimateNA and iButton readings (T_Difference_ = T_ClimateNA_ - T_iButton_) for the *summer* season in hill and valley systems in Alberta, Canada. Residuals are for the *full model* with *scaled* variables.**


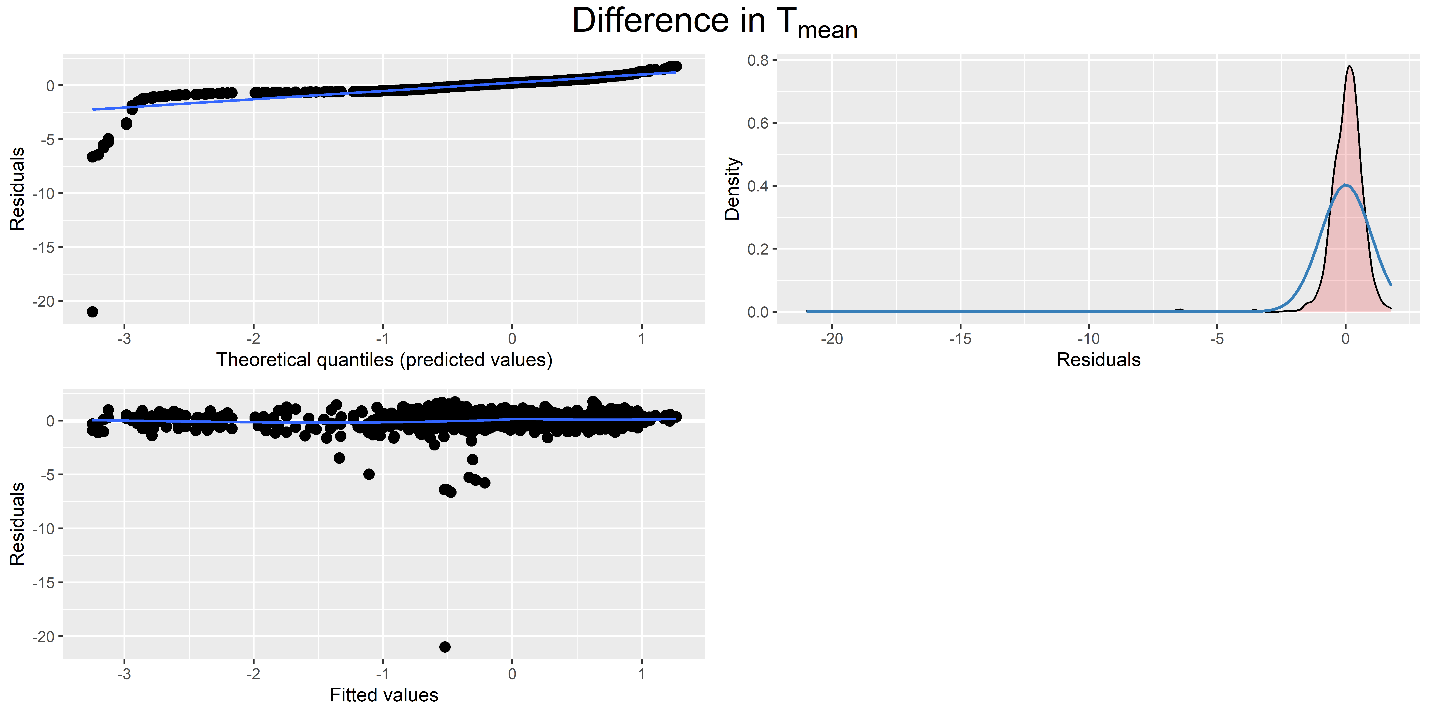

**Figure S37: Model diagnostics for the absolute difference in monthly average of the daily mean temperature between ClimateNA and iButton readings (T_Difference_ = T_ClimateNA_ - T_iButton_) for the *summer* season in hill and valley systems in Alberta, Canada. Residuals are for the *full model* with *scaled* variables.**


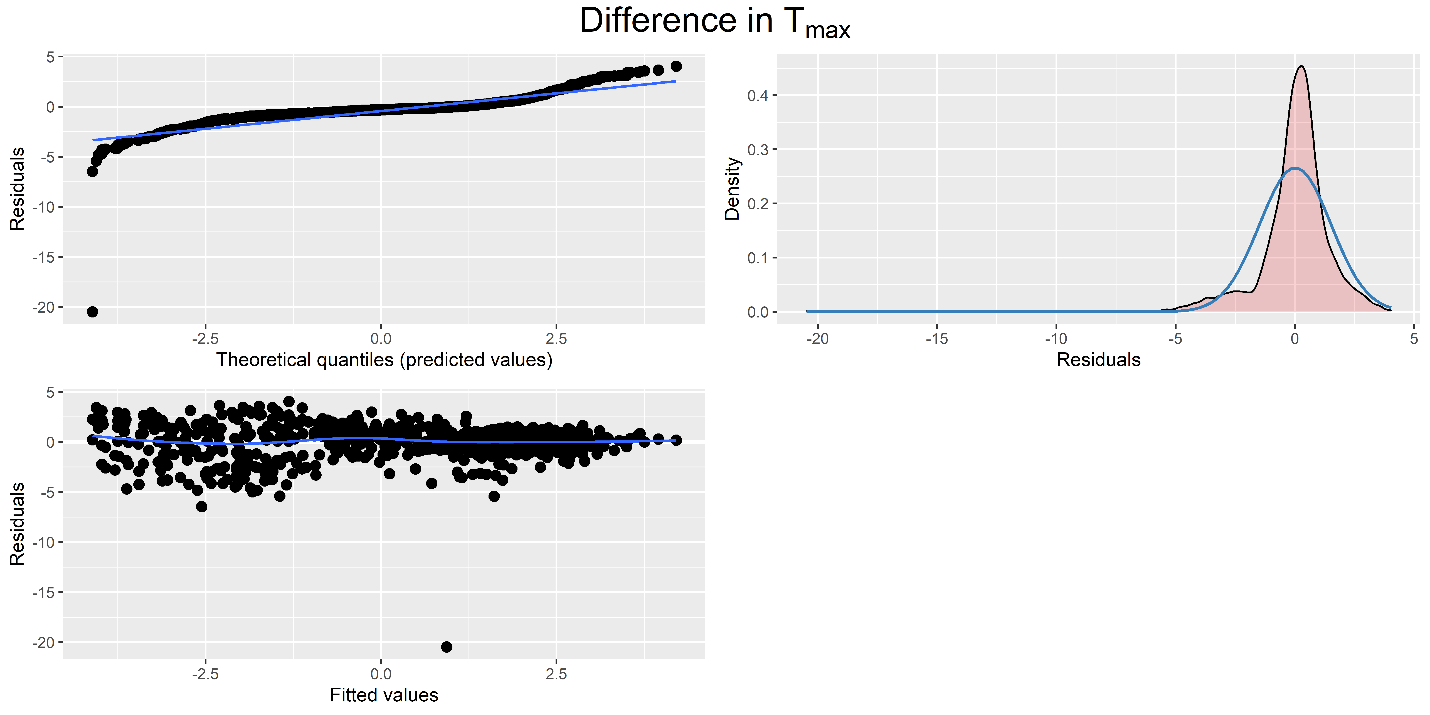

**Figure S38: Model diagnostics for the absolute difference in monthly average of the daily maxima temperature between ClimateNA and iButton readings (T_Difference_ = T_ClimateNA_ - T_iButton_) for the *summer* season in hill and valley systems in Alberta, Canada. Residuals are for the *full model* with *scaled* variables.**


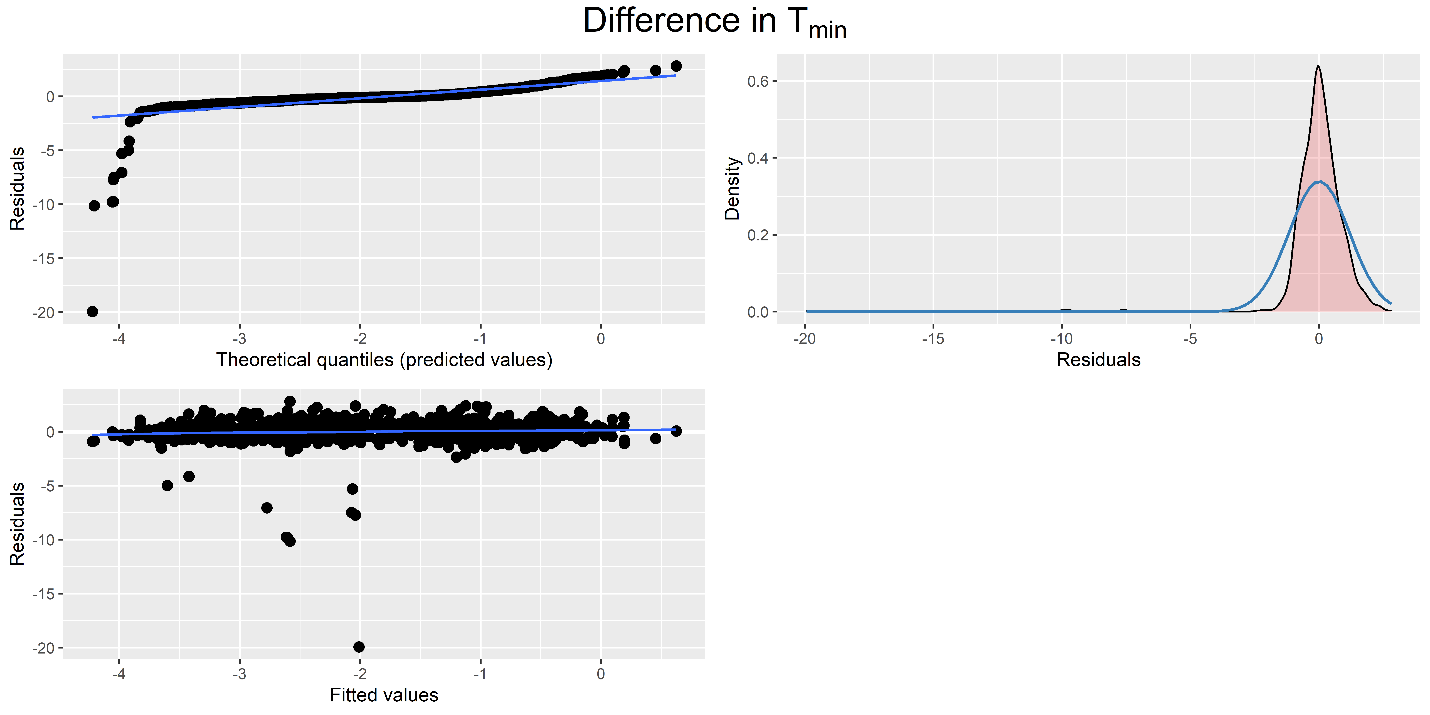


**Figure S39: Model diagnostics for the absolute difference in monthly average of the daily minimum temperature between ClimateNA and iButton readings (T_Difference_ = T_ClimateNA_ - T_iButton_) for the *summer* season in hill and valley systems in Alberta, Canada. Residuals are for the *full model* with *scaled* variables.**


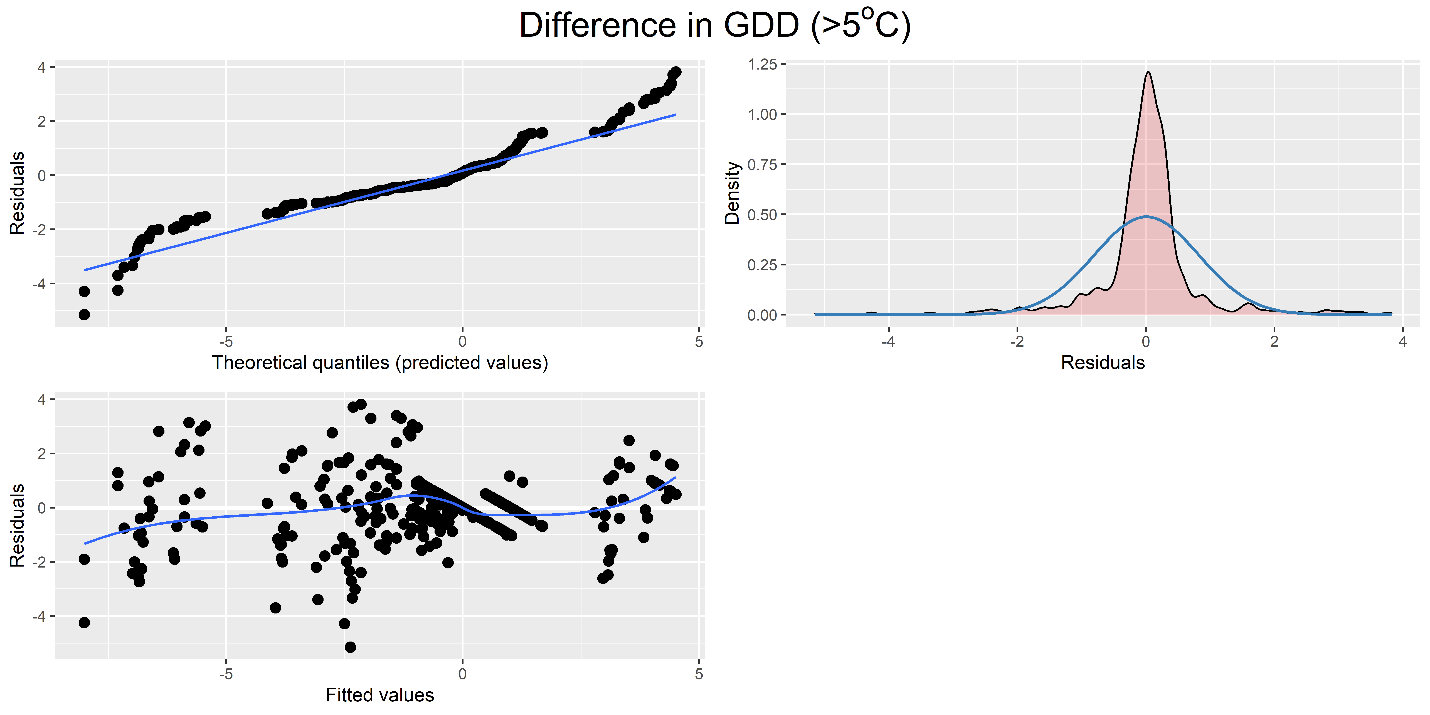

**Figure S40: Model diagnostics for the absolute difference in monthly average growing degree days above 5 ^o^C between ClimateNA and iButton readings (T_Difference_ = T_ClimateNA_ - T_iButton_) for the *winter* season in hill and valley systems in Alberta, Canada. Residuals are for the *full model* with *scaled* variables.**


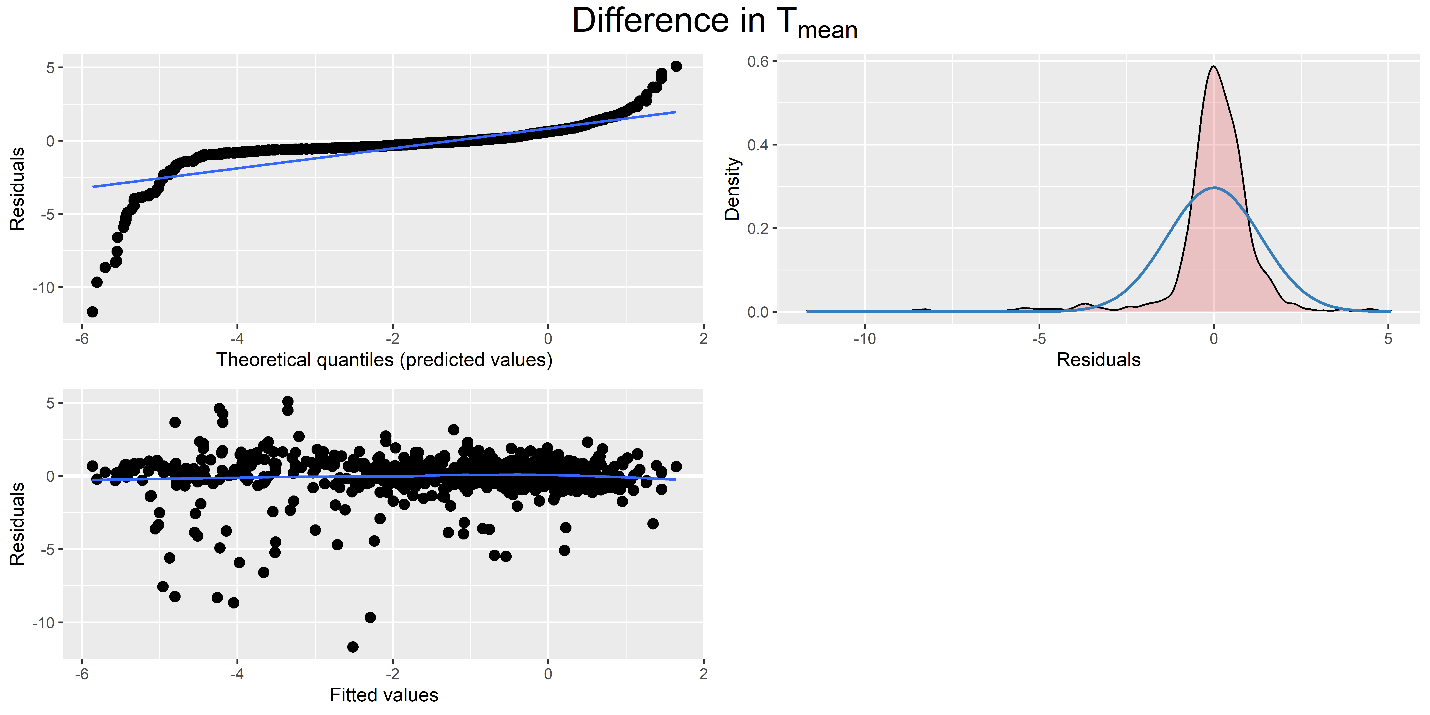

**Figure S41: Model diagnostics for the absolute difference in monthly average of the daily mean temperature between ClimateNA and iButton readings (T_Difference_ = T_ClimateNA_ - T_iButton_) for the *winter* season in hill and valley systems in Alberta, Canada. Residuals are for the *full model* with *scaled* variables.**


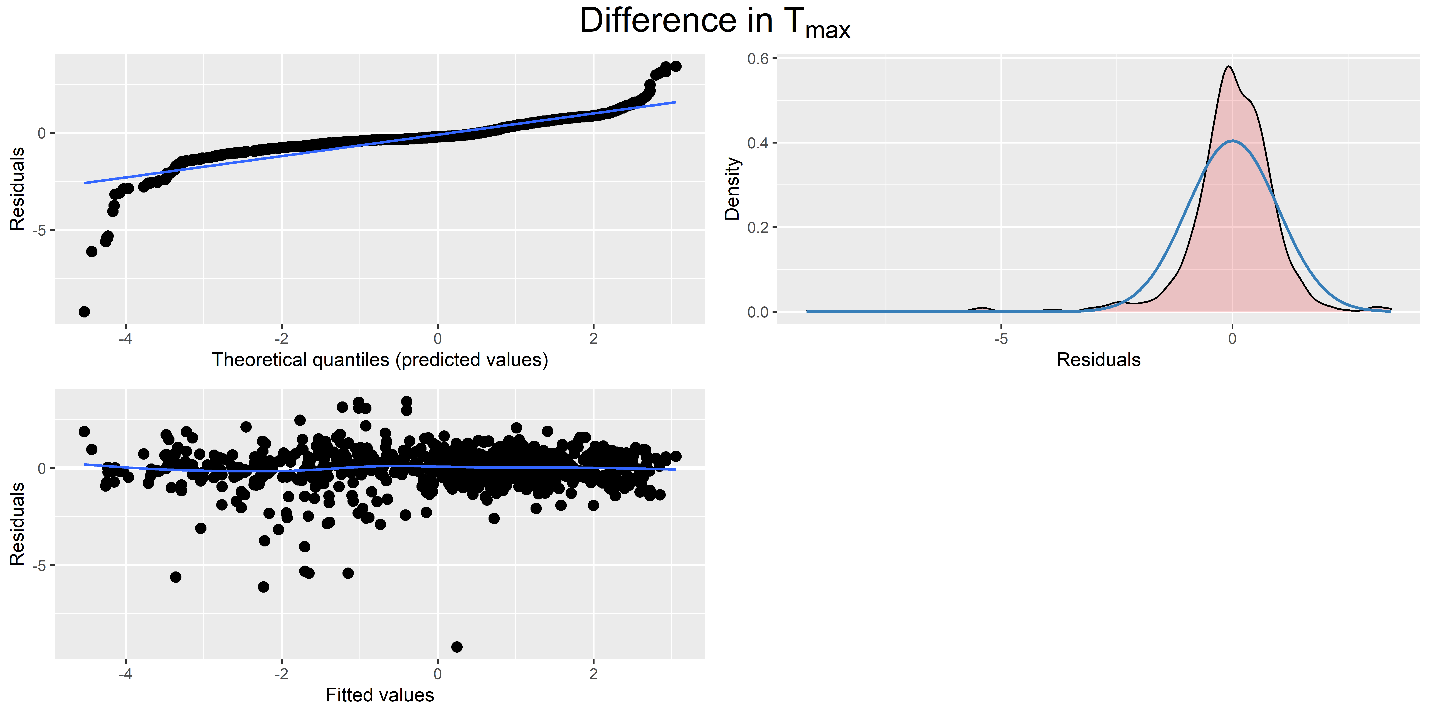

**Figure S42: Model diagnostics for the absolute difference in monthly average of the daily maxima temperature between ClimateNA and iButton readings (T_Difference_ = T_ClimateNA_ - T_iButton_) for the *winter* season in hill and valley systems in Alberta, Canada. Residuals are for the *full model* with *scaled* variables.**


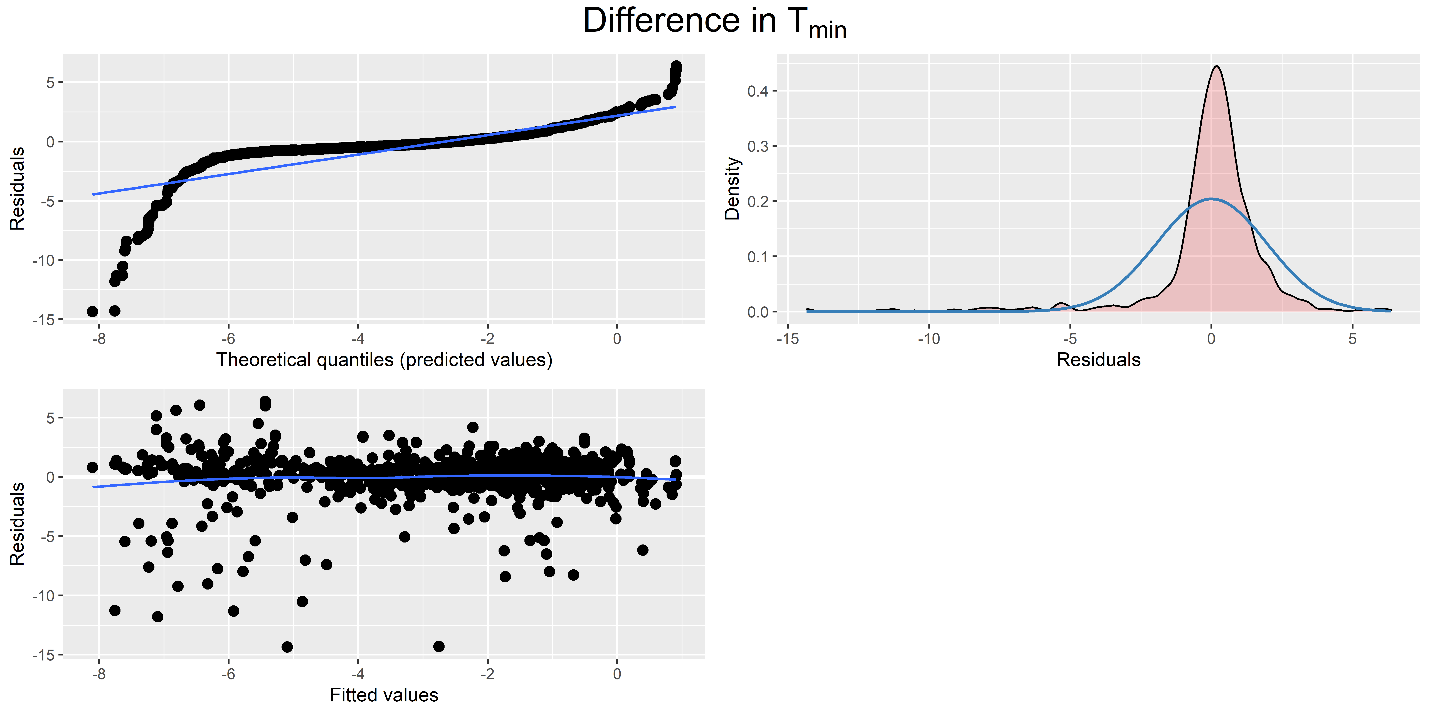
 **Figure S43: Model diagnostics for the absolute difference in monthly average of the daily minimum temperature between ClimateNA and iButton readings (T_Difference_ = T_ClimateNA_ - T_iButton_) for the *winter* season in hill and valley systems in Alberta, Canada. Residuals are for the *full model* with *scaled* variables.**


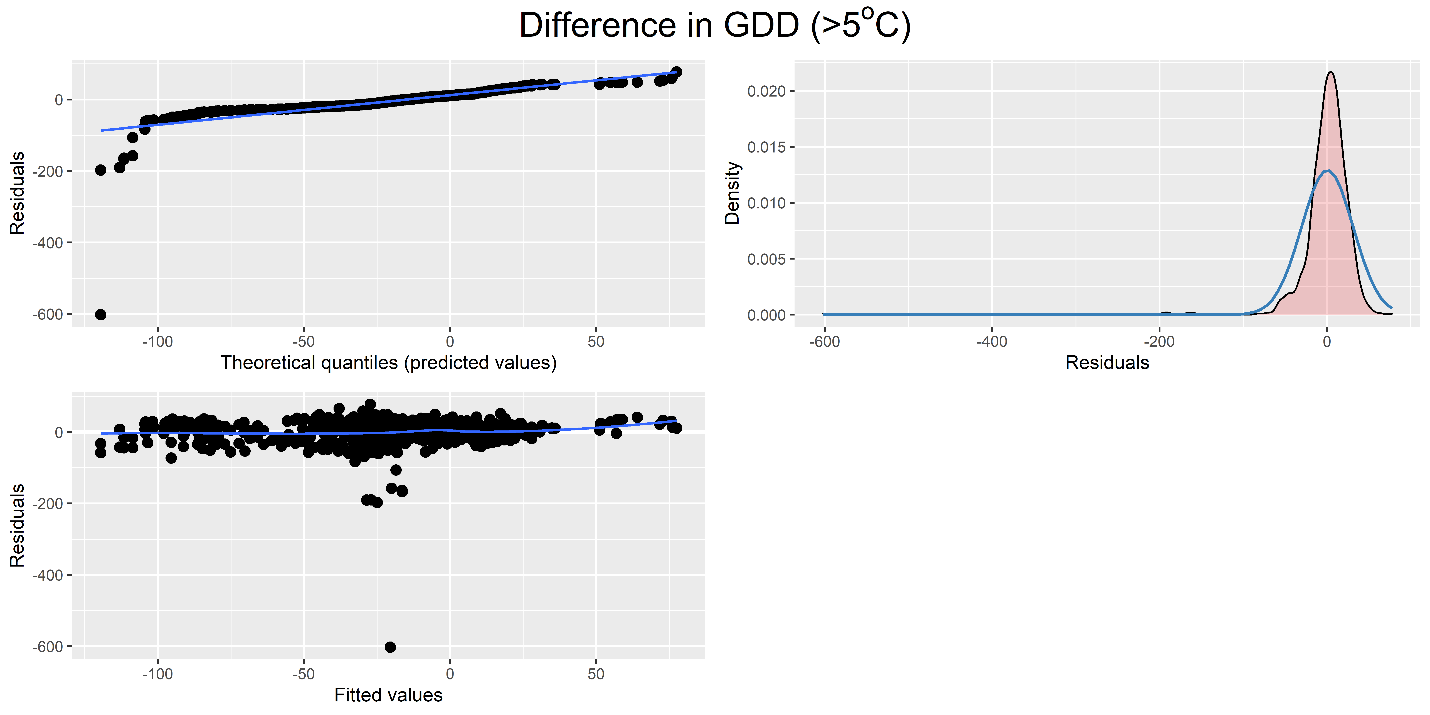

**Figure S44: Model diagnostics for the absolute difference in monthly average growing degree days above 5 ^o^C between ClimateNA and iButton readings (T_Difference_ = T_ClimateNA_ - T_iButton_) for the *summer* season in hill and valley systems in Alberta, Canada. Residuals are for the *full model* with *unscaled* variables.**


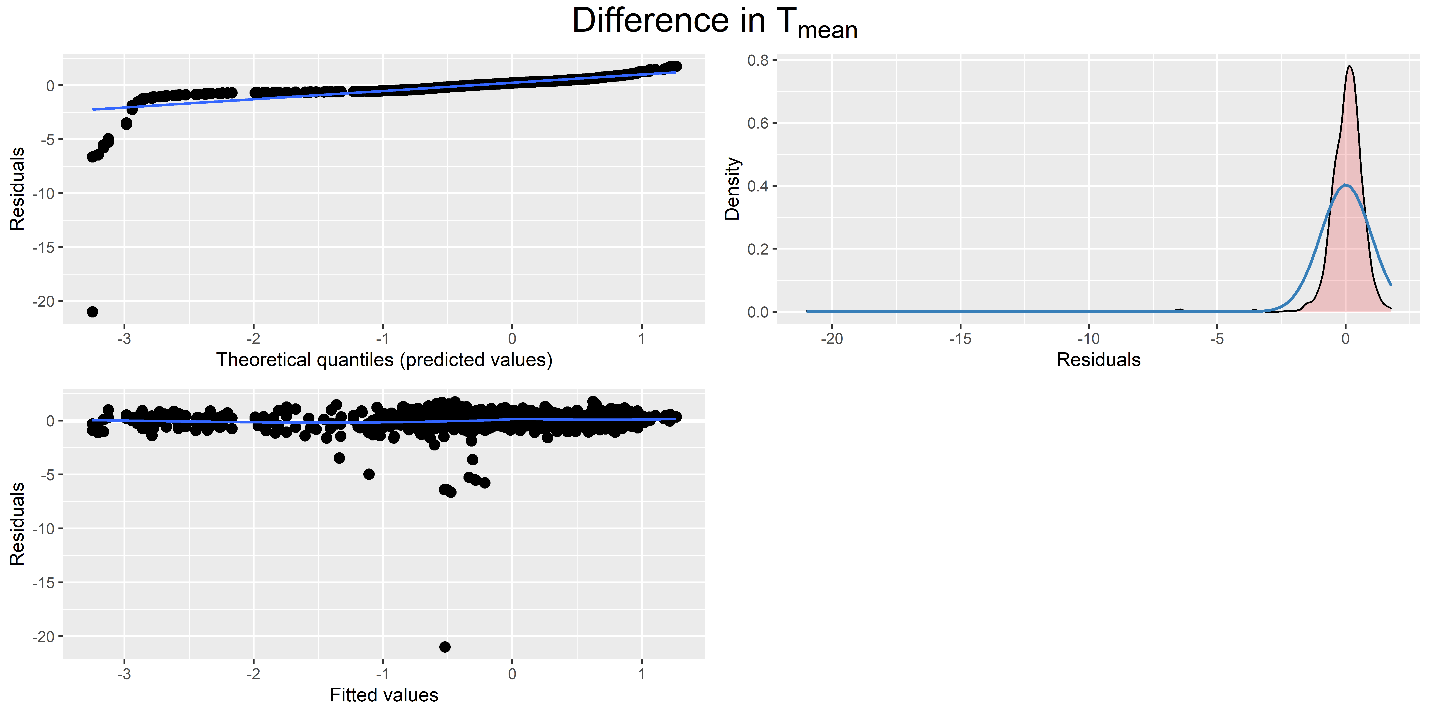

**Figure S45: Model diagnostics for the absolute difference in monthly average of the daily mean temperature between ClimateNA and iButton readings (T_Difference_ = T_ClimateNA_ - T_iButton_) for the *summer* season in hill and valley systems in Alberta, Canada. Residuals are for the *full model* with *unscaled* variables.**


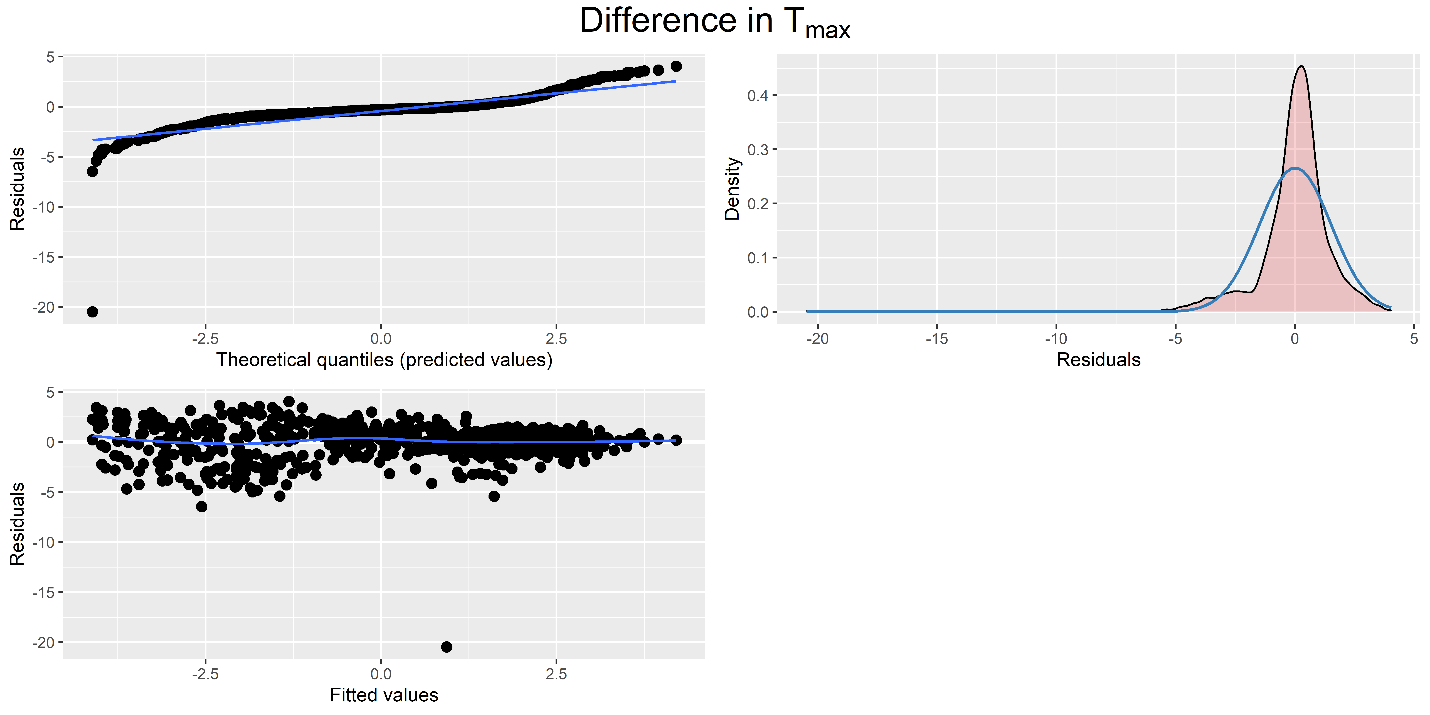

**Figure S46: Model diagnostics for the absolute difference in monthly average of the daily maxima temperature between ClimateNA and iButton readings (T_Difference_ = T_ClimateNA_ - T_iButton_) for the *summer* season in hill and valley systems in Alberta, Canada. Residuals are for the *full model* with *unscaled* variables.**


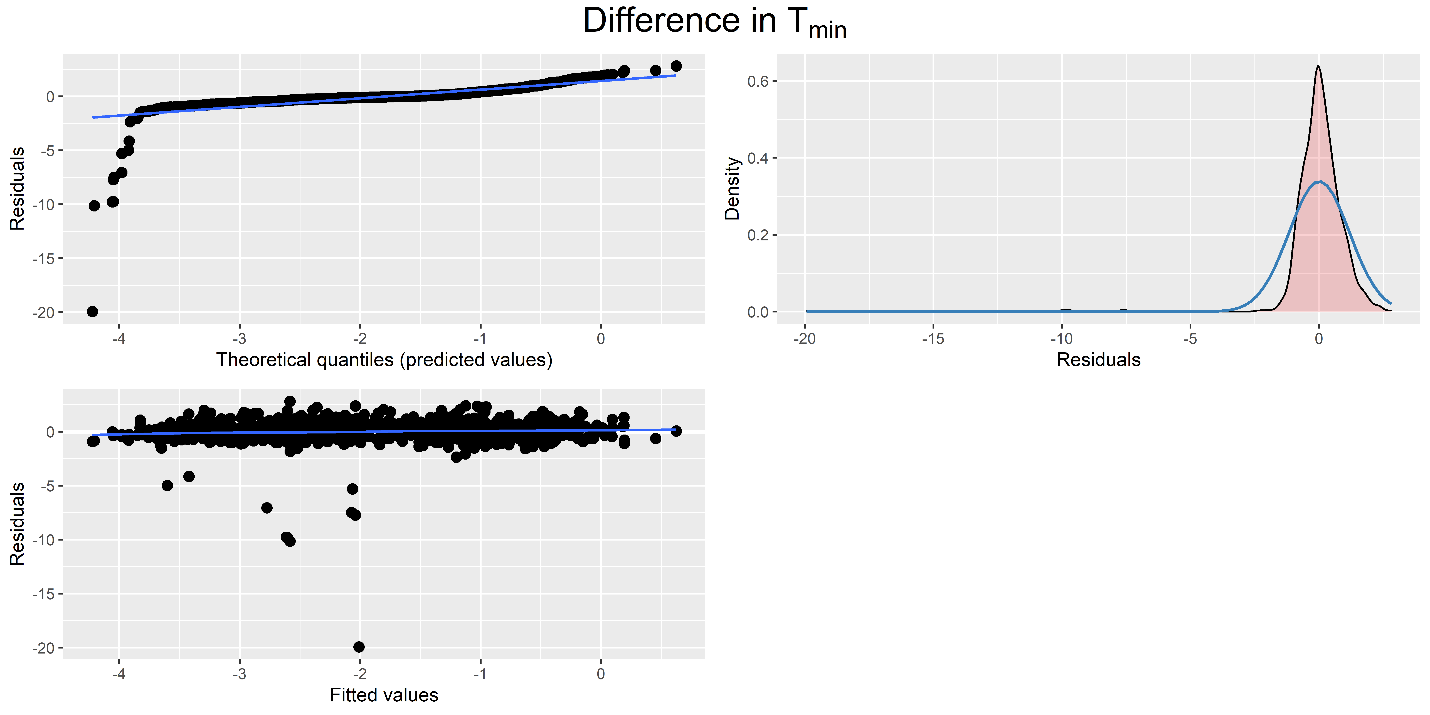
 **Figure S47: Model diagnostics for the absolute difference in monthly average of the daily minimum temperature between ClimateNA and iButton readings (T_Difference_ = T_ClimateNA_ - T_iButton_) for the *summer* season in hill and valley systems in Alberta, Canada. Residuals are for the *full model* with *unscaled* variables.**.


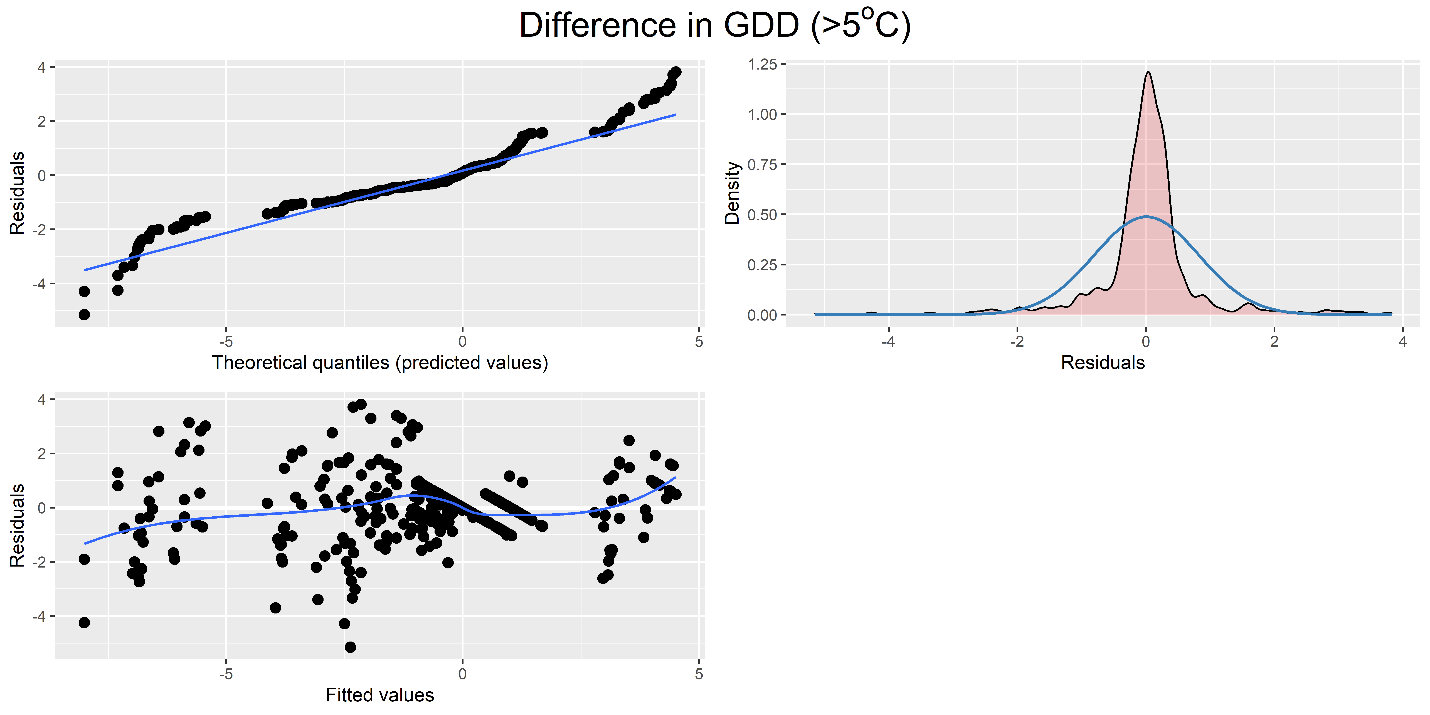

**Figure S48: Model diagnostics for the absolute difference in monthly average growing degree days above 5 ^o^C between ClimateNA and iButton readings (T_Difference_ = T_ClimateNA_ - T_iButton_) for the *winter* season in hill and valley systems in Alberta, Canada. Residuals are for the *full model* with *unscaled* variables.**


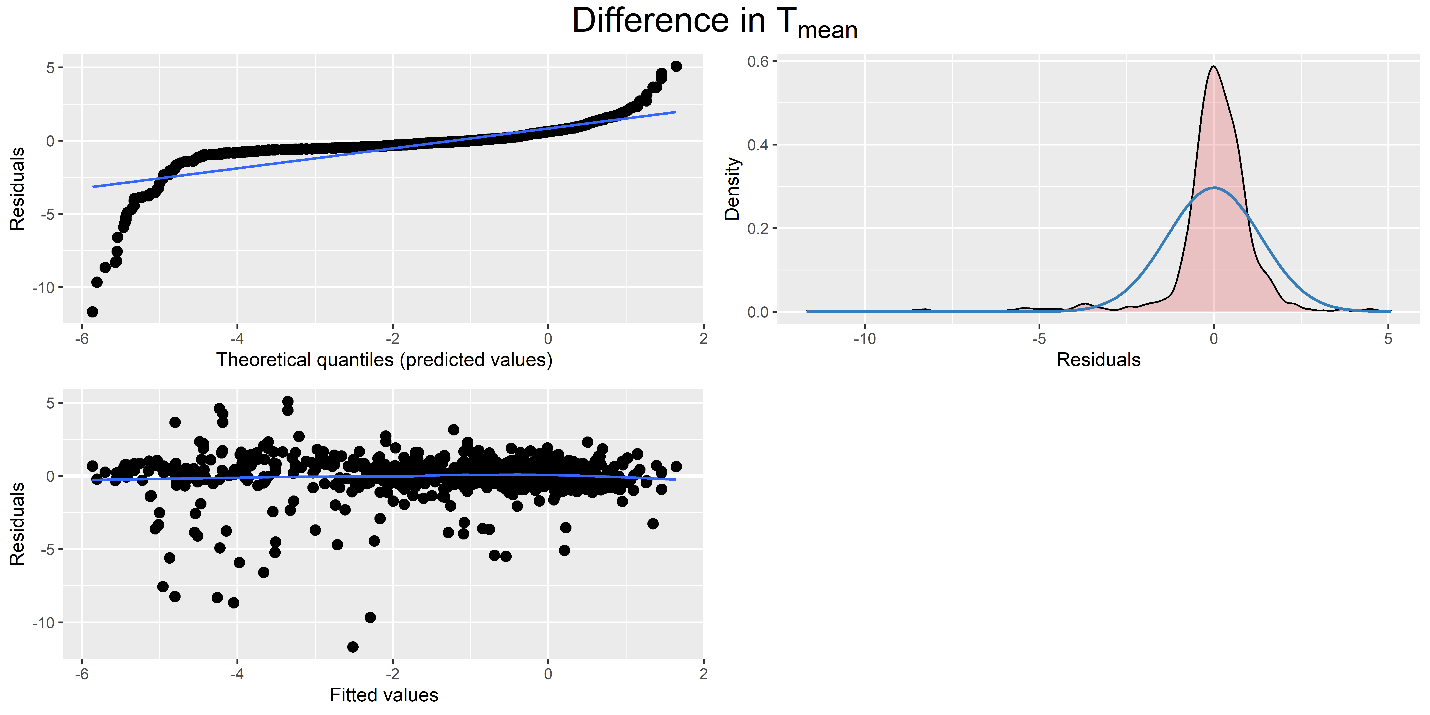

**Figure S49: Model diagnostics for the absolute difference in monthly average of the daily mean temperature between ClimateNA and iButton readings (T_Difference_ = T_ClimateNA_ - T_iButton_) for the *winter* season in hill and valley systems in Alberta, Canada. Residuals are for the *full model* with *unscaled* variables.**


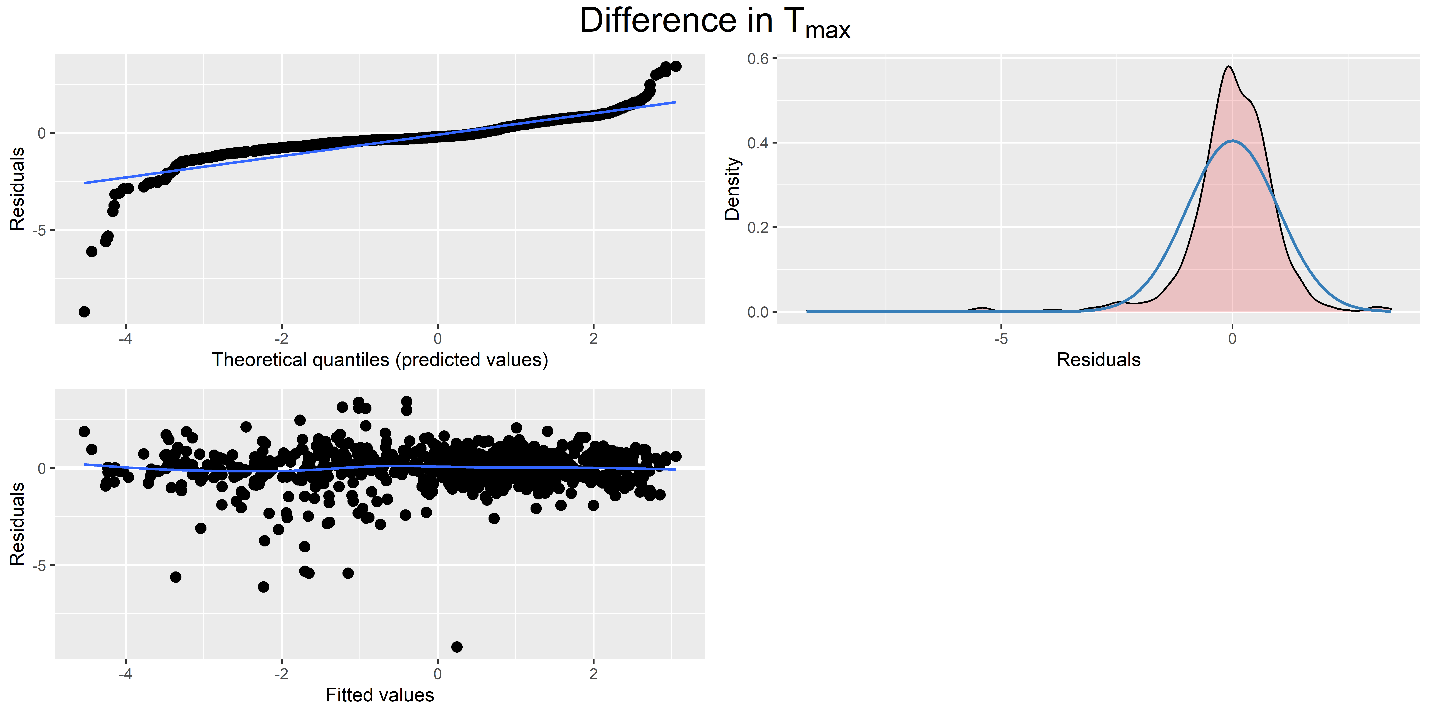

**Figure S50: Model diagnostics for the absolute difference in monthly average of the daily maxima temperature between ClimateNA and iButton readings (T_Difference_ = T_ClimateNA_ - T_iButton_) for the *winter* season in hill and valley systems in Alberta, Canada. Residuals are for the *full model* with *unscaled* variables.**


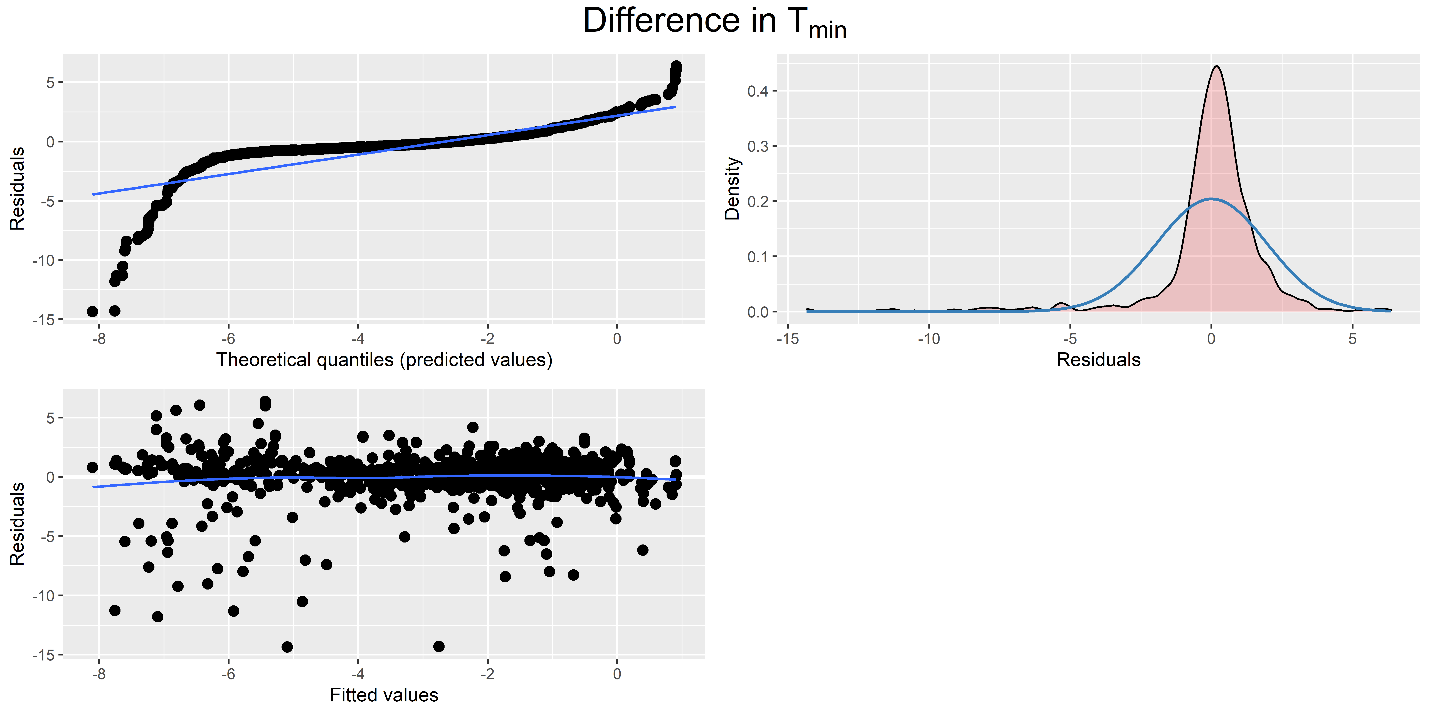

**Figure S51: Model diagnostics for the absolute difference in monthly average of the daily minimum temperature between ClimateNA and iButton readings (T_Difference_ = T_ClimateNA_ - T_iButton_) for the *winter* season in hill and valley systems in Alberta, Canada. Residuals are for the *full model* with *unscaled* variables.**
